# Catalyst- and solvent-free approach to 2-arylated quinolines *via* [5 + 1] annulation of 2-methylquinolines with diynones†

**DOI:** 10.1039/c7ra12716b

**Published:** 2018-01-25

**Authors:** Hai-Yuan Zhao, Fu-Song Wu, Li Yang, Ying Liang, Xiao-Lin Cao, Heng-Shan Wang, Ying-Ming Pan

**Affiliations:** School of Life and Environmental Sciences, Guilin University of Electronic Technology Guilin 541004 China yingl@aliyun.com; State Key Laboratory for Chemistry and Molecular Engineering of Medicinal Resources, School of Chemistry and Pharmaceutical Sciences of Guangxi Normal University Guilin 541004 China panym@mailbox.gxnu.edu.cn +86-773-5803930; Guangxi Key Laboratory of Special Non-wood Forest Cultivation and Utilization, Guangxi Zhuang Autonomous Region Forestry Research Institute Nanning 530002 China

## Abstract

A novel route for the synthesis of 2-arylated quinolines through a [5 + 1] annulation directly from 2-methylquinolines and diynones under catalyst-free and solvent-free conditions was disclosed. This synthetic process was atom-economic, with good tolerance of a broad range of functional groups, and with great practical worth.

## Abstract 2

A novel route for the synthesis of 2-arylated quinolines through a [5 + 1] annulation directly from 2-methylquinolines and diynones under catalyst-free and solvent-free conditions was disclosed.

## Graphical abstract

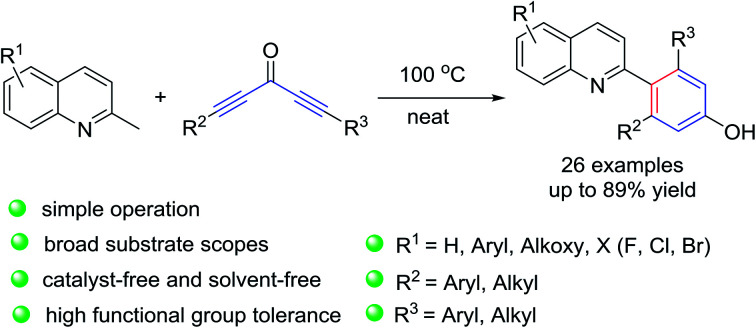

Nitrogen-containing heterocyclic compounds are ubiquitous in natural molecules and exhibit a wide array of biological activities.^1^ Among various N-heterocycles, quinoline nuclei are privileged scaffolds that occupy an important role in many medicinally relevant compounds.^2^ 2-Arylated quinolines are found in many medicinal compounds including etoricoxib,^3^ rosuvastatin,^4^ and gleevec,^5^ as well as molecules designed for other purposes including P, N ligands, such as QUINAP.^6^ Because of their unique biological activity and wide application, the functionalized 2-arylated quinoline elicited considerable synthetic interest, and a variety of synthetic routes have been established.^7^ In addition, some classical synthetic methods such as Kumada,^8^ Suzuki,^9^ Negishi,^10^ or Stille^9*b*^ are usually used to efficiently prepare these compounds, but these methods require the preparation of cross-coupling reagents such as Grignard reagents, boronic acids, organozincs, and organostannanes in advance and these cross-coupling reagents are unstable or toxic or can't be isolated as solids.^11^ More recently, much research has been directed toward the synthesis of 2-arylated quinolones and their derivatives *via* transition-metal-catalyzed C–H arylation^12^ and many other methods also have been developed by transitional-metal-catalyzed cross-couplings.^13^ These transition metals included Co, Cu or Pd. Moreover, 2-arylated quinolines synthesis *via* direct C–H arylation of quinolones with various aryl bromides, arylboronic acid, or arylzinc reagents catalyzed by transition metal catalyst, such as Rh, Fe, or Ni, have been investigated by Bergman,^14^ Maiti,^15^ and Tobisu.^16^ Ru^17^ or Mn^18^ also was used to catalyze indirect Friedländer synthesis to obtain 2-arylated quinolines that involved oxidative cyclization of 2-aminobenzyl alcohol with either ketones or alcohols. Although these processes were highly efficient and significance, none of these procedures could directly provide the final products with a low content of the heavy metal impurities which are strict restrictions in drugs and pharmaceuticals.^19^ Thus, an alternative, general, solvent-free, and environmentally sustainable procedure is urgently required for the quick synthesis of 2-arylated quinolines.

Recently, as our continuous study on the C(sp^3^)–H activation of 2-methylquinolines, which provided a facile synthetic approach to access substituted pyrrolo[1,2-*a*]quinolones (Scheme 1),^20^ we had found that the methyl of 2-methylquinolines has very high reactivity. Based on this, herein we reported the catalyst-free and solvent-free [5 + 1] annulation of 2-methylquinolines and diynones to access 4-(quinolin-2-yl)-phenols. To the best of our knowledge, there were none of the group that reported direct construction of six-member aromatic-ring at the methyl of 2-methylquinolines with diynones to give 4-(quinolin-2-yl)-phenols. The present novel construction protocol for 4-(quinolin-2-yl)-phenols had several significant advantages: (1) this chemistry provided a novel and simple strategy for the synthesis of highly valuable 4-(quinolin-2-yl)-phenols under very simple conditions; (2) according to the atom economy concept, this protocol was carried out under catalyst-free and solvent-free conditions, without the addition of any acid, base, or other reagents, which provided the final products without heavy metal impurities and improved its potential utility; (3) the method featured high functional group tolerance, high yields, and broad substrate scopes. Particularly, this route could directly introduce two different substituent groups on the newly formed of six-member aromatic-ring (see Table 2).

**Scheme 1:**
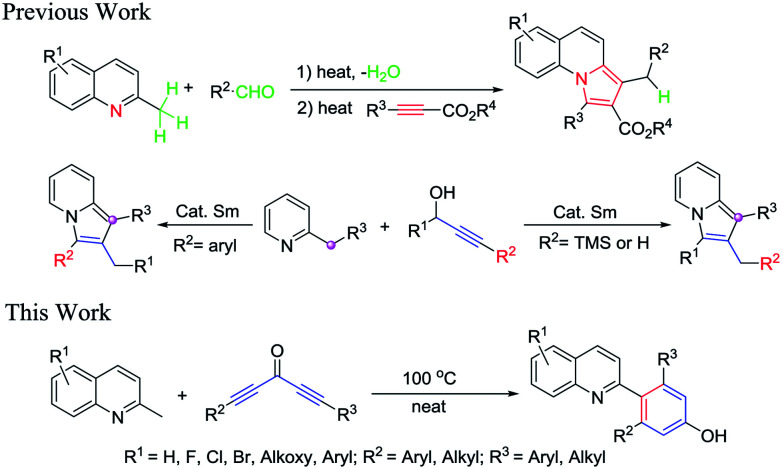
C–H bond activation of 2-methylquinolines and 2-methylpyridine.

To examine the feasibility of our proposed protocol, 2-methylquinoline (1a) and 1,5-diphenylpenta-1,4-diyn-3-one (2a) were chosen as the model substrates and diverse reaction conditions were screened as shown in Table 1. Initially, treatment of 1a (0.6 mmol) with 2a (0.5 mmol) in chlorobenzene (PhCl) at 120 °C for 10 hours led to the arylation product 2′-(quinolin-2-yl)-[1,1′:3′,1′′-terphenyl]-5′-ol (3a) in 65% yield (Table 1, entry 1). The structure of 3a was confirmed by its ^1^H and ^13^C NMR spectra, mass spectra, and single-crystal X-ray diffraction analysis (Fig. 1).^21^ To improve the efficiency, we used Sm(OTf)_3_ as catalyst, but the result provided 3a in less than 60% (Table 1, entry 2). And then, when we used base (Cs_2_CO_3_, Et_3_N) or acid (AcOH) as additives, no desired products was isolated (Table 1, entries 4–6) because of 2a degradation in the presence of acid or base. Subsequently, we used other solvents such as toluene, DMF, DMSO, and 1,4-dioxane in place of PhCl and these reactions were completed in the absence of additives, providing the yields of 3a in less than 65% (Table 1, entries 7–10). Gratifyingly, when the reaction was carried out in the absence of solvent, the yield of corresponding product 3a was increased to 73% (Table 1, entry 11). Subsequently, we carefully adjusted the reaction temperature (Table 1, entries 12–14) and the desired product 3a was obtained in the best yield (85%) when the reaction was performed at 100 °C. The reaction time extended to 15 hours, but the yield of 3a was not increased (Table 1, entry 15).

**Table 1:** Optimization of reaction conditions^a^

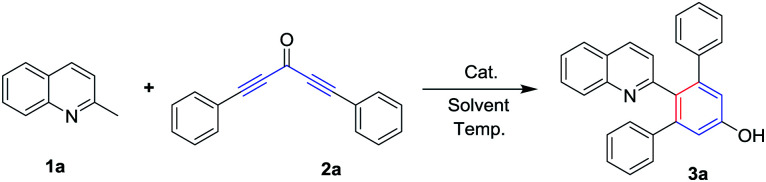				
Entry	Cat.	Solvent	Temp. (°C)	Yield^b^ (%)
1	—	PhCl	120	65
2	Sm(OTf)_3_	PhCl	120	55
3^c^	Sm(OTf)_3_	PhCl	120	0
4	Cs_2_CO_3_	PhCl	120	0
5	Et_3_N	PhCl	120	0
6	AcOH	PhCl	120	0
7	—	Toluene	120	35
8	—	DMF	120	63
9	—	DMSO	120	45
10	—	1,4-Dioxane	120	Trace
11^d^	—	—	120	73
12^d^	—	—	110	75
**13** d	—	—	**100**	**85**
14^d^	—	—	90	60
15^d^^,^^e^	—	—	100	86

a Reactions conditions: 1a (0.6 mmol), 2a (0.5 mmol), catalyst (10 mol% to 2a), solvent (1 mL), sealed tube, 10 h.

b Isolated yield of pure product based on 2a.

c Cs_2_CO_3_ as base was added to the reaction.

d 1a (1.5 mmol), 2a (0.5 mmol), sealed tube, 10 h.

e Reaction time was 15 h.

**Fig. 1:**
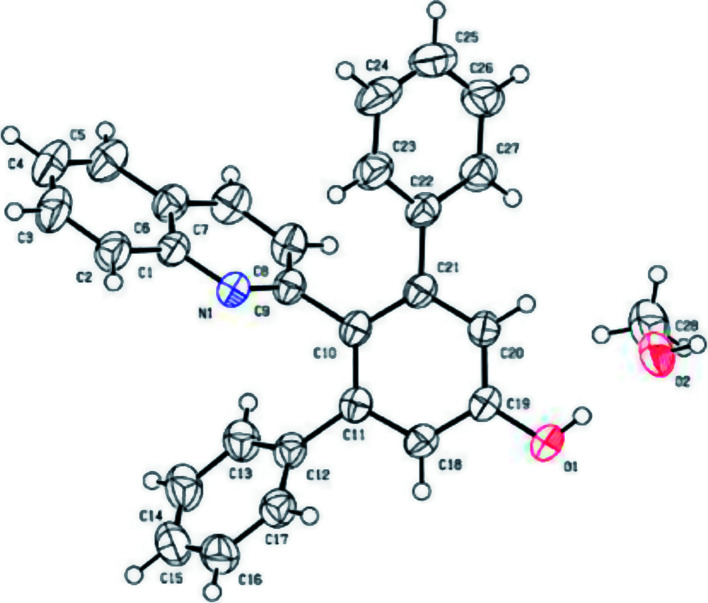
X-ray crystal structure of 3a (CCDC 1534893†).

With the optimized conditions in hand, a series of diynones and 2-methylquinolines were subjected to the reaction to investigate the scope and the results were shown in Table 2. The 2-methylquinoline ring has been substituted with electron-rich or electron-deficient groups R^1^ whereas R^2^, R^3^ in the diynones included alkyl and aryl moieties. All reactions proceeded smoothly to afford the corresponding 4-(quinolin-2-yl)phenols/4-(pyridin-2-yl)phenols in moderate to high yields (55–89%). Desired products 3b–3f were obtained in moderate to good yields (55–77%) with an electron-rich group (–EtO) or electron-deficient substituent (–F, –Cl, –Br) at C-5, C-6, or C-7 of 2-methylquinolines. The reaction of 6,7-dichloro-2-methylquinoline and diynones provided the corresponding product 3g in 73% yield with the PhCl as solvent. Symmetric diynones bearing electron-donating substituents such as Me, MeO, and Et or electron-withdrawing groups such as F and Cl on the benzene ring were found to be good substrates for this reaction and provided the desired products (3l–3q) in moderate to high yields, which showed that the position of the substituents on the benzene ring did not affect the transformation significantly. In addition, the diynones reacted with 2-methylquinoline which has an electron-deficient substituent at C-6 or C-7, furnishing the corresponding 4-(quinolin-2-yl)-phenols products in good yields (3h, 3i). It was found that the reaction of the 3-methylbenzo[*f*]quinoline and 2,6-dimethylpyridine also proceeded smoothly and afforded the desired product 3j and 3k in 82% and 68% yields, respectively. Unfortunately, the reaction of 2-methylpyridine and 1,5-diphenylpenta-1,4-diyn-3-one (2a) under the standard conditions only give a trace amount of the desired product. Then, we investigated the reaction with heterocycle substituted diynones, and found the thiophene substrates furnishing the desired product in higher yield (3v). Subsequently, other asymmetrical diynones which have two different substituents were also tested for the present reaction and the corresponding products were isolated in good to excellent yields (3r–3u, 3x–3z). We found that strained cyclopropyl was tolerated for this transformation and provided the corresponding products 3w and 3y in moderate yields.

**Table 2:** Synthesis of 4-(quinolin-2-yl)phenol derivatives^a^^,^^b^^,^^c^

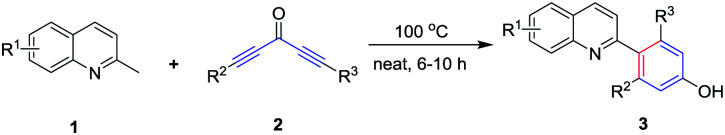
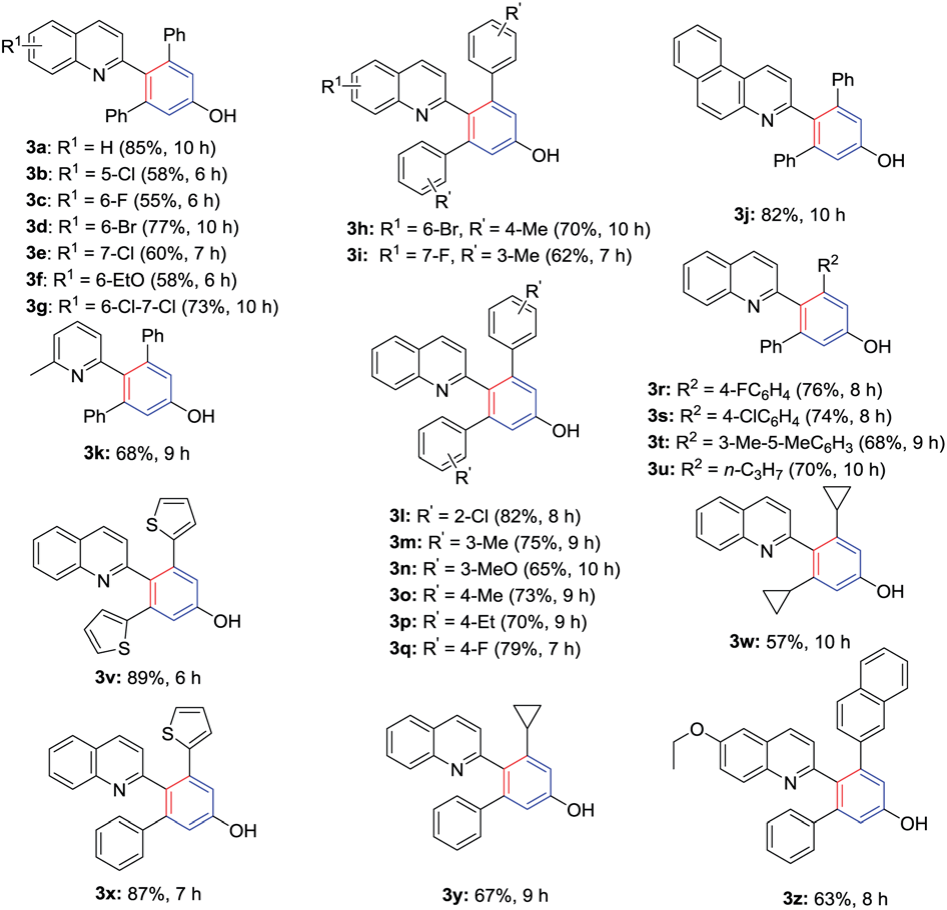

a Yield of the isolated product, calculated from 2.

b The reaction completed under solvent-free (see ESI).

c 3d, 3g, 3h, 3j completed in PhCl (see ESI).

To support the proposed reaction pathway, additional control experiments were carried out and the results were presented in Scheme 2. It was observed that the presence of 2 equiv. of 1,1-diphenylethylene or BHT (2,6-di-*tert*-butyl-4-methylphenol) didn't suppress the synthesis of 3a. These results suggested that a radical mechanism wasn't likely involved.

**Scheme 2:**
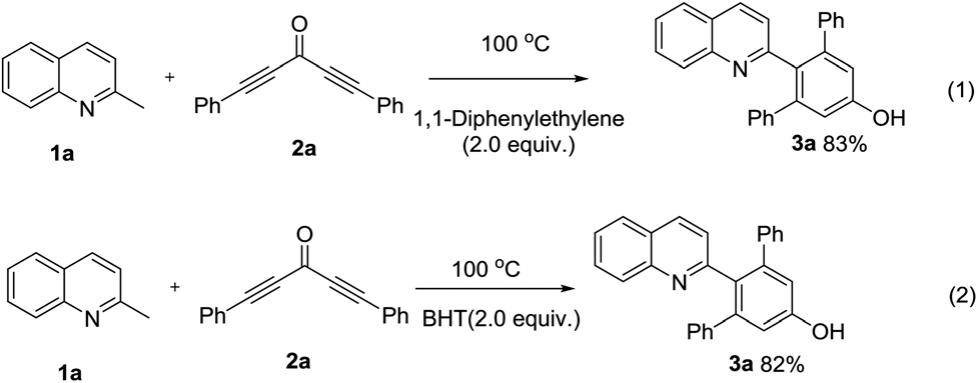
Control experiments.

A possible mechanism is proposed in Scheme 3. At first, the enamine intermediate A was formed from 1a*via* tautomerization,^22^ followed by, Michael addition to diynones, giving the intermediate B. And then, the enamine intermediate C was generated from B*via* the requisite disruption of aromaticity. Subsequently, intermediate C was transformed into intermediate D by intramolecular annulation reaction and the intermediate D was rapidly aromatized to form the stable product 3a.

**Scheme 3:**
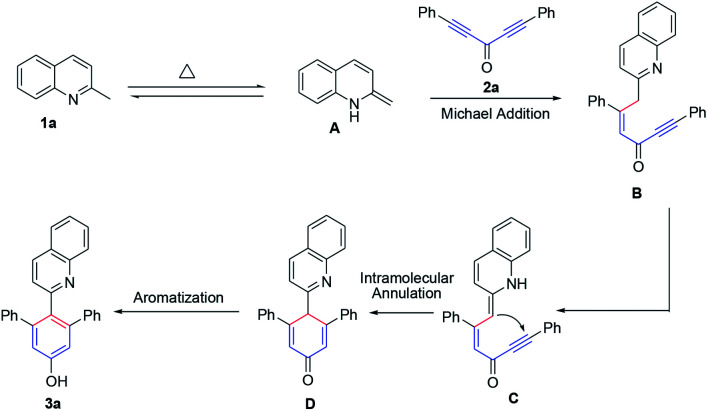
Plausible mechanism.

In summary, we have developed a rapid, simple, efficient, catalyst-free, and solvent-free reaction to access 4-(quinolin-2-yl)-phenols through a [5 + 1] annulation directly from 2-methylquinolines and diynones. The synthetic process was atom-economic, applicable to wide range of substrates, and has functional group tolerance, and these features would render this method attractive for academic and industrial use.

## Conflicts of interest

There are no conflicts to declare.

## Supplementary Material

RA-008-C7RA12716B-s001

RA-008-C7RA12716B-s002

## References

[cit1] Hitora Y., Takada K., Ise Y., Okada S., Matsunaga S. (2016). J. Nat. Prod..

[cit2] Gutekunst W. R., Gianatassio R., Baran P. S. (2012). Angew. Chem., Int. Ed..

[cit3] Friesen R. W., Brideau C., Chan C. C., Charleson S., Deschênes D., Dubé D., Ethier D., Fortin R., Gauthier J. Y., Girard Y., Gordon R., Greig G. M., Riendeau D., Savoie C., Wang Z., Wong E., Visco D., Xu L. J., Young R. N. (1998). Bioorganic Med. Chem. Lett..

[cit4] Quirk J., Thornton M., Kirkpatrick P. (2003). Nature.

[cit5] Capdeville R., Buchdunger E., Zimmermann J., Matter A. (2002). Nature.

[cit6] Chelucci G., Orru G., Pinna G. A. (2003). Tetrahedron.

[cit7] Kaila N., Janz K., DeBernardo S., Bedard P. W., Camphausen R. T., Tam S., Tsao D. H. H., Keith Jr J. C., Nickerson-Nutter C., Shilling A., Young-Sciame R., Wang Q. (2007). J. Med. Chem..

[cit8] Iglesias M. J., Prieto A., Nicasio M. C. (2012). Org. Lett..

[cit9] Arumugam V., Kaminsky W., Nallasamy D. (2015). RSC Adv..

[cit10] Haas D., Hammann J. M., Lutter F. H., Knochel P. (2016). Angew. Chem., Int. Ed..

[cit11] Lützen A., Hapke M. (2002). Eur. J. Org. Chem..

[cit12] Kong L.-H., Yu S.-J., Zhou X.-K., Li X.-W. (2016). Org. Lett..

[cit13] Li S.-M., Huang J., Chen G.-J., Han F.-S. (2011). Chem. Commun..

[cit14] Bergman A. M., Bergman R. G., Ellman J. A. (2010). J. Org. Chem..

[cit15] Deb A., Manna S., Maji A., Dutta U., Maiti D. (2013). Eur. J. Org. Chem..

[cit16] Hyodo I., Tobisu M., Chatani N. (2012). Chem.–Asian J..

[cit17] Mierde H. V., Voort P. V. D., Vos D. D., Verpoort F. (2008). Eur. J. Org. Chem..

[cit18] Mastalir M., Glatz M., Pittenauer E., Allmaier G., Kirchner K. (2016). J. Am. Chem. Soc..

[cit19] Balaram V. (2016). TrAC, Trends Anal. Chem..

[cit20] Wu F.-S., Zhao H.-Y., Xu Y.-L., Hu K., Pan Y.-M., Ma X.-L. (2017). J. Org. Chem..

[cit21] CCDC 1534893 (3a) contains the supplementary crystallographic data for this paper.†

[cit22] Xu L.-B., Shao Z.-Z., Wang L., Zhao H.-L., Xiao J. (2014). Tetrahedron Lett..

