# Supplementary material for: Catalyst- and solvent-free approach to 2-arylated quinolines via [5 + 1] annulation of 2-methylquinolines with diynones
Source: RSC Adv. 2018 Jan 25;8(9):4584–7. doi: 10.1039/c7ra12716b (PMC9077778; doi:10.1039/c7ra12716b)
Supplement: RA-008-C7RA12716B-s001 [file RA-008-C7RA12716B-s001.pdf]

Electronic Supplementary Material (ESI) for RSC Advances.
This journal is © The Royal Society of Chemistry 2018

Catalyst- and solvent-free approach to 2-arylated quinolines via [5+1] annulation of 2-methylquinolines with diynones

(Electronic Supplementary Information)

Hai-Yuan Zhao[a,b†], Fu-Song Wu[b†], Li Yang[c], Ying Liang[a*], Xiao-Lin Cao[b], Heng-Shan Wang[b] and Ying-Ming Pan[b*]

[a] School of Life and Environmental Sciences, Guilin University of Electronic Technology, Guilin, 541004, (China)

[b] State Key Laboratory for Chemistry and Molecular Engineering of Medicinal Resources, School of Chemistry and Pharmaceutical Sciences of Guangxi Normal University, Guilin 541004 (China)

[C] Guangxi Key Laboratory of Special Non-wood Forest Cultivation and Utilization, Guangxi Zhuang Autonomous Region Forestry Research Institute, Nanning, 530002 (China)

†These authors contributed equally to this work.

Table of Contents

General methods and materials......S2

Procedure of synthesis of starting materials 1......S2

Synthesis of 4-(quinolin-2-yl)phenol Derivatives 3a-3c, 3e-3f, 3i, 3k-3z......S3

Synthesis of 4-(quinolin-2-yl)phenol Derivatives 3d, 3g, 3h, and 3j......S3

References......S3

Spectral data of all compounds......S4

Copies of ^{1}H NMR and ^{13}C NMR spectra of all compounds......S17

General methods and materials. Proton nuclear magnetic resonance spectra (**^{1}H NMR**) and carbon nuclear magnetic resonance spectra (**^{13}C NMR**) were recorded at 400 MHz and 100 MHz or 500 MHz and 125 MHz, respectively, using DMSO-d_6 as reference standard (δ 2.50 ppm) for **^{1}H NMR** and (δ 40.00 ppm) for **^{13}C NMR** or $CDCl_3$ as reference standard (δ 7.26 ppm) for ^{1}H NMR and (δ 77.00 ppm) for ^{13}C NMR. **HRMS** (ion trap) were recorded using ESI. Melting points were uncorrected. Precoated silica gel plates F-254 were used for analytical thin-layer chromatography. Column chromatography was performed on silica gel (300-400 mesh). Starting materials were readily prepared according to literature procedures. Unless otherwise noted, all reagents were obtained commercially and used without further purification.

Procedure of synthesis of starting materials 1[1]:

1) To a stirred solution of acetylene (**A**; 10.2 mmol)) in dry THF (30 mL) under argon was added *n*-BuLi (4.5 mL, 11.2 mmol, 2.5 M solution in hexane) via a syringe at 0 °C. The mixture was allowed to stir for 30 min at 0 °C. Then methyl formate (0.28 mL, 4.52 mmol) was added. The mixture was allowed to stir for an additional 30 min at 0 °C, and then for 2 h at room temperature. The mixture was poured into an aqueous saturated solution of NH_4Cl, and the product was extracted with ethyl acetate. The combined organic layers were washed with a saturated aqueous sodium chloride solution, dried over $MgSO_4$, and filtered, and the solvents were removed under reduced pressure. The residue was purified by flash column chromatography (silica gel, PE/EA = 10/1) to give **B**.

Procedure A: MnO_2 (15 equiv.) was added to a solution of **B** (5 mmol) in CH_2Cl_2 (15 mL) at room temperature. The resulting mixture was stirred overnight. Then the solid was filtered, and the solvents were removed under reduced pressure. The residue was purified by flash column chromatography (silica gel, PE/EA = 50/1) to give **2**.

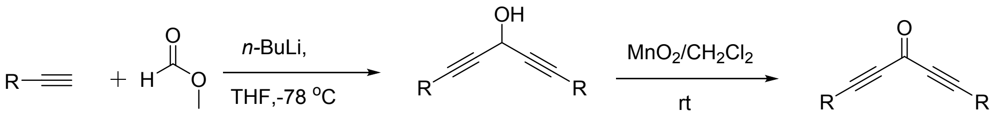

A B 2

2) To a stirred solution of acetylene (**A**, 8 mmol) in dry THF (30 mL) under argon was added *n*-BuLi (3.36 mL, 8.4 mmol, 2.5 M solution in hexane) via a syringe at 0 °C. The mixture was allowed to stir for 30 min at 0 °C. Then 3-phenylpropiolaldehyde (**C**, 8 mmol) was added. The mixture was allowed to stir for an additional 30 min at 0 °C, and then for 2 h at room temperature. It was then poured into aqueous saturated NH_4Cl solution and extracted with ethyl acetate. The combined organic layers were washed with a saturated aqueous sodium chloride solution, dried over $MgSO_4$, and filtered, and the solvents were removed under reduced pressure. The residue was purified by flash column chromatography (silica gel, PE/EA = 10/1) to give **D**.

D used the same procedure as general procedure A to give **2** (silica gel, PE/EA = 50/1).

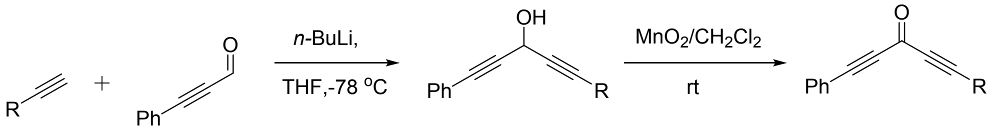

A C D 2

Synthesis of 4-(quinolin-2-yl)phenol Derivatives 3a-3c, 3e-3f, 3i, 3k-3z.

The reaction mixture of **1** (1.5 mmol), **2** (0.5 mmol), were stirred at 100 °C for 6-10 h in a sealed tube, and monitored periodically by TLC. After the resulting solution cooled to room temperature, the target product **3** was purified and raw material **1** was recycled by column chromatography on silica gel using a mixture of ethyl acetate and petroleum ether.

Synthesis of 4-(quinolin-2-yl)phenol Derivatives 3d, 3g, 3h, and 3j.

1 (0.6 mmol) and **2** (0.5 mmol) were dissolved in PhCl (1 mL), and stirred at 100 °C for 10 h in a sealed tube. The reaction monitored periodically by TLC. After the resulting solution cooled to room temperature, the target product **3** was purified by column chromatography on silica gel using a mixture of ethyl acetate and petroleum ether.

References

[1] Q.-H. Teng, Y.-L. Xu, Y. Liang, H.-S. Wang, Y.-C. Wang, Y.-M. Pan, *Adv. Synth. Catal.* **2016**, 358, 1897.

Spectral data of all compounds

2'-(quinolin-2-yl)-[1,1':3',1''-terphenyl]-5'-ol **(3a)**

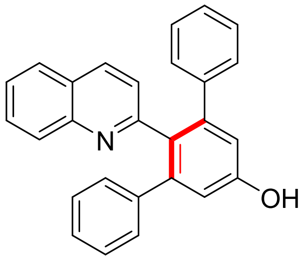

3a

The reaction of 2-methylquinoline (**1a**) (214.8 mg, 1.5 mmol), and 1,5-diphenylpenta-1,4-diyn-3-one (**2a**) (115.1 mg, 0.5 mmol) at 100 °C for 10 h, isolated by column chromatography (silica gel, petroleum ether/AcOEt = 10/1), affords 158.7 mg (85%) of **3a** as light yellow solid. **m.p.** 205-207 °C.

^{1}H NMR (400 MHz, DMSO-d_6): δ 10.05 (s, 1H), 7.86 (d, *J* = 8.4 Hz, 1H), 7.73 (d, *J* = 8.0 Hz, 1H), 7.66 (d, *J* = 8.4 Hz, 1H), 7.55–7.53 (m, 1H), 7.42–7.40 (m, 1H), 7.05 (s, 10H), 6.97 (d, *J* = 8.4 Hz, 1H), 6.88 (s, 2H) ppm.

^{13}C NMR (100 MHz, DMSO-d_6): δ 159.8, 157.4, 147.4, 143.6, 141.9, 135.0, 130.7, 129.7, 129.2, 128.2, 128.1, 127.0, 126.7, 126.2, 125.7, 116.6.

HRMS (*m/z*) (ESI): calcd for $C_{27}H_{20}NO$ 374.1539 [M+H^{+}]; found 374.1535.

2'-(5-chloroquinolin-2-yl)-[1,1':3',1''-terphenyl]-5'-ol **(3b)**

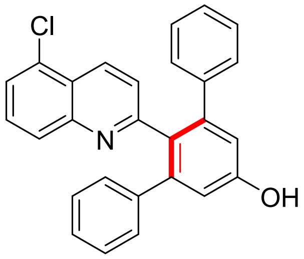

3b

The reaction of 5-chloro-2-methylquinoline (**1b**) (266.4 mg, 1.5 mmol), and 1,5-diphenylpenta-1,4-diyn-3-one (**2a**) (115.1 mg, 0.5 mmol) at 100 °C for 6 h, isolated by column chromatography (silica gel, petroleum ether/AcOEt = 10/1), affords 118.3 mg (58%) of **3b** as light yellow solid. **m.p.** 221-223 °C.

^{1}H NMR (400 MHz, DMSO-d_6): δ 10.01 (s, 1H), 8.09 (d, J = 8.7 Hz, 1H), 7.68–7.64 (m, 3H), 7.14 (d, J = 8.7 Hz, 1H), 7.11–7.02 (m, 10H), 6.87 (s, 2H) ppm.

^{13}C NMR (100 MHz, DMSO-d_6): δ 160.8, 157.6, 148.0, 143.5, 141.6, 131.0, 130.3, 129.9, 129.8, 129.6, 128.8, 128.2, 127.0, 126.9, 126.8, 123.8, 116.5 ppm.

HRMS (m/z) (ESI): calcd for $C_{27}H_{18}ClNO$ 408.1150 [M+H$^+$]; found 408.1146.

2'-(6-fluoroquinolin-2-yl)-[1,1':3',1''-terphenyl]-5'-ol **(3c)**

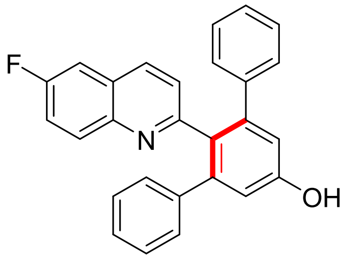

3c

The reaction of 6-fluoro-2-methylquinoline (**1c**) (241.8 mg, 1.5 mmol), and 1,5-diphenylpenta-1,4-diyn-3-one (**2a**) (115.1 mg, 0.5 mmol) at 100 °C for 6 h, isolated by column chromatography (silica gel, petroleum ether/AcOEt = 10/1), affords 107.7 mg (55%) of **3c** as light yellow solid. **m.p.** 238-240 °C.

^{1}H NMR (400 MHz, DMSO-d_6): δ 9.97 (s, 1H), 7.88 (d, J = 8.5 Hz, 1H), 7.69–7.66 (m, 1H), 7.54–7.50 (m, 1H), 7.46–7.44 (m, 1H), 7.09–7.03 (m, 10H), 7.03–6.99 (m, 1H), 6.88 (s, 2H) ppm.

^{13}C NMR (100 MHz, DMSO-d_6): δ 161.2, 159.2, 158.7, 157.4, 144.5, 143.5, 141.7, 134.4, 131.8, 130.2, 129.6, 128.1, 126.9, 126.7, 126.3, 119.5, 116.5, 111.1 ppm.

HRMS (m/z) (ESI): calcd for $C_{27}H_{19}FNO$ 392.1445 [M+H$^+$]; found 392.1440.

2'-(6-bromoquinolin-2-yl)-[1,1':3',1''-terphenyl]-5'-ol **(3d)**

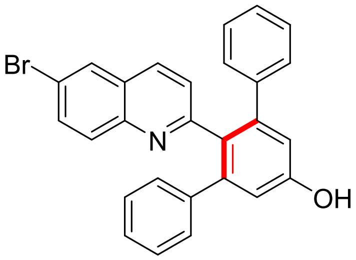

3d

The reaction of 6-bromo-2-methylquinoline (**1d**) (133.3 mg, 0.6 mmol), and 1,5-diphenylpenta-1,4-diyn-3-one (**2a**) (115.1 mg, 0.5 mmol) in PhCl (1 mL) at 100 °C for 10 h, isolated by column chromatography (silica gel, petroleum ether/AcOEt = 10/1), affords 174.2 mg (77%) of **3d** as light yellow solid. **m.p.** 239-241 °C.

^{1}H NMR (400 MHz, DMSO-d_6): δ 9.97 (s, 1H), 8.04 (d, J = 2.2 Hz, 1H), 7.87 (d, J = 8.5 Hz, 1H), 7.67–7.62 (m, 1H), 7.56 (d, J = 9.0 Hz, 1H), 7.07–7.00 (m, 11H), 6.86 (s, 2H) ppm.

^{13}C NMR (100 MHz, DMSO-d_6): δ 160.4, 157.5, 145.9, 143.4, 141.6, 134.1, 132.6, 131.3, 130.2, 130.0, 129.6, 128.1, 127.4, 126.9, 126.5, 119.5, 116.5 ppm.

HRMS (*m/z*) (ESI): calcd for $C_{27}H_{19}BrNO$ 452.0645, 454.0624 [M+H$^+$]; found 452.0643, 454.0621.

2'-(7-chloroquinolin-2-yl)-[1,1':3',1''-terphenyl]-5'-ol **(3e)**

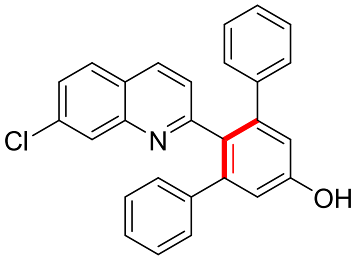

3e

The reaction of 7-chloro-2-methylquinoline (**1e**) (266.4 mg, 1.5 mmol), and 1,5-diphenylpenta-1,4-diyn-3-one (**2a**) (115.1 mg, 0.5 mmol) at 100 °C for 7 h, isolated by column chromatography (silica gel, petroleum ether/AcOEt = 10/1), affords 122.4 mg (60%) oef **3c** as light yellow solid. **m.p.** 125-127 °C.

^{1}H NMR (400 MHz, DMSO-d_6): δ 9.96 (s, 1H), 7.94 (d, J = 8.4 Hz, 1H), 7.92 (d, J = 8.5 Hz, 1H), 7.81 (d, J = 8.7 Hz, 1H), 7.66 (d, J = 2.1 Hz, 1H), 7.48–7.44 (m, 1H), 7.10–7.02 (m, 9H), 7.01 (d, J = 8.5 Hz, 1H), 6.85 (s, 2H) ppm.

^{13}C NMR (100 MHz, DMSO-d_6): δ 161.1, 157.5, 147.6, 143.4, 141.6, 134.9, 134.1, 130.1, 130.1, 129.6, 128.1, 127.6, 127.2, 126.9, 126.1, 124.7, 116.5 ppm.

HRMS (*m/z*) (ESI): calcd for $C_{27}H_{18}ClNO$ 408.1150 [M+H$^+$]; found 408.1145.

2'-(6-ethoxyquinolin-2-yl)-[1,1':3',1''-terphenyl]-5'-ol **(3f)**

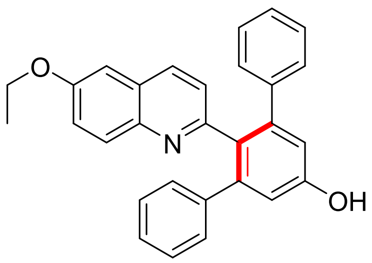

3f

The reaction of 6-ethoxy-2-methyl-quinolin (**1f**) (280.86 mg, 1.5 mmol), and 1,5-diphenylpenta-1,4-diyn-3-one (**2a**) (115.1 mg, 0.5 mmol) at 100 °C for 6 h, isolated by column chromatography (silica gel, petroleum ether/AcOEt = 10/1), affords 121.1 mg (58%) of **3f** as light yellow solid. **m.p.** 155-157 °C.

^{1}H NMR (400 MHz, DMSO-d_6): δ 9.90 (s, 1H), 7.75 (d, J = 8.5 Hz, 1H), 7.56 (d, J = 9.2 Hz, 1H), 7.20–7.12 (m, 1H), 7.11 (d, J = 2.7 Hz, 1H), 7.07 (d, J = 5.9 Hz, 10H), 6.91 (d, J = 8.4 Hz, 1H), 6.88 (s, 2H), 4.06 (q, J = 6.8 Hz, 2H), 1.34 (t, J = 6.8 Hz, 3H) ppm.

^{13}C NMR (100 MHz, DMSO-d_6): δ 157.2, 156.9, 156.7, 143.3, 143.2, 141.9, 133.7, 130.6, 130.5, 129.6, 128.0, 127.1, 126.7, 125.7, 122.0, 116.4, 106.4, 63.8, 15.0 ppm.

HRMS (*m/z*) (ESI): calcd for $C_{29}H_{24}NO_2$ 418.1802 [M+H$^+$]; found 418.1803.

2'-(6,7-dichloroquinolin-2-yl)-[1,1':3',1''-terphenyl]-5'-ol **(3g)**

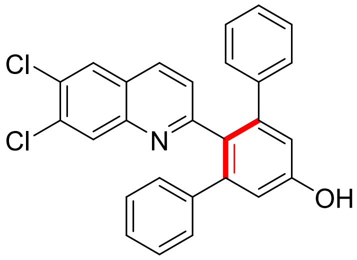

3g

The reaction of 6,7-dichloro-2-methylquinoline (**1g**) (127.2 mg, 0.6 mmol), and 1,5-diphenylpenta-1,4-diyn-3-one (**2a**) (115.1 mg, 0.5 mmol) in PhCl (1 mL) at 100 °C for 10 h, isolated by column chromatography (silica gel, petroleum ether/AcOEt = 10/1), affords 161.4 mg (73%) of **3g** as light yellow solid. **m.p.** 186-187 °C.

^{1}H NMR (400 MHz, DMSO-d_6): δ 10.02 (s, 1H), 8.15 (s, 1H), 7.92 (d, J = 8.5 Hz, 1H), 7.88 (s, 1H), 7.11–7.05 (m, 7H), 7.03–6.97 (m, 4H), 6.85 (s, 2H) ppm.

^{13}C NMR (100 MHz, DMSO-d_6): δ 161.7, 157.6, 146.0, 143.4, 141.5, 134.2, 132.3, 130.0, 129.8, 129.6, 129.3, 129.2, 128.2, 127.0, 125.6, 116.5 ppm.

HRMS (*m/z*) (ESI): calcd for $C_{27}H_{18}Cl_2NO$ 442.0760 [M+H$^+$]; found 442.0775.

2'-(6-bromoquinolin-2-yl)-4,4''-dimethyl-[1,1':3',1''-terphenyl]-5'-ol **(3h)**

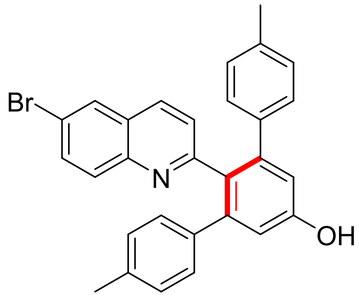

3h

The reaction of 6-bromo-2-methylquinoline (**1d**) (133.3 mg, 0.6 mmol), and 1,5-di-p-tolylpenta-1,4-diyn-3-one (**2b**) (129.1 mg, 0.5 mmol) in PhCl (1 mL) at 100 °C for 10 h, isolated by column chromatography (silica gel, petroleum ether/AcOEt = 10/1), affords 167.7 mg (70%) of **3h** as light yellow solid. **m.p.** 136-139 °C.

^{1}H NMR (400 MHz, DMSO-d_6): δ 9.88 (s, 1H), 8.05 (d, J = 2.1 Hz, 1H), 7.89 (d, J = 8.5 Hz, 1H), 7.69 –7.62 (m, 1H), 7.61 (d, J = 9.0 Hz, 1H), 7.03 (d, J = 8.5 Hz, 1H), 6.89–6.83 (m, 8H), 6.80 (s, 2H), 2.11 (s, 6H) ppm.

^{13}C NMR (100 MHz, DMSO-d_6): δ 160.8, 157.4, 145.8, 143.2, 138.7, 135.9, 134.1, 132.6, 131.4, 130.1, 130.0, 129.5, 128.7, 127.5, 126.6, 119.4, 116.3, 21.0 ppm.

HRMS (*m/z*) (ESI): calcd for $C_{29}H_{23}BrNO$ 480.0958 482.0937 [M+H$^+$]; found 480.0959 482.0934.

2'-(7-fluoroquinolin-2-yl)-3,3''-dimethyl-[1,1':3',1''-terphenyl]-5'-ol **(3i)**

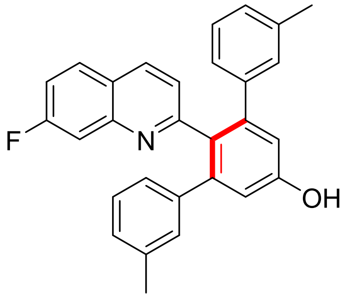

3i

The reaction of 7-Fluoro-2-methylpyridine (**1h**) (241.8 mg, 1.5 mmol), and 1,5-di-m-tolylpenta-1,4-diyn-3-one (**2c**) (129.1 mg, 0.5 mmol) at 100 °C for 7 h, isolated by column chromatography (silica gel, petroleum ether/AcOEt = 10/1), affords 130.0 mg (62%) of **3i** as light yellow solid. **m.p.** 171-173 °C.

^{1}H NMR (400 MHz, DMSO-d_6): δ 9.88 (s, 1H), 7.94 (d, J = 8.5 Hz, 1H), 7.86–7.80 (m, 1H), 7.40–7.32 (m, 3H), 6.97–6.93 (m, 3H), 6.91–6.86 (m, 2H), 6.88–6.82 (m, 3H), 6.78 (d, J = 7.5 Hz, 2H), 2.06 (s, 6H) ppm.

^{13}C NMR (100 MHz, DMSO-d_6): δ 163.8, 161.4, 161.2, 157.4, 148.0, 143.3, 141.5, 137.1, 134.9, 130.6, 130.5, 130.2, 127.8, 127.5, 126.8, 125.1, 123.3, 116.7, 116.3, 112.4, 21.3 ppm.

HRMS (*m/z*) (ESI): calcd for $C_{29}H_{23}FNO$ 420.1758 [M+H$^+$]; found 420.1748.

2'-(benzo[f]quinolin-3-yl)-[1,1':3',1''-terphenyl]-5'-ol **(3j)**

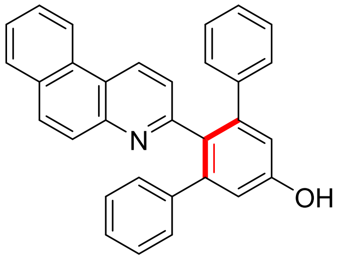

3j

The reaction of 3-methylbenzo[f]quinoline (**1i**) (116.0 mg, 0.6 mmol), and 1,5-diphenylpenta-1,4-diyn-3-one (**2a**) (115.1 mg, 0.5 mmol) in PhCl (1 mL) at 100 °C for 10 h, isolated by column chromatography (silica gel, petroleum ether/AcOEt = 10/1), affords 173.6 mg (82%) of **3j** as light yellow solid. **m.p.** 153-155 °C.

^{1}H NMR (400 MHz, DMSO-d_6): δ 9.93 (s, 1H), 8.78 (d, J = 8.6 Hz, 1H), 8.65 (d, J = 7.8 Hz, 1H), 7.97–7.90 (m, 2H), 7.67–7.61 (m, 2H), 7.57 (d, J = 9.1 Hz, 1H), 7.14 (d, J = 8.5 Hz, 1H), 7.10–7.01 (m, 10H), 6.89 (s, 2H) ppm.

^{13}C NMR (100 MHz, DMSO-d_6): δ 159.2, 157.3, 146.9, 143.5, 141.8, 131.5, 130.6, 130.3, 130.3, 129.6, 129.0, 128.3, 128.0, 127.6, 127.6, 126.8, 125.8, 123.5, 122.8, 116.5 ppm.

HRMS (*m/z*) (ESI): calcd for $C_{31}H_{22}NO$ 424.1696 [M+H$^+$]; found 424.1690.

2'-(6-methylpyridin-2-yl)-[1,1':3',1''-terphenyl]-5'-ol **(3k)**

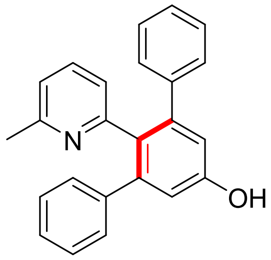

3k

The reaction of 2,6-dimethyl-pyridin (**1j**) (160.7 mg, 1.5 mmol), and 1,5-diphenylpenta-1,4-diyn-3-one (**2a**) (115.1 mg, 0.5 mmol) at 100 °C for 9 h, isolated by column chromatography (silica gel, petroleum ether/AcOEt = 10/1), affords 114.7 mg (68%) of **3k** as light yellow solid. **m.p.** 153-155 °C.

^{1}H NMR (400 MHz, DMSO-d_6) δ 9.91 (s, 1H), 7.20 (d, J = 7.7 Hz, 1H), 7.16–7.11 (m, 6H), 7.04–7.00 (m, 4H), 6.79 (d, J = 5.9 Hz, 3H), 6.56 (d, J = 7.6 Hz, 1H), 2.13 (s, 3H) ppm.

^{13}C NMR (100 MHz, DMSO-d_6): δ 158.1, 157.1, 156.5, 143.2, 142.0, 135.6, 130.5, 129.6, 127.9, 126.7, 124.2, 120.3, 116.3, 24.3 ppm.

HRMS (*m/z*) (ESI): calcd for $C_{24}H_{19}NO$ 338.1539 [M+H$^+$]; found 338.1540.

2,2''-dichloro-2'-(quinolin-2-yl)-[1,1':3',1''-terphenyl]-5'-ol **(3l)**

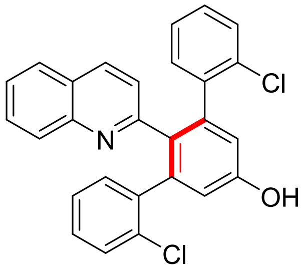

3l

The reaction of 2-methylquinoline (**1a**) (214.8 mg, 1.5 mmol), and 1,5-bis(2-chlorophenyl)penta-1,4-diyn-3-one (**2d**) (149.6 mg, 0.5 mmol) at 100 ^{o}C for 8 h, isolated by column chromatography (silica gel, petroleum ether/AcOEt = 10/1), affords 181.4 mg (82%) of **3l** as light yellow solid. **m.p.** 235-237 ^{o}C.

^{1}H NMR (400 MHz, DMSO-d_6): δ 10.10 (s, 1H), 7.86 (d, J = 8.5 Hz, 1H), 7.70 (d, J = 8.1 Hz, 1H), 7.55–7.49 (m, 2H), 7.40–7.32 (m, 1H), 7.17–7.10 (m, 4H), 6.98–6.91 (m, 7H) ppm.

^{13}C NMR (100 MHz, DMSO-d_6): δ 160.5, 158.4, 158.1, 157.2, 147.2, 137.3, 134.7, 132.3, 132.0, 129.7, 129.6, 129.5, 129.2, 129.1, 129.0, 127.9, 126.5, 126.0, 124.3, 124.1, 117.7, 115.6, 115.4 ppm.

HRMS (m/z) (ESI): calcd for $C_{27}H_{18}Cl_2NO$ 442.0760 [M+H$^+$]; found 442.0765.

3,3''-dimethyl-2'-(quinolin-2-yl)-[1,1':3',1''-terphenyl]-5'-ol **(3m)**

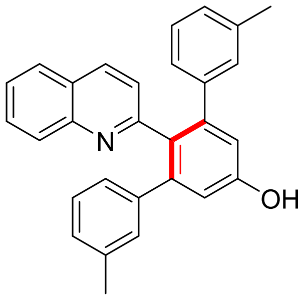

3m

The reaction of 2-methylquinoline (**1a**) (214.8 mg, 1.5 mmol), and 1,5-di-m-tolylpenta-1,4-diyn-3-one (**2c**) (129.1 mg, 0.5 mmol) at 100 ^{o}C for 9 h, isolated by column chromatography (silica gel, petroleum ether/AcOEt = 10/1), affords 150.6 mg (75%) of **3m** as light yellow solid. **m.p.**187-189 ^{o}C.

^{1}H NMR (500 MHz, DMSO-d_6): δ 9.90 (s, 1H), 7.89 (d, J = 8.5 Hz, 1H), 7.73–7.68 (m, 2H), 7.59–7.54 (m, 1H), 7.44–7.39 (m, 1H), 6.97 (d, J = 8.2 Hz, 3H), 6.92–6.73 (m, 8H), 2.04 (s, 6H) ppm.

^{13}C NMR (125 MHz, DMSO-d_6): δ 159.9, 157.3, 147.2, 143.4, 141.6, 137.0, 134.8, 130.5, 130.5, 129.6, 129.1, 128.0, 127.8, 127.5, 126.8, 126.5, 126.1, 125.6, 116.3, 21.3 ppm.

HRMS (m/z) (ESI): calcd for $C_{29}H_{24}NO$ 402.1852 [M+H$^+$]; found 402.1849.

3,3''-dimethoxy-2'-(quinolin-2-yl)-[1,1':3',1''-terphenyl]-5'-ol **(3n)**

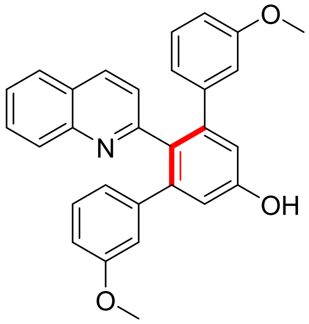

3n

The reaction of 2-methylquinoline (**1a**) (214.8 mg, 1.5 mmol), and 1,5-bis(3-methoxyphenyl)penta-1,4-diyn-3-one (**2e**) (145.2 mg, 0.5 mmol) at 100 °C for 10 h, isolated by column chromatography (silica gel, petroleum ether/AcOEt = 10/1), affords 140.8 mg (65%) of **3n** as light yellow solid. **m.p.** 103-104 °C.

^{1}H NMR (400 MHz, DMSO-d_6): δ 9.99 (s, 1H), 7.92 (d, J = 8.5 Hz, 1H), 7.75 (d, J = 8.5 Hz, 2H), 7.59–7.52 (m, 1H), 7.45–7.40 (m, 1H), 7.05 (d, J = 8.4 Hz, 1H), 7.00–6.92 (m, 2H), 6.93 (s, 2H), 6.71 (d, J = 7.6 Hz, 2H), 6.61–6.56 (m, 4H), 3.38 (s, 6H) ppm.

^{13}C NMR (100 MHz, DMSO-d_6): δ 160.0, 158.8, 157.4, 147.3, 143.2, 143.1, 135.1, 130.6, 129.7, 129.2, 129.1, 128.0, 126.6, 126.2, 125.5, 122.1, 116.5, 115.1, 113.1, 55.0 ppm.

HRMS (*m/z*) (ESI): calcd for $C_{29}H_{24}NO_3$ 434.1751 [M+H$^+$]; found 434.1751.

4,4''-dimethyl-2'-(quinolin-2-yl)-[1,1':3',1''-terphenyl]-5'-ol **(3o)**

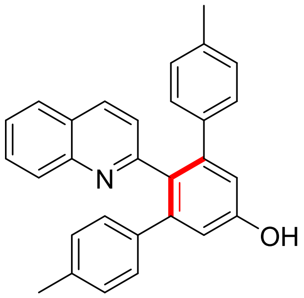

3o

The reaction of 2-methylquinoline (**1a**) (214.8 mg, 1.5 mmol), and 1,5-di-p-tolylpenta-1,4-diyn-3-one (**2b**) (129.1 mg, 0.5 mmol) at 100 °C for 9 h, isolated by column chromatography (silica gel, petroleum ether/AcOEt = 10/1), affords 146.6 mg (73%) of **3o** as light yellow solid. **m.p.** 178-180 °C.

^{1}H NMR (400 MHz, DMSO-d_6): δ 9.80 (s, 1H), 7.76 (d, J = 8.4 Hz, 1H), 7.61–7.56 (m, 2H), 7.50–7.43 (m, 1H), 7.36–7.25 (m, 1H), 6.89–6.72 (m, 5H), 6.81–6.68 (m, 6H), 1.96 (s, 6H) ppm.

^{13}C NMR (100 MHz, DMSO-d_6): δ 160.0, 157.3, 147.3, 143.3, 138.9, 135.8, 134.9, 130.5, 129.5, 129.2, 128.7, 128.0, 126.4, 126.2, 125.6, 116.4, 20.9 ppm.

HRMS (*m/z*) (ESI): calcd for $C_{29}H_{24}NO$ 402.1852 [M+H$^+$]; found 402.1856.

4,4''-diethyl-2'-(quinolin-2-yl)-[1,1':3',1''-terphenyl]-5'-ol **(3p)**

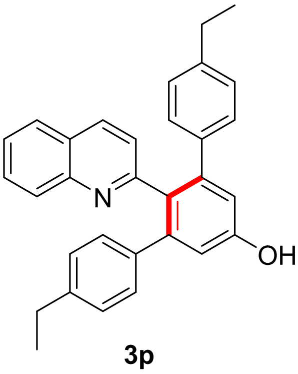

3p

The reaction of 2-methylquinoline (**1a**) (214.8 mg, 1.5 mmol), and 1,5-bis(4-ethylphenyl)penta-1,4-diyn-3-one (**2f**) (143.2 mg, 0.5 mmol) at 100 ^{o}C for 9 h, isolated by column chromatography (silica gel, petroleum ether/AcOEt = 10/1), affords 150.3 mg (70%) of **3p** as light yellow solid. **m.p.** 176-178 ^{o}C.

^{1}**H NMR** (400 MHz, DMSO-d_6): δ 9.87 (s, 1H), 7.87 (d, *J* = 8.5 Hz, 1H), 7.71–7.60 (m, 2H), 7.55–7.48 (m, 1H), 7.42–7.38 (m, 1H), 6.98–6.81 (m, 5H), 6.89–6.74 (m, 6H), 2.40 (q, *J* = 8.5 Hz, 4H), 1.01 (t, *J* = 8.4 Hz, 6H) ppm.

^{13}**C NMR** (100 MHz, DMSO-d_6): δ 159.9, 157.3, 147.3, 143.3, 142.1, 139.2, 134.8, 130.5, 129.5, 129.4, 129.2, 128.0, 127.4, 126.4, 126.1, 125.6, 116.4, 28.0, 15.6 ppm.

HRMS (*m/z*) (ESI): calcd for $C_{31}H_{28}NO$ 430.2165 [M+H^{+}]; found 430.2162.

4,4''-difluoro-2'-(quinolin-2-yl)-[1,1':3',1''-terphenyl]-5'-ol **(3q)**

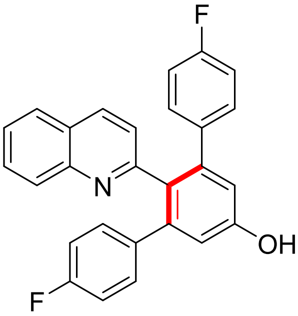

3q

The reaction of 2-methylquinoline (**1a**) (214.8 mg, 1.5 mmol), and 1,5-bis(4-fluorophenyl)penta-1,4-diyn-3-one (**2g**) (133.2 mg, 0.5 mmol) at 100 ^{o}C for 7 h, isolated by column chromatography (silica gel, petroleum ether/AcOEt = 10/1), affords 161.7 mg (79%) of **3q** as light yellow solid. **m.p.** 226-228 ^{o}C.

^{1}**H NMR** (400 MHz, DMSO-d_6): δ 10.13 (s, 1H), 7.91 (d, *J* = 8.5 Hz, 1H), 7.75 (d, *J* = 7.7 Hz, 1H), 7.67 (d, *J* = 8.4 Hz, 1H), 7.60–7.55 (m, 1H), 7.46–7.41 (m, 1H), 7.07 (m, 4H), 6.96 (d, *J* = 8.5 Hz, 1H), 6.86–6.69 (m, 6H) ppm.

^{13}**C NMR** (100 MHz, DMSO-d_6): δ 162.7, 160.3, 159.6, 157.6, 147.5, 142.6, 138.2, 135.4, 131.7, 131.7, 130.8, 130.0, 129.2, 128.3, 126.9, 126.3, 125.7, 116.8, 115.2, 115.0 ppm.

HRMS (*m/z*) (ESI): calcd for $C_{27}H_{18}F_2NO$ 410.1351 [M+H$^+$]; found 410.1349.

4-fluoro-2'-(quinolin-2-yl)-[1,1':3',1''-terphenyl]-5'-ol **(3r)**

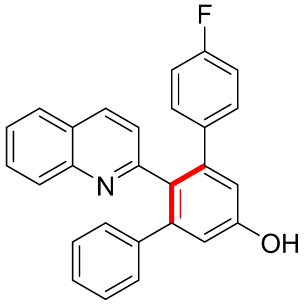

3r

The reaction of 2-methylquinoline (**1a**) (214.8 mg, 1.5 mmol), and 1-(4-fluorophenyl)-5-phenylpenta-1,4-diyn-3-one (**2h**) (124.1 mg, 0.5 mmol) at 100 ^{o}C for 8 h, isolated by column chromatography (silica gel, petroleum ether/AcOEt = 10/1), affords 148.8 mg (76%) of **3r** as light yellow solid. **m.p.** 189-191 ^{o}C.

^{1}**H NMR** (400 MHz, DMSO-d_6): δ 10.03 (s, 1H), 7.90 (d, *J* = 8.5 Hz, 1H), 7.74 (d, *J* = 8.0 Hz, 1H), 7.68 (d, *J* = 8.4 Hz, 1H), 7.59–7.54 (m, 1H), 7.45–7.41 (m, 1H), 7.07–6.86 (m, 7H), 6.99 (d, *J* = 8.4 Hz, 1H), 6.93–6.84 (m, 4H) ppm.

^{13}**C NMR** (100 MHz, DMSO-d_6): δ 162.5, 160.1, 159.6, 157.4, 147.4, 143.5, 142.5, 141.7, 138.2, 135.0, 131.5, 130.6, 129.6, 129.1, 128.1, 128.1, 126.9, 126.6, 126.1, 125.6, 116.6, 115.0, 114.8 ppm.

HRMS (*m/z*) (ESI): calcd for $C_{27}H_{19}FNO$ 392.1445 [M+H$^+$]; found 392.1442.

4-chloro-2'-(quinolin-2-yl)-[1,1':3',1''-terphenyl]-5'-ol **(3s)**

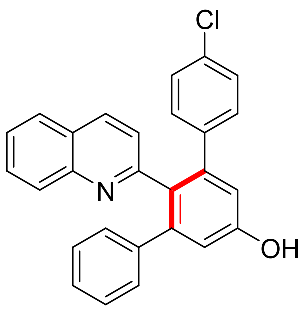

3s

The reaction of 2-methylquinoline (**1a**) (214.8 mg, 1.5 mmol), and 1-(4-chlorophenyl)-5-phenylpenta-1,4-diyn-3-one (**2i**) (132.4 mg, 0.5 mmol) at 100 °C for 8 h, isolated by column chromatography (silica gel, petroleum ether/AcOEt = 10/1), affords 150.9 mg (74%) of **3s** as light yellow solid. **m.p.** 232-235 °C.

^{1}H NMR (400 MHz, DMSO-d_6): δ 9.99 (s, 1H), 7.92 (d, J = 8.5 Hz, 1H), 7.76 (d, J = 7.9 Hz, 1H), 7.67 (d, J = 8.3 Hz, 1H), 7.60–7.56 (m, 1H), 7.45–7.39 (m, 1H), 7.12 (d, J = 8.5 Hz, 2H), 7.07 (d, J = 8.2 Hz, 7H), 6.99 (d, J = 8.4 Hz, 1H), 6.87 (s, 2H) ppm.

^{13}C NMR (100 MHz, DMSO-d_6): δ 159.3, 157.4, 147.3, 143.5, 142.1, 141.6, 140.7, 135.0, 131.7, 131.3, 130.4, 129.6, 129.6, 129.1, 128.1, 128.0, 126.9, 126.6, 126.1, 125.5, 116.8, 116.4 ppm.

HRMS (*m/z*) (ESI): calcd for $C_{27}H_{19}ClNO$ 408.1150 [M+H$^+$]; found 408.1147.

3,5-dimethyl-2'-(quinolin-2-yl)-[1,1':3',1''-terphenyl]-5'-ol **(3t)**

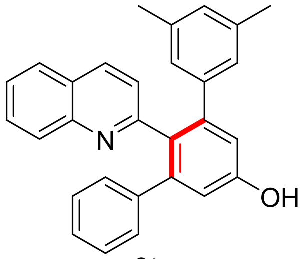

3t

The reaction of 2-methylquinoline (**1a**) (214.8 mg, 1.5 mmol), and 1-(3,5-dimethylphenyl)-5-phenylpenta-1,4-diyn-3-one (**2j**) (129.2 mg, 0.5 mmol) at 100 °C for 9 h, isolated by column chromatography (silica gel, petroleum ether/AcOEt = 10/1), affords 136.5 mg (68%) of **3t** as light yellow solid. **m.p.** 132-134 °C.

^{1}H NMR (400 MHz, DMSO-d_6): δ 9.90 (s, 1H), 7.87 (d, J = 8.5 Hz, 1H), 7.76–7.69 (m, 2H), 7.57–7.52 (m, 1H), 7.45–7.41 (m, 1H), 7.11–7.01 (m, 5H), 6.97 (d, J = 8.4 Hz, 1H), 6.88–6.82 (m, 2H), 6.70 (s, 2H), 6.64 (s, 1H), 1.95 (s, 6H) ppm.

^{13}C NMR (100 MHz, DMSO-d_6): δ 159.9, 157.2, 147.2, 143.5, 143.2, 141.8, 141.4, 136.7, 134.7, 130.5, 129.6, 129.5, 129.1, 128.2, 128.0, 127.9, 127.7, 126.8, 126.4, 126.1, 125.5, 116.3, 116.2, 21.1 ppm.

HRMS (*m/z*) (ESI): calcd for $C_{29}H_{23}NO$ 402.1852 [M+H$^+$]; found 402.1848.

5-propyl-6-(quinolin-2-yl)-[1,1'-biphenyl]-3-ol **(3u)**

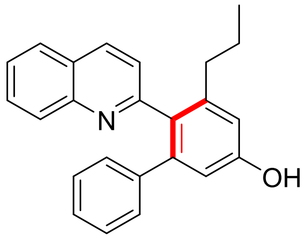

3u

The reaction of 2-methylquinoline (**1a**) (214.8 mg, 1.5 mmol), and 1-phenylocta-1,4-diyn-3-one (**2k**) (98.2 mg, 0.5 mmol) at 100 °C for 10 h, isolated by column chromatography (silica gel, petroleum ether/AcOEt = 10/1), affords 118.8 mg (70%) of **3u** as light yellow solid. **m.p.** 90-93 °C.

^{1}H NMR (400 MHz, DMSO-d_6): δ 9.68 (s, 1H), 7.98 (m, 2H), 7.84 (d, *J* = 8.1 Hz, 1H), 7.70 (m, 1H), 7.55–7.51 (m, 1H), 7.07–7.01 (m, 5H), 6.96 (d, *J* = 8.4 Hz, 1H), 6.79 (d, *J* = 2.3 Hz, 1H), 6.69 (d, *J* = 2.1 Hz, 1H), 2.38 (t, *J* = 7.2 Hz, 2H), 1.36–1.30 (m, 2H), 0.63 (t, *J* = 7.6 Hz, 3H) ppm.

^{13}C NMR (100 MHz, DMSO-d_6): δ 160.2, 157.3, 147.5, 143.0, 142.5, 141.9, 135.4, 131.0, 129.8, 129.6, 129.3, 128.2, 128.1, 126.9, 126.7, 126.4, 125.1, 115.8, 114.8, 35.7, 24.2, 14.4 ppm.

HRMS (*m/z*) (ESI): calcd for $C_{24}H_{22}NO$ 340.1696 [M+H$^+$]; found 340.1694.

4-(quinolin-2-yl)-3,5-di(thiophen-2-yl)phenol **(3v)**

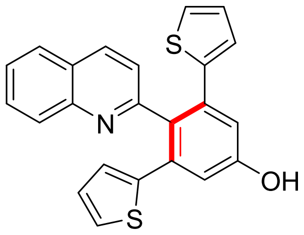

3v

The reaction of 2-methylquinoline (**1a**) (214.8 mg, 1.5 mmol), and 1,5-di(thiophen-2-yl)penta-1,4-diyn-3-one (**2l**) (121.2 mg, 0.5 mmol) at 100 °C for 6 h, isolated by column chromatography (silica gel, petroleum ether/AcOEt = 10/1), affords 171.5 mg (89%) of **3v** as light yellow solid. **m.p.** 184-186 °C.

^{1}H NMR (400 MHz, DMSO-d_6): δ 10.07 (s, 1H), 8.12 (d, *J* = 8.4 Hz, 1H), 7.95–7.89 (m, 2H), 7.72–7.65 (m, 1H), 7.57–7.51 (m, 1H), 7.26–7.15 (m, 2H), 7.18 (d, *J* = 8.4 Hz, 1H), 7.05 (s, 2H), 6.75–6.68 (m, 2H), 6.68–6.61 (m, 2H) ppm.

^{13}C NMR (100 MHz, DMSO-d_6): δ 159.3, 157.4, 147.5, 142.5, 136.2, 135.8, 130.2, 130.0, 129.5, 128.4, 127.5, 127.4, 127.2, 127.1, 125.2, 116.6 ppm.

HRMS (*m/z*) (ESI): calcd for $C_{23}H_{16}NOS_2$ 386.0673 [M+H$^+$]; found 386.0657.

3,5-dicyclopropyl-4-(quinolin-2-yl)phenol **(3w)**

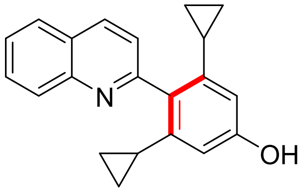

3w

The reaction of 2-methylquinoline (**1a**) (214.8 mg, 1.5 mmol), and 1,5-dicyclopropylpenta-1,4-diyn-3-one (**2m**) (79.1 mg, 0.5 mmol) at 100 °C for 10 h, isolated by column chromatography (silica gel, petroleum ether/AcOEt = 10/1), affords 85.9 mg (57%) of **3w** as White solid. **m.p.** 171-173 °C.

^{1}H NMR (400 MHz, DMSO-d_6): δ 9.34 (s, 1H), 8.37 (d, J = 8.4 Hz, 1H), 8.00–7.91 (m, 2H), 7.74–7.67 (m, 1H), 7.59–7.53 (m, 1H), 7.49 (d, J = 8.4 Hz, 1H), 6.19 (s, 2H), 1.41–1.33 (m, 2H), 0.49 (d, J = 54.5 Hz, 8H) ppm.

^{13}C NMR (100 MHz, DMSO-d_6) δ 160.8, 158.0, 148.2, 142.6, 136.2, 133.8, 130.0, 129.4, 128.4, 126.9, 126.8, 125.0, 108.3, 13.9, 9.9, 8.6 ppm.

HRMS (*m/z*) (ESI): calcd for $C_{21}H_{19}NO$ 302.1539 [M+H$^+$]; found 302.1531.

6-(quinolin-2-yl)-5-(thiophen-2-yl)-[1,1'-biphenyl]-3-ol **(3x)**

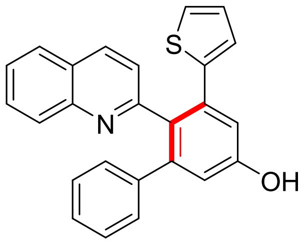

3x

The reaction of 2-methylquinoline (**1a**) (214.8 mg, 1.5 mmol), and 1-phenyl-5-(thiophen-2-yl)penta-1,4-diyn-3-one (**2n**) (118.2 mg, 0.5 mmol) at 100 °C for 7 h, isolated by column chromatography (silica gel, petroleum ether/AcOEt = 10/1), affords 165.1 mg (87%) of **3x** as light yellow solid. **m.p.** 207-208 °C.

^{1}H NMR (400 MHz, DMSO-d_6): δ 10.04 (s, 1H), 7.98 (d, J = 8.4 Hz, 1H), 7.82–7.76 (m, 2H), 7.66–7.61 (m, 1H), 7.48–7.42 (m, 1H), 7.24–7.16 (m, 1H), 7.12–7.08 (m, 4H), 7.06–7.02 (m, 3H), 6.88 (d, J = 2.4 Hz, 1H), 6.74–6.65 (m, 1H), 6.67–6.61 (m, 1H) ppm.

^{13}C NMR (100 MHz, DMSO-d_6): δ 159.5, 157.4, 147.4, 143.7, 142.9, 141.4, 135.4, 130.5, 129.8, 129.6, 129.3, 128.1, 128.0, 127.3, 127.0, 126.9, 126.8, 126.6, 125.4, 117.0, 116.2 ppm.

HRMS (*m/z*) (ESI): calcd for $C_{25}H_{18}NOS$ 380.1104 [M+H$^+$]; found 380.1086.

5-cyclopropyl-6-(quinolin-2-yl)-[1,1'-biphenyl]-3-ol **(3y)**

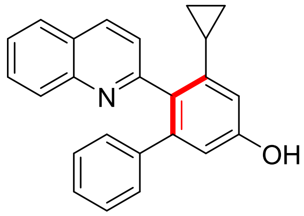

3y

The reaction of 2-methylquinoline (**1a**) (214.8 mg, 1.5 mmol), and 1-cyclopropyl-5-phenylpenta-1,4-diyn-3-one (**2o**) (97.1 mg, 0.5 mmol) at 100 °C for 9 h, isolated by column chromatography (silica gel, petroleum ether/AcOEt = 10/1), affords 113.0 mg (67%) of **3y** as light yellow solid. **m.p.** 118-120 °C.

^{1}H NMR (400 MHz, DMSO-d_6): δ 9.65 (s, 1H), 8.07 (d, J = 8.4 Hz, 1H), 7.96 (d, J = 8.4 Hz, 1H), 7.86 (d, J = 8.0 Hz, 1H), 7.70–7.62 (m, 1H), 7.54–7.48 (m, 1H), 7.12 (d, J = 8.4 Hz, 1H), 7.08–7.02 (m, 5H), 6.66 (d, J = 2.4 Hz, 1H), 6.47 (d, J = 2.3 Hz, 1H), 1.71–1.62 (m, 1H), 0.58 (s, 4H) ppm.

^{13}C NMR (100 MHz, DMSO-d_6): δ 160.3, 157.7, 147.6, 143.8, 142.3, 141.8, 135.5, 132.0, 129.7, 129.6, 129.3, 128.1, 128.1, 126.8, 126.6, 126.4, 125.2, 114.4, 110.3, 13.8, 9.3 ppm.

HRMS (*m/z*) (ESI): calcd for $C_{24}H_{20}NO$ 338.1539 [M+H$^+$]; found 338.1537.

6-(6-ethoxyquinolin-2-yl)-5-(naphthalen-2-yl)-[1,1'-biphenyl]-3-ol **(3z)**

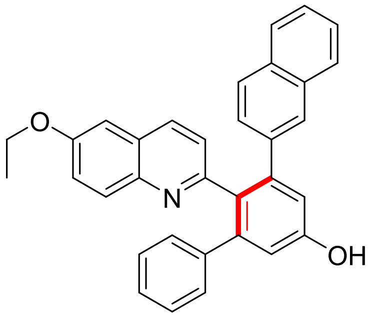

3z

The reaction of 2-methylquinoline (**1a**) (214.8 mg, 1.5 mmol), and 1-(naphthalen-2-yl)-5-phenylpenta-1,4-diyn-3-one (**2p**) (140.2 mg, 0.5 mmol) at 100 °C for 8 h, isolated by column chromatography (silica gel, petroleum ether/AcOEt = 10/1), affords 147.3 mg (63%) of **3z** as light yellow solid. **m.p.** 136-137 °C.

^{1}H NMR (400 MHz, DMSO-d_6): δ 9.93 (s, 1H), 7.76 (d, J = 1.1 Hz, 1H), 7.71–7.63 (m, 3H), 7.52–7.46 (m, 2H), 7.42–7.36 (m, 2H), 7.16–7.11 (m, 1H), 7.10–7.04 (m, 7H), 6.95–6.88 (m, 2H), 6.88 (d, J = 2.5 Hz, 1H), 4.03 (q, J = 7.6 Hz, 2H), 1.32 (t, J = 7.2 Hz, 3H) ppm.

^{13}C NMR (100 MHz, DMSO-d_6): δ 157.2, 156.9, 156.7, 143.5, 143.2, 143.2, 141.8, 139.5, 133.8, 133.0, 131.8, 130.8, 130.5, 128.3, 128.2, 128.1, 127.8, 127.1, 127.0, 126.8, 126.5, 126.3, 125.7, 122.1, 116.7, 116.5, 106.5, 63.8, 15.0 ppm.

HRMS (*m/z*) (ESI): calcd for $C_{33}H_{26}NO_2$ 468.1958 [M+H$^+$]; found 468.1956.

Copies of ^{1}H NMR and ^{13}C NMR spectra of all compounds

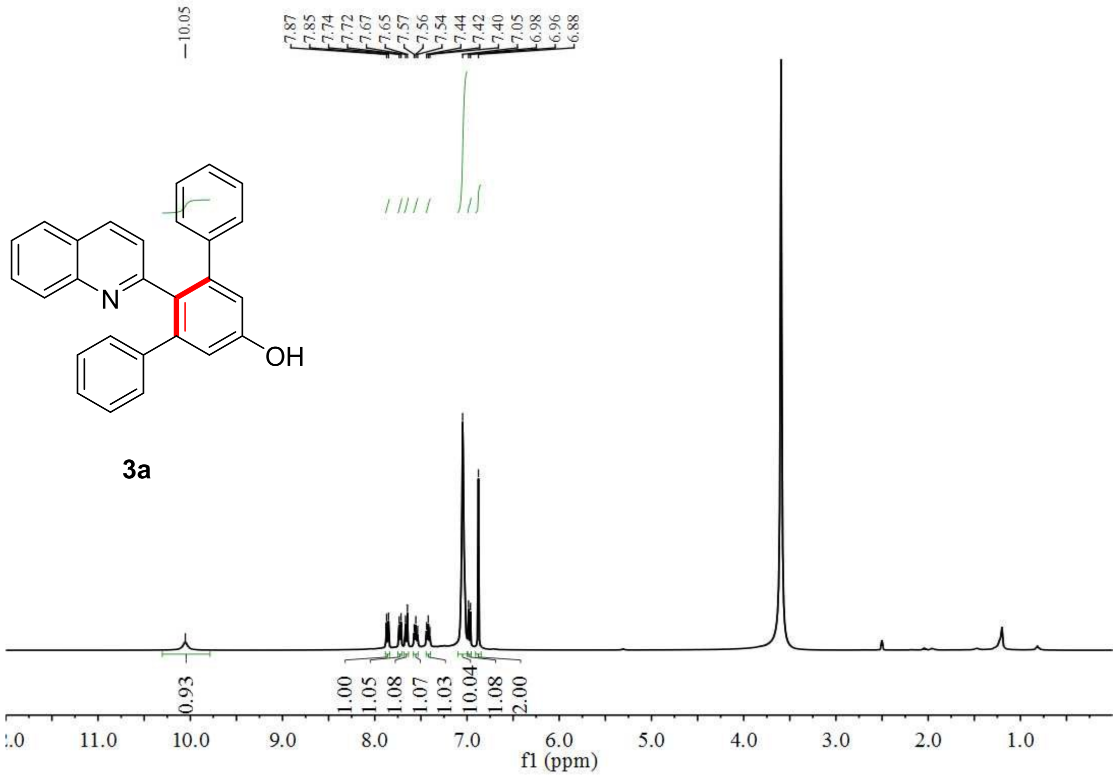

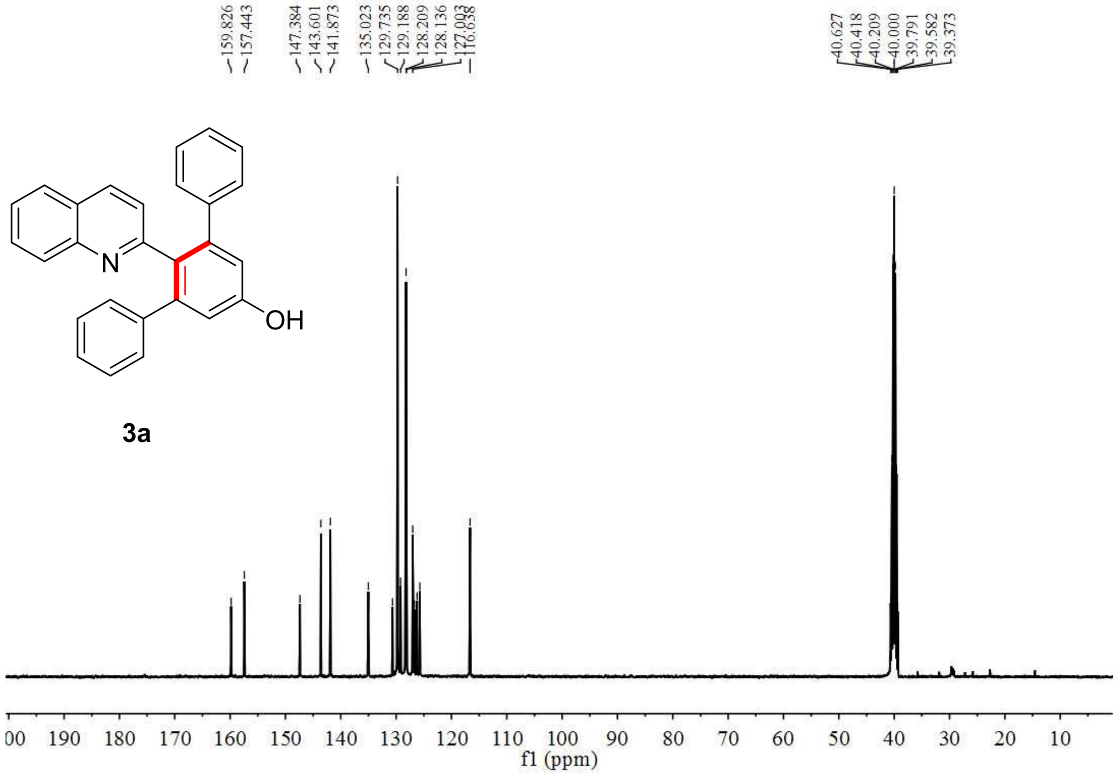

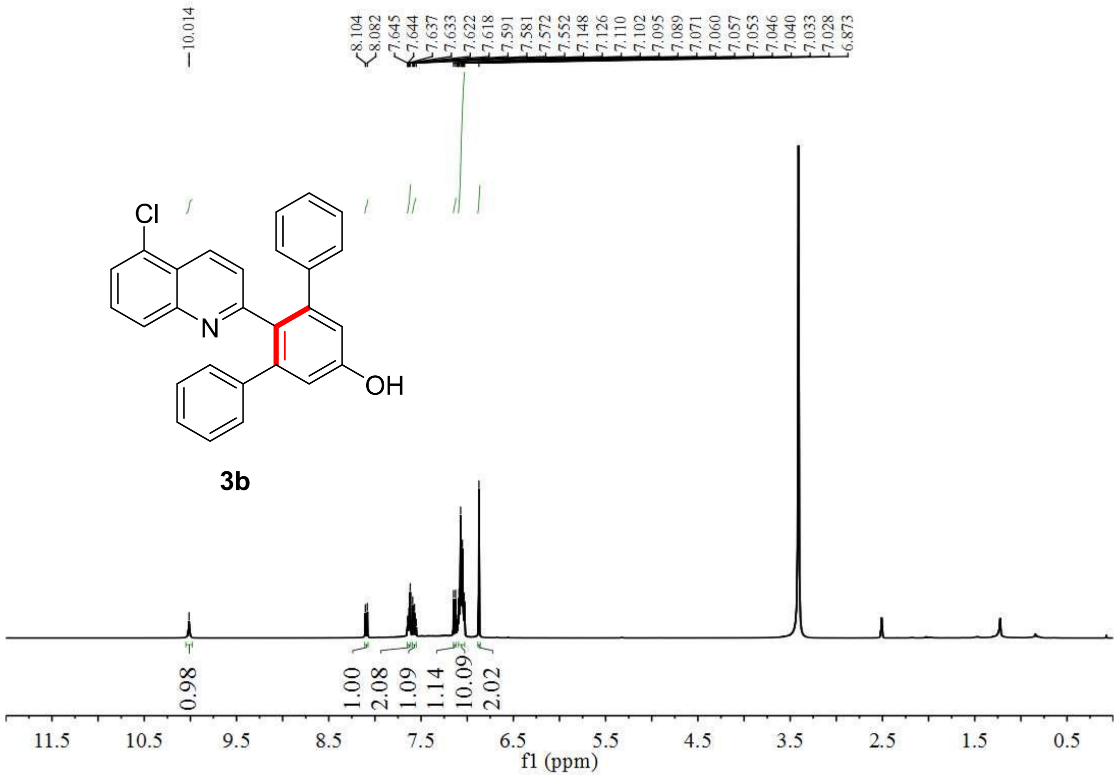

10.014
8.104
8.082
7.645
7.644
7.637
7.633
7.622
7.618
7.591
7.581
7.572
7.552
7.148
7.126
7.110
7.102
7.095
7.089
7.071
7.060
7.057
7.053
7.046
7.040
7.033
7.028
6.873
Cl
N
OH
3b
0.98
1.00
2.08
1.09
1.14
10.09
2.02
11.5
10.5
9.5
8.5
7.5
6.5
5.5
4.5
3.5
2.5
1.5
0.5
f1 (ppm)

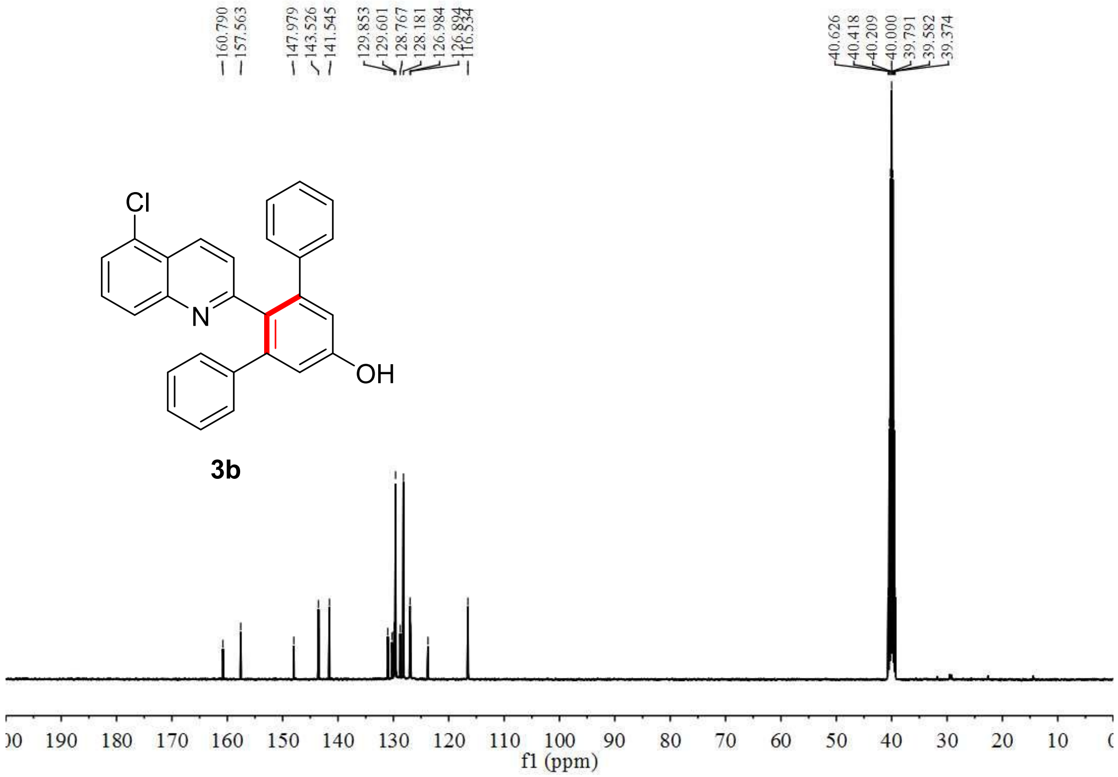

160.790
157.563
147.979
143.526
141.545
129.853
129.601
128.767
128.181
126.984
126.894
116.534
40.626
40.418
40.209
40.000
39.791
39.582
39.374
Cl
N
OH
3b
190
180
170
160
150
140
130
120
110
100
90
80
70
60
50
40
30
20
10
f1 (ppm)

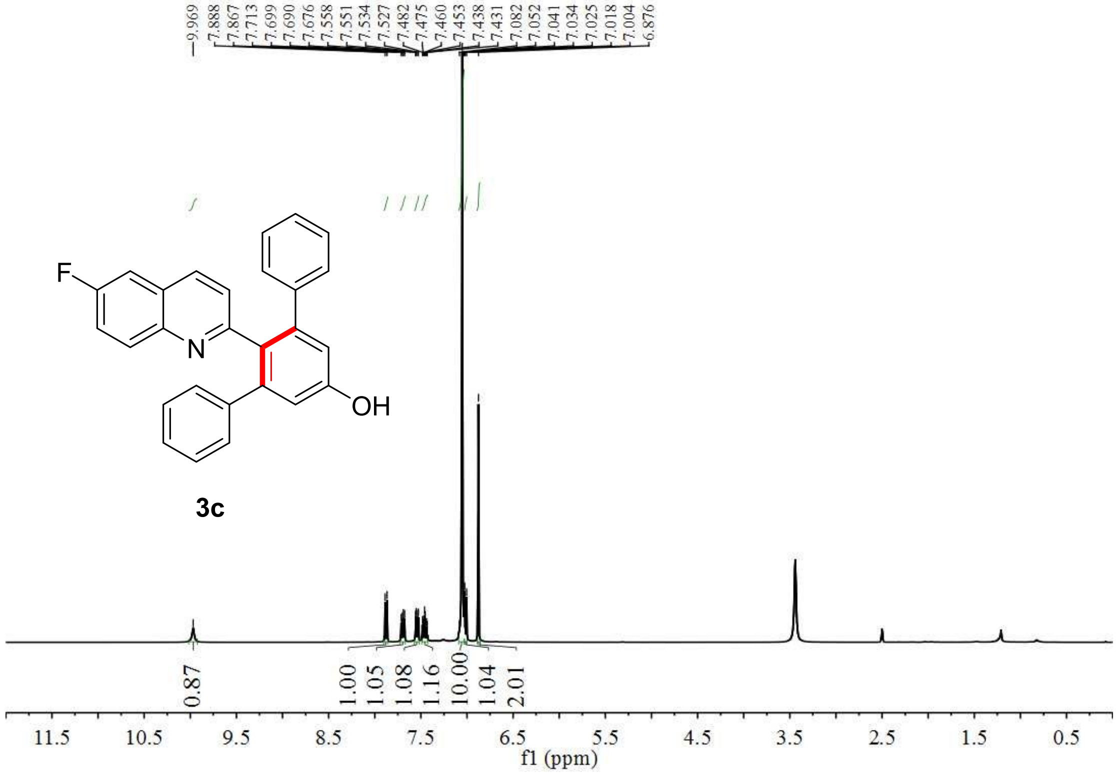
9.969
7.888
7.867
7.713
7.699
7.690
7.676
7.558
7.551
7.534
7.527
7.482
7.475
7.460
7.453
7.438
7.431
7.082
7.052
7.041
7.034
7.025
7.018
7.004
6.876
F
N
OH
3c
0.87
1.00
1.05
1.08
1.16
10.00
1.04
2.01
11.5 10.5 9.5 8.5 7.5 6.5 5.5 4.5 3.5 2.5 1.5 0.5
f1 (ppm)

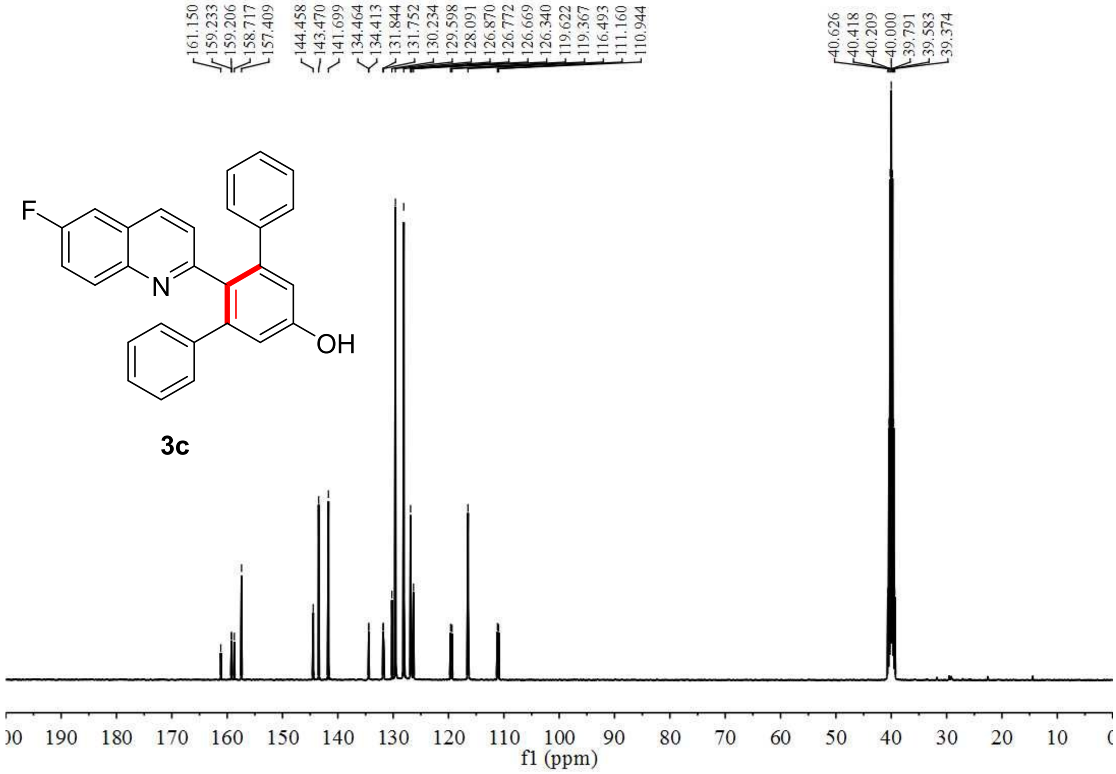
161.150
159.233
159.206
158.717
157.409
144.458
143.470
141.699
134.464
134.413
131.844
131.752
130.234
129.598
128.091
126.870
126.772
126.669
126.340
119.622
119.367
116.493
111.160
110.944
40.626
40.418
40.209
40.000
39.791
39.583
39.374
F
N
OH
3c
190 180 170 160 150 140 130 120 110 100 90 80 70 60 50 40 30 20 10
f1 (ppm)

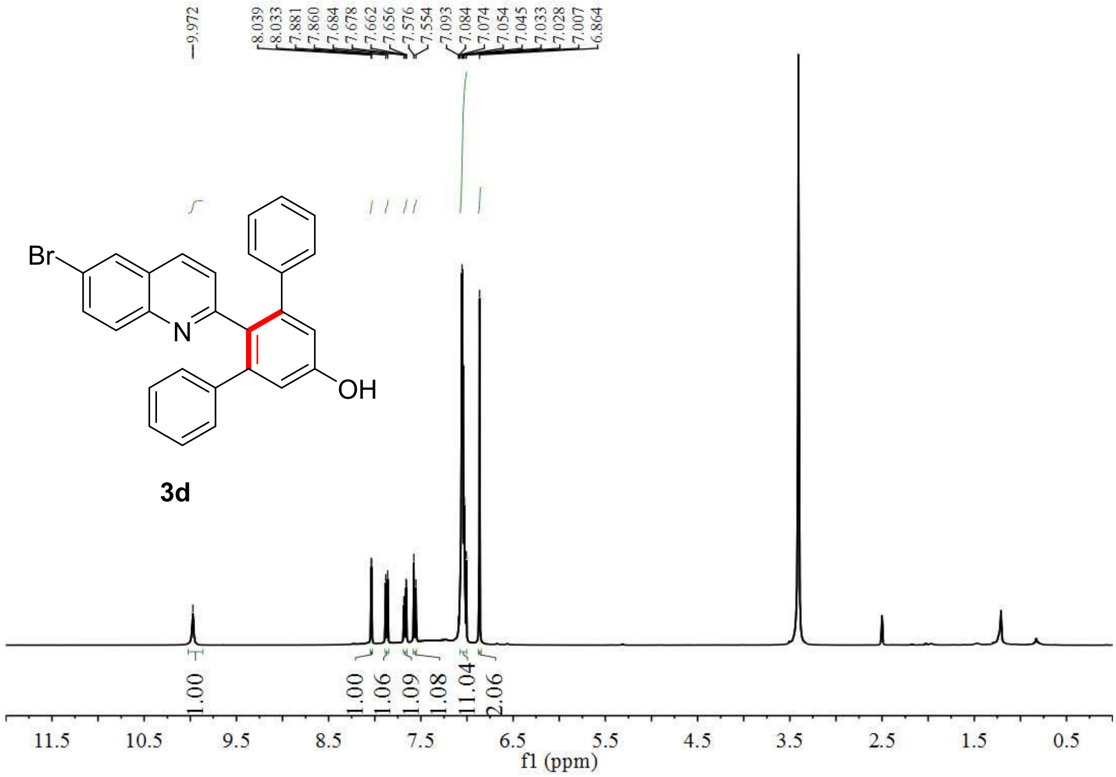
9.972
8.039
8.033
7.881
7.860
7.684
7.678
7.662
7.656
7.576
7.554
7.093
7.084
7.074
7.054
7.045
7.033
7.028
7.007
6.864
Br
N
OH
3d
1.00
1.00
1.06
1.09
1.08
11.04
2.06
11.5
10.5
9.5
8.5
7.5
6.5
5.5
4.5
3.5
2.5
1.5
0.5
f1 (ppm)

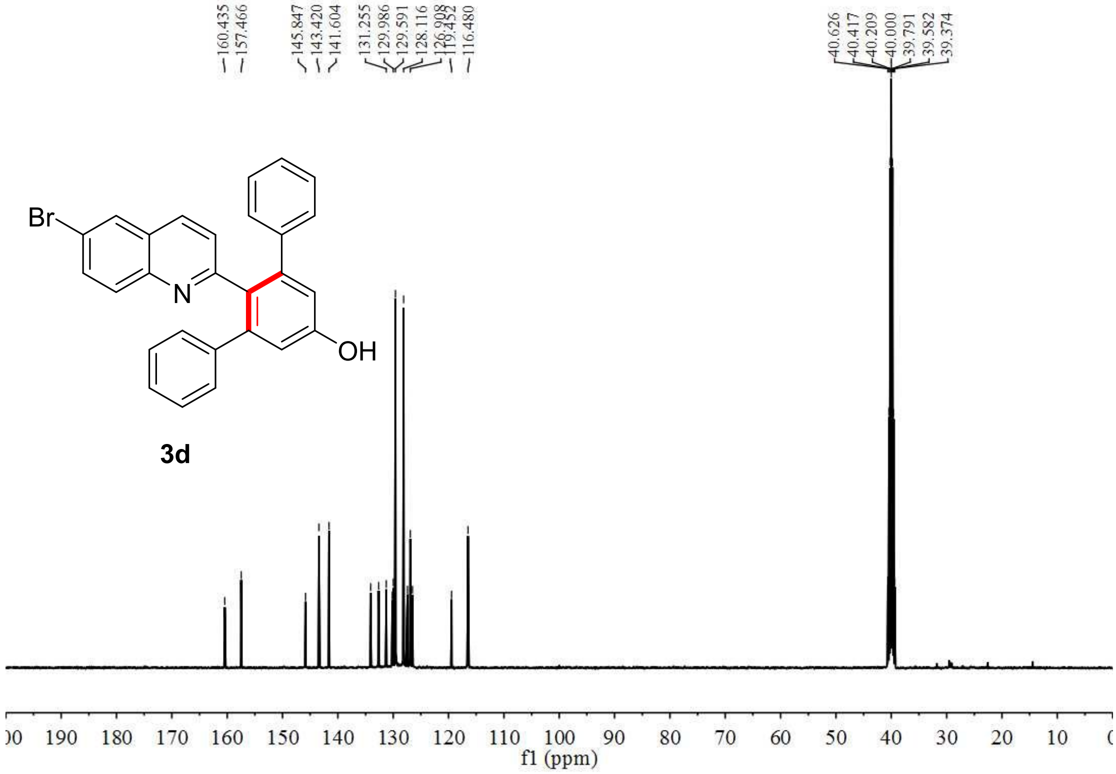
160.435
157.466
145.847
143.420
141.604
131.255
129.986
129.591
128.116
119.452
116.480
40.626
40.417
40.209
40.000
39.791
39.582
39.374
Br
N
OH
3d
190
180
170
160
150
140
130
120
110
100
90
80
70
60
50
40
30
20
10
f1 (ppm)

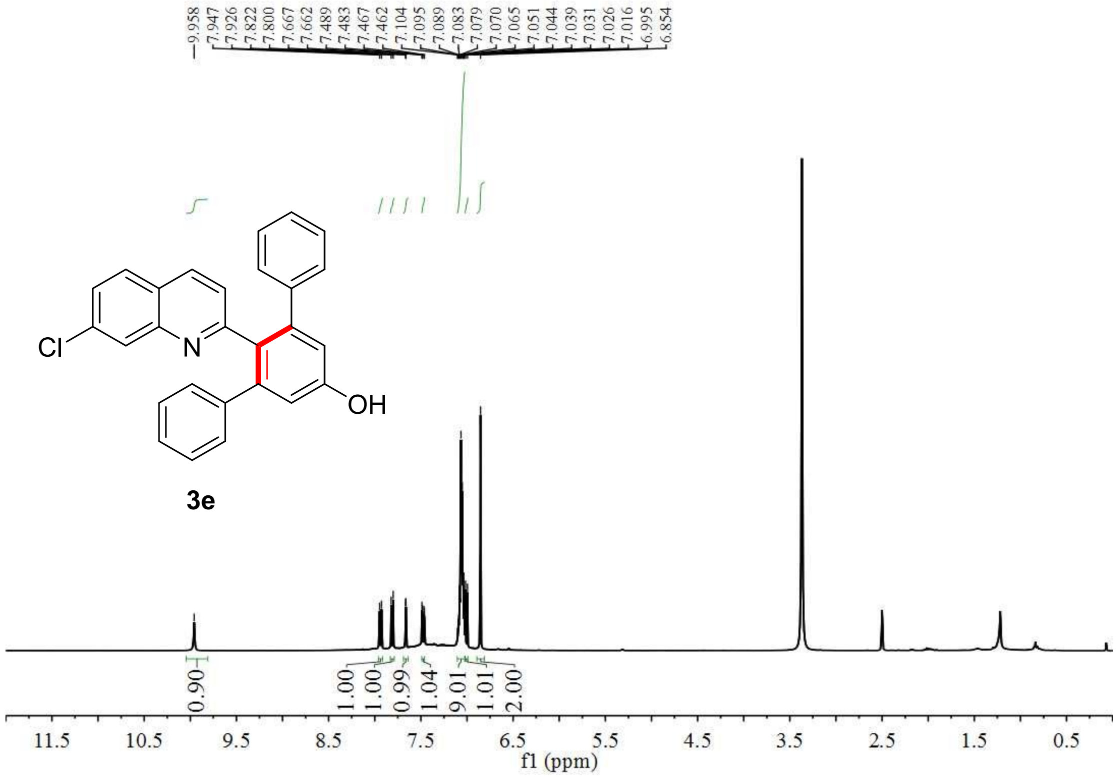
9.958
7.947
7.926
7.822
7.800
7.667
7.662
7.489
7.483
7.467
7.462
7.104
7.095
7.089
7.083
7.079
7.070
7.065
7.051
7.044
7.039
7.031
7.026
7.016
6.995
6.854
Cl
N
OH
3e
0.90
1.00
1.00
0.99
1.04
9.01
1.01
2.00
11.5
10.5
9.5
8.5
7.5
6.5
5.5
4.5
3.5
2.5
1.5
0.5
f1 (ppm)

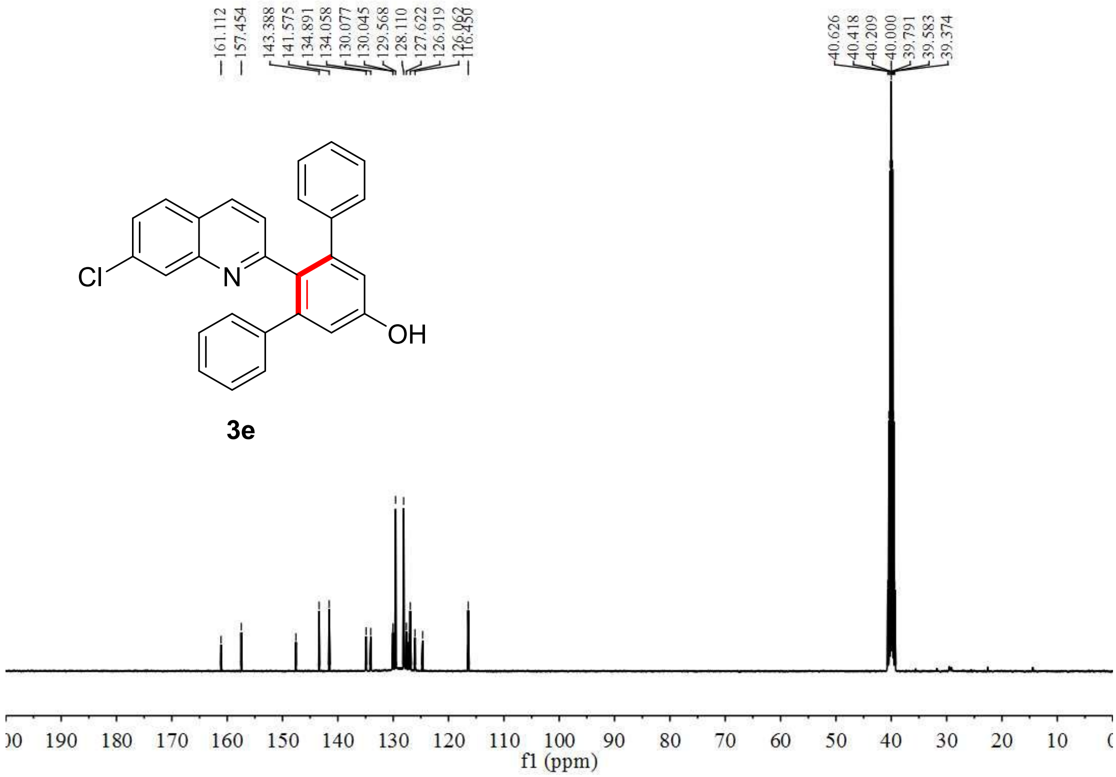
161.112
157.454
143.388
141.575
134.891
134.058
130.077
130.045
129.568
128.110
127.622
126.919
126.062
116.450
40.626
40.418
40.209
40.000
39.791
39.583
39.374
Cl
N
OH
3e
190
180
170
160
150
140
130
120
110
100
90
80
70
60
50
40
30
20
10
f1 (ppm)

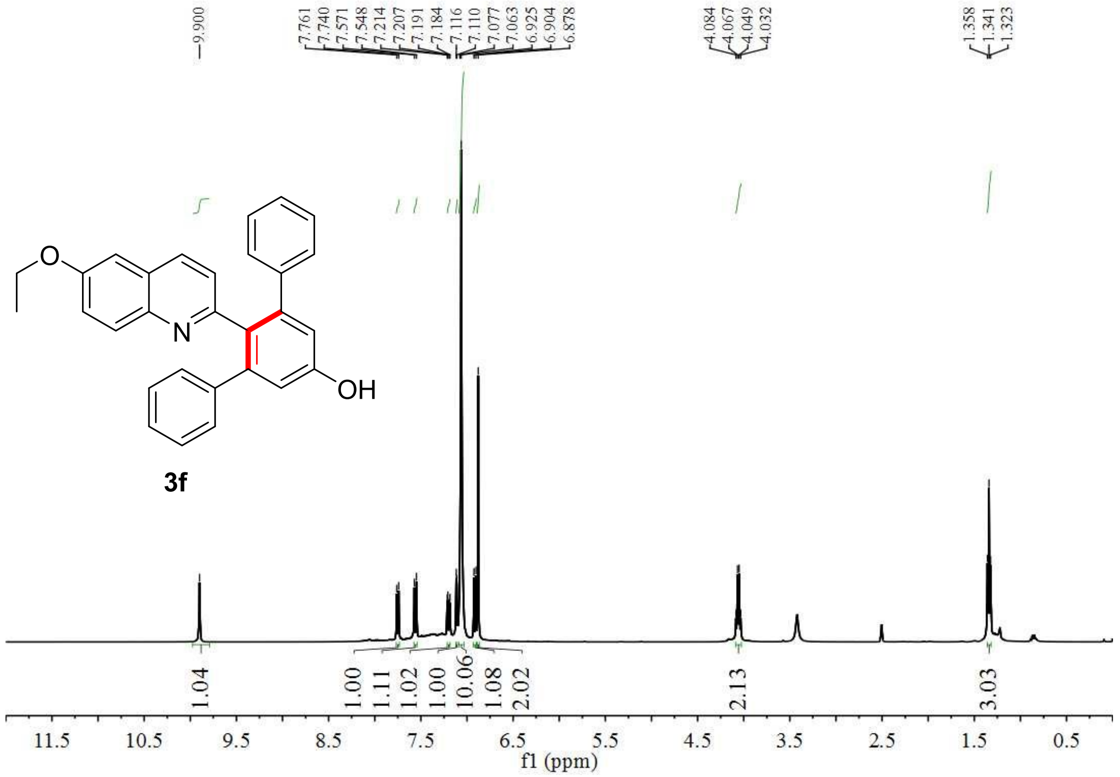
9.900
7.761
7.740
7.571
7.548
7.214
7.207
7.191
7.184
7.116
7.110
7.077
7.063
6.925
6.904
6.878
4.084
4.067
4.049
4.032
1.358
1.341
1.323
O
N
OH
3f
1.04
1.00
1.11
1.02
1.00
10.06
1.08
2.02
2.13
3.03
f1 (ppm)

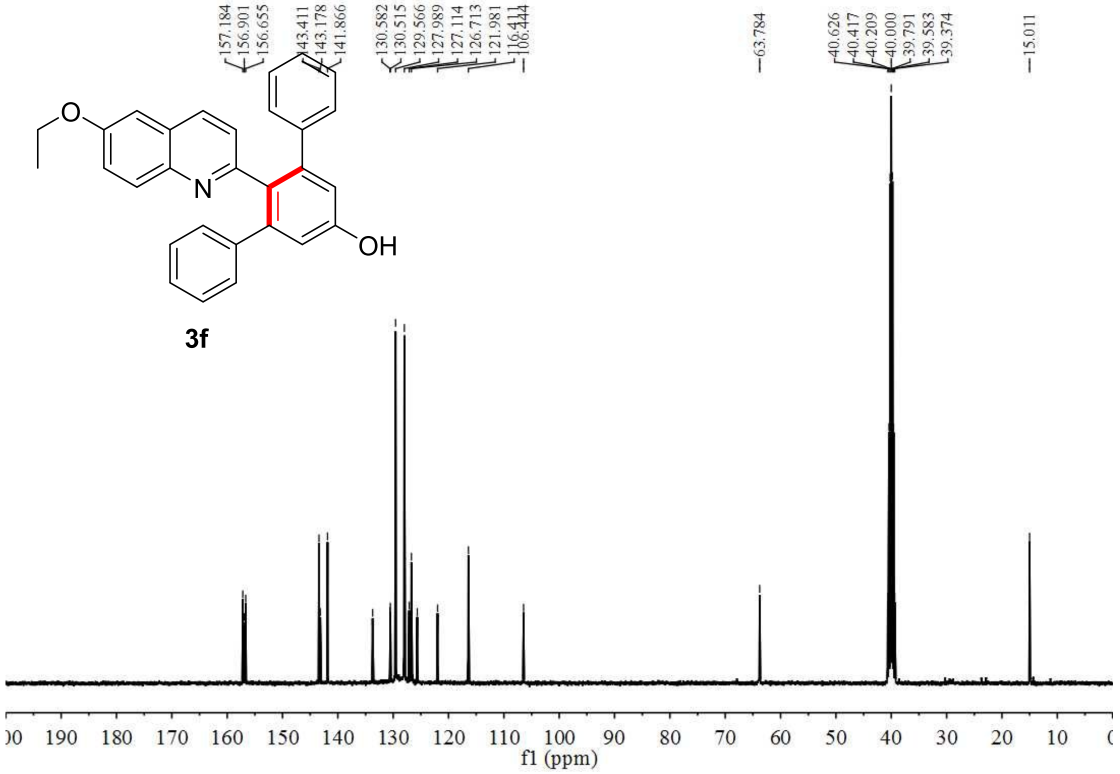
157.184
156.901
156.655
143.411
143.178
141.866
130.582
130.515
129.566
127.989
127.114
126.713
121.981
116.411
106.444
63.784
40.626
40.417
40.209
40.000
39.791
39.583
39.374
15.011
O
N
OH
3f
f1 (ppm)

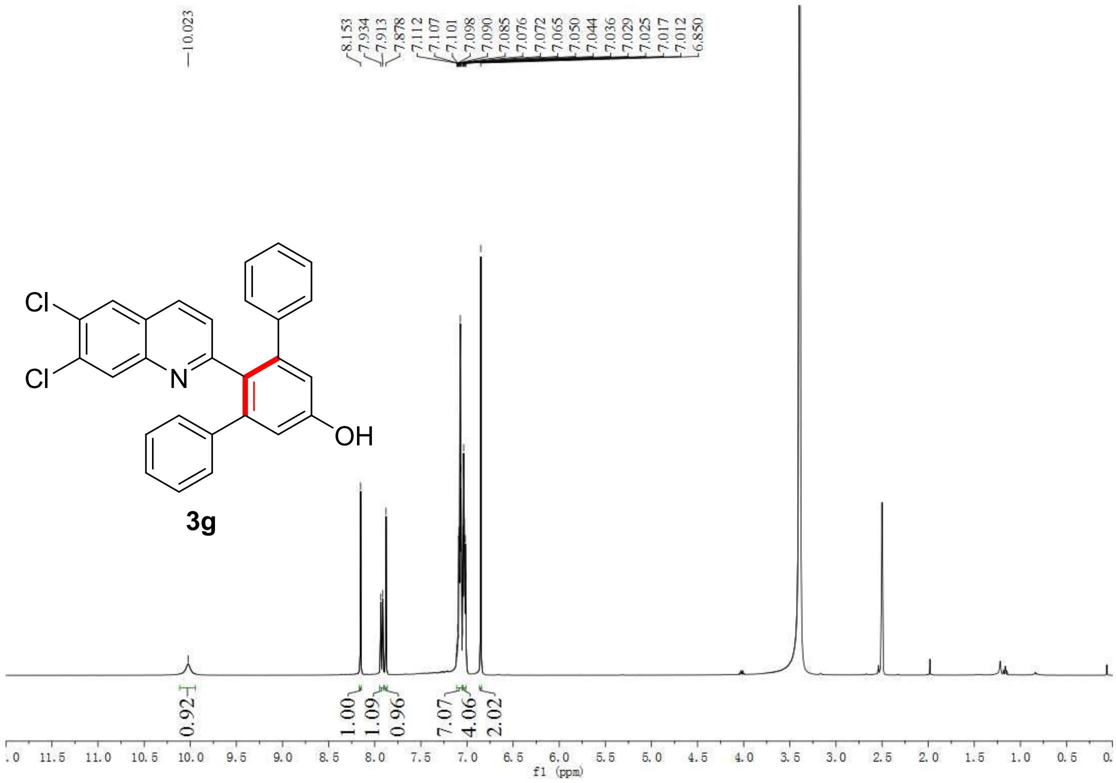
10.023
8.153
7.934
7.913
7.878
7.112
7.107
7.101
7.098
7.090
7.085
7.076
7.072
7.065
7.050
7.044
7.036
7.029
7.025
7.017
7.012
6.850
Cl
Cl
N
OH
3g
0.92
1.00
1.09
0.96
7.07
4.06
2.02
f1 (ppm)

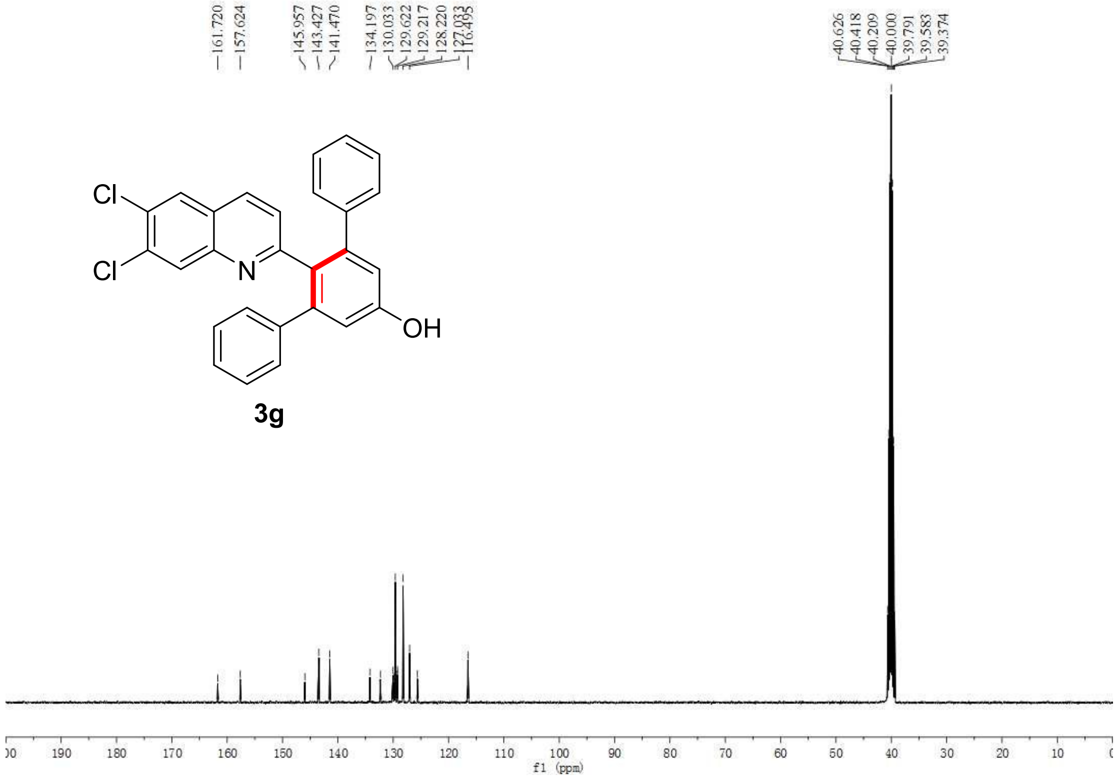
161.720
157.624
145.957
143.427
141.470
134.197
130.033
129.622
129.217
128.220
127.033
116.495
40.626
40.418
40.209
40.000
39.791
39.583
39.374
Cl
Cl
N
OH
3g
f1 (ppm)

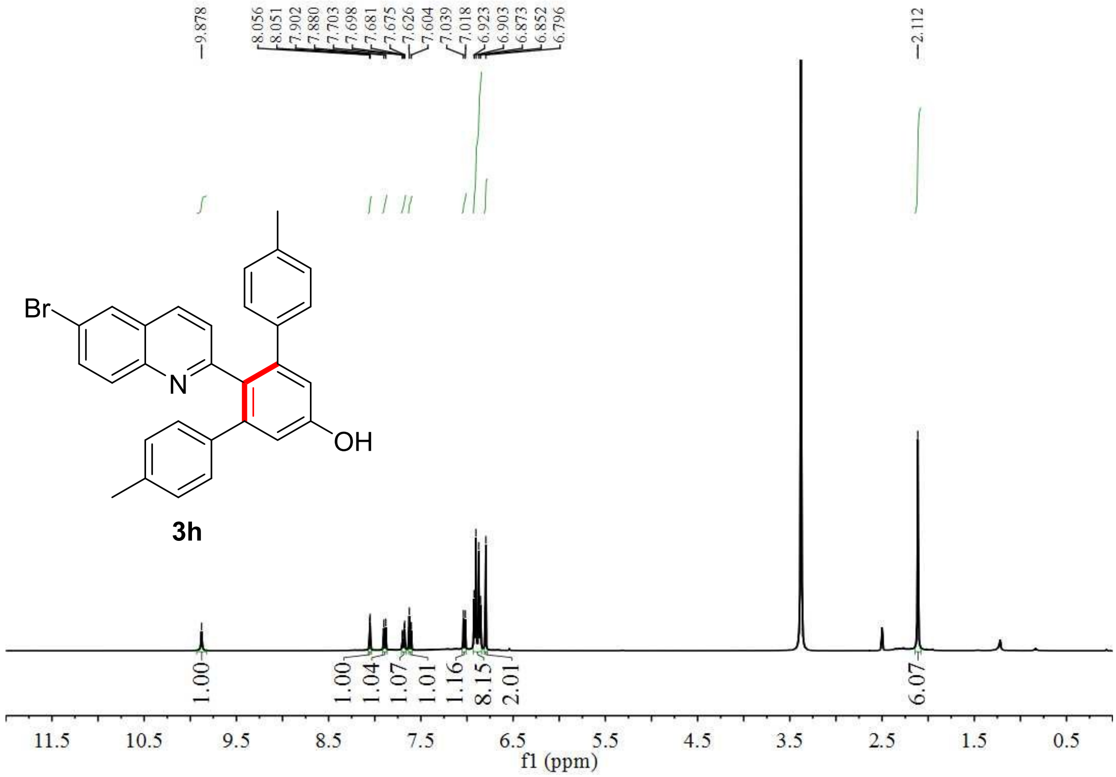
9.878
8.056
8.051
7.902
7.880
7.703
7.698
7.681
7.675
7.626
7.604
7.039
7.018
6.923
6.903
6.873
6.852
6.796
2.112
Br
N
OH
3h
1.00
1.00
1.04
1.07
1.01
1.16
8.15
2.01
6.07
11.5
10.5
9.5
8.5
7.5
6.5
5.5
4.5
3.5
2.5
1.5
0.5
f1 (ppm)

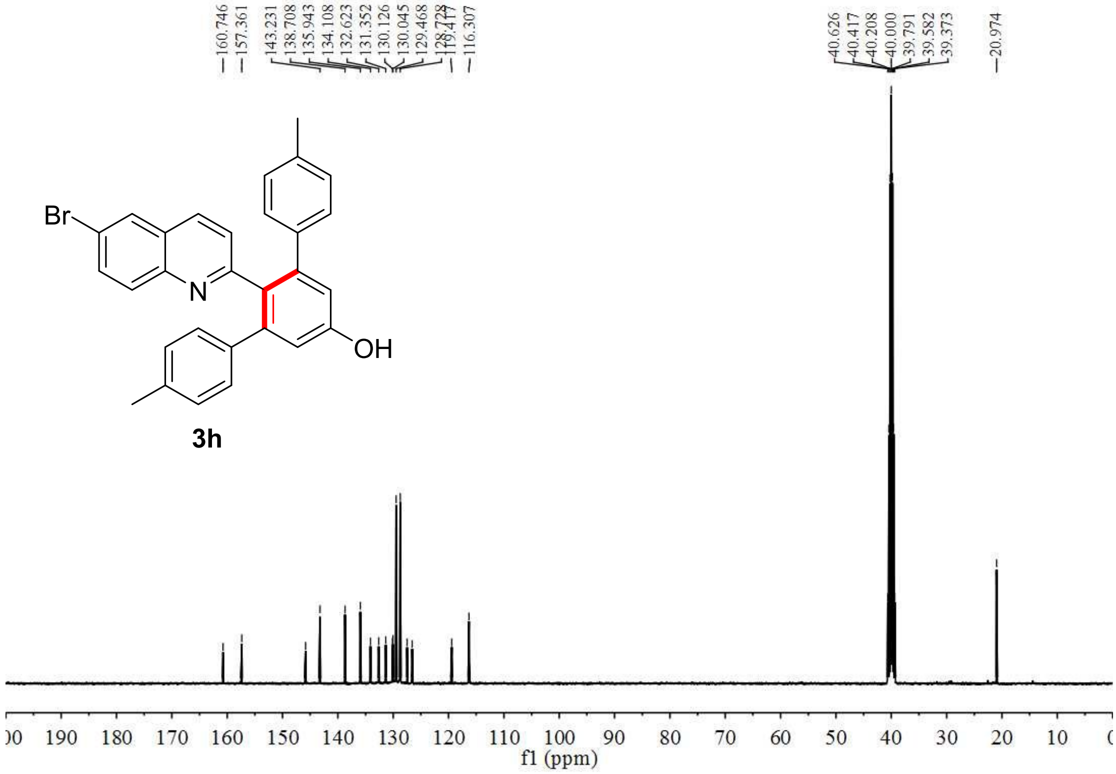
160.746
157.361
143.231
138.708
135.943
134.108
132.623
131.352
130.126
130.045
129.468
119.417
116.307
40.626
40.417
40.208
40.000
39.791
39.582
39.373
20.974
Br
N
OH
3h
190
180
170
160
150
140
130
120
110
100
90
80
70
60
50
40
30
20
10
f1 (ppm)

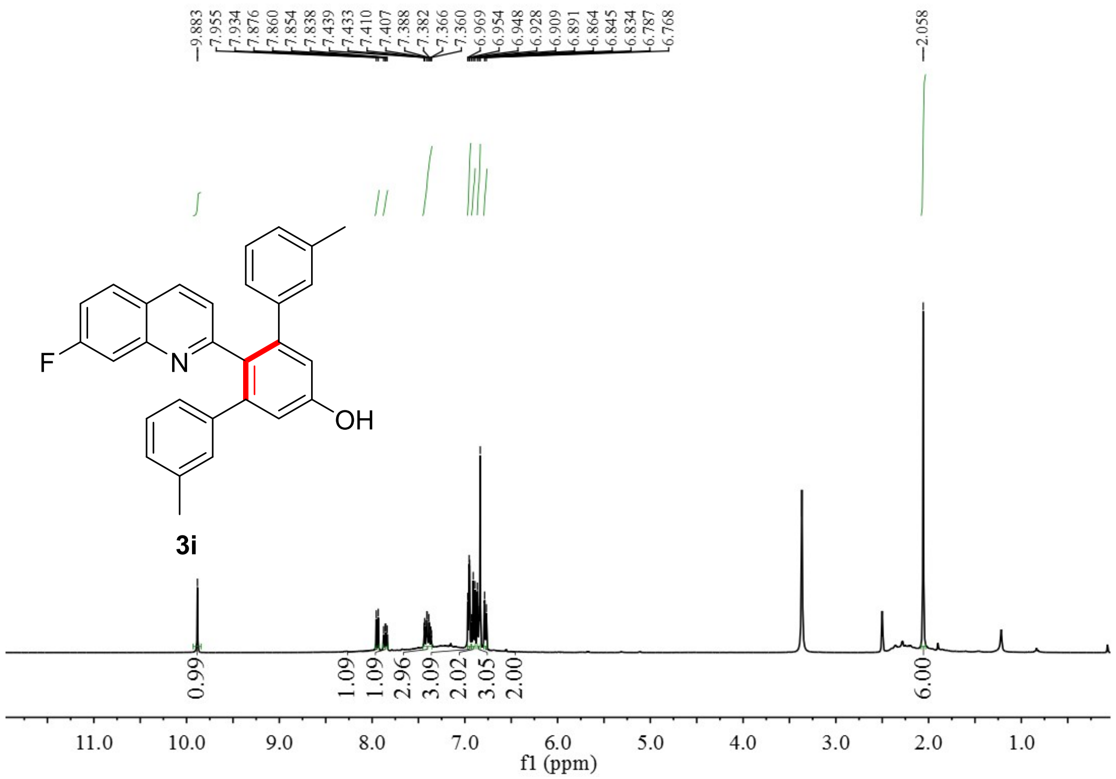
9.883
7.955 7.934 7.876 7.860 7.854 7.838 7.439 7.433 7.410 7.407 7.388 7.382 7.366 7.360 6.969 6.954 6.948 6.928 6.909 6.891 6.864 6.845 6.834 6.787 6.768
2.058
F
N
OH
3i
0.99 1.09 1.09 2.96 3.09 2.02 3.05 2.00 6.00
11.0 10.0 9.0 8.0 7.0 6.0 5.0 4.0 3.0 2.0 1.0
f1 (ppm)

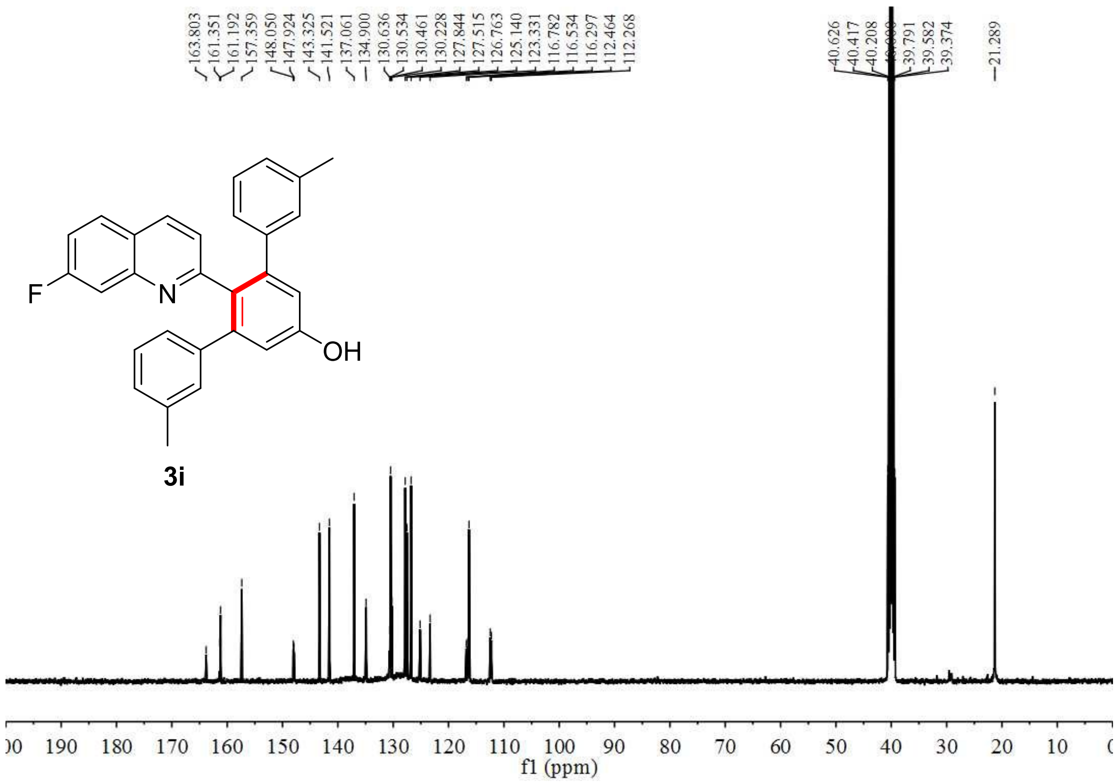
163.803 161.351 161.192 157.359 148.050 147.924 143.325 141.521 137.061 134.900 130.636 130.534 130.461 130.228 127.844 127.515 126.763 125.140 123.331 116.782 116.534 116.297 112.464 112.268
40.626 40.417 40.208 39.791 39.582 39.374
21.289
F
N
OH
3i
190 180 170 160 150 140 130 120 110 100 90 80 70 60 50 40 30 20 10
f1 (ppm)

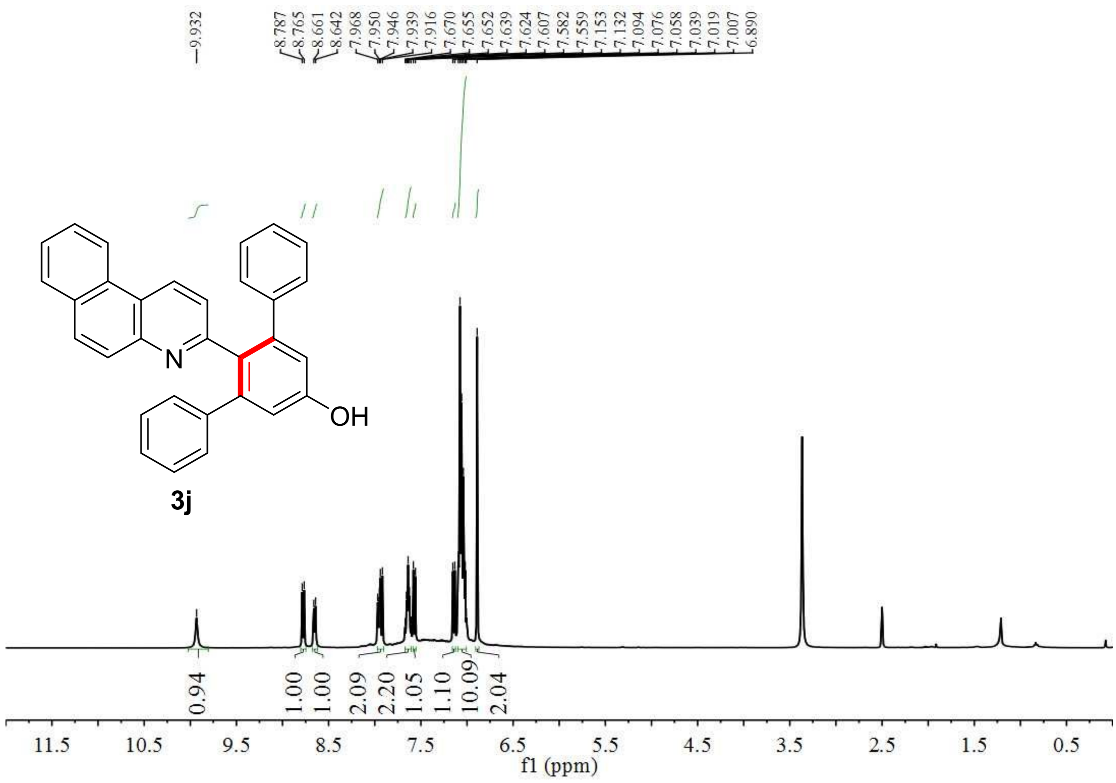
9.932
8.787 8.765 8.661 8.642 7.968 7.950 7.946 7.939 7.916 7.670 7.655 7.652 7.639 7.624 7.607 7.582 7.559 7.153 7.132 7.094 7.076 7.058 7.039 7.019 7.007 6.890
N
OH
3j
0.94 1.00 1.00 2.09 2.20 1.05 1.10 10.09 2.04
11.5 10.5 9.5 8.5 7.5 6.5 5.5 4.5 3.5 2.5 1.5 0.5
f1 (ppm)

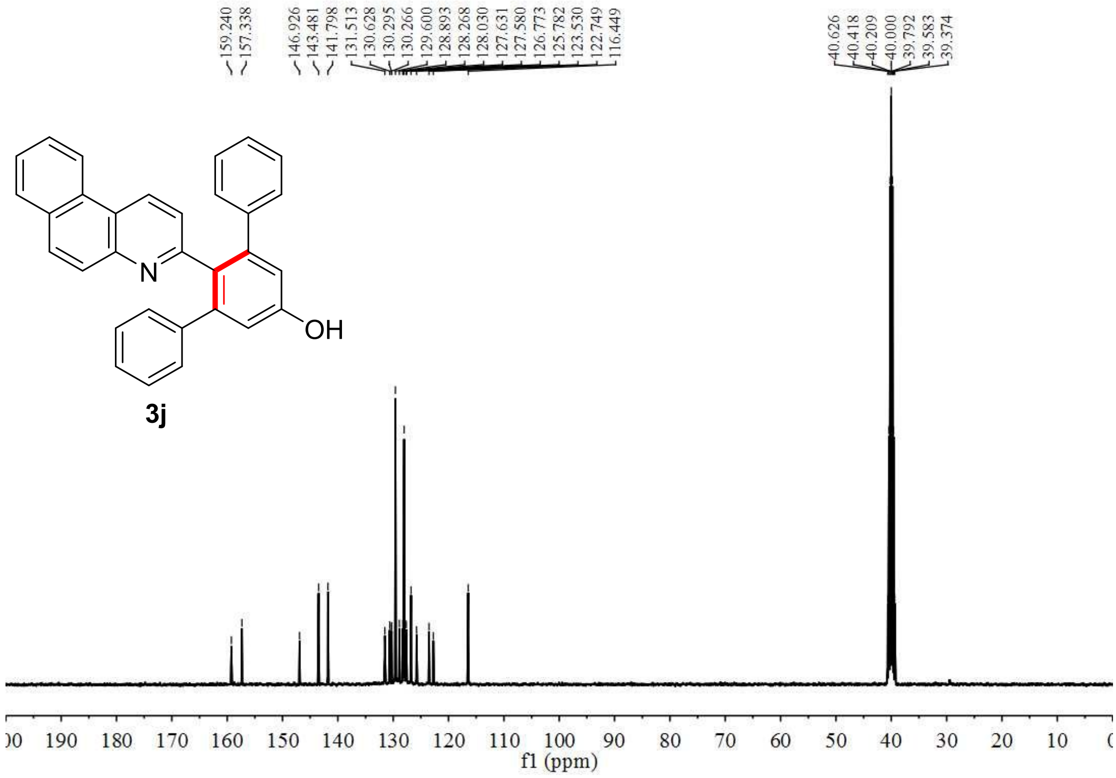
159.240 157.338
146.926 143.481 141.798 131.513 130.628 130.295 130.266 129.600 128.893 128.268 128.030 127.631 127.580 126.773 125.782 123.530 122.749 116.449
40.626 40.418 40.209 40.000 39.792 39.583 39.374
N
OH
3j
190 180 170 160 150 140 130 120 110 100 90 80 70 60 50 40 30 20 10
f1 (ppm)

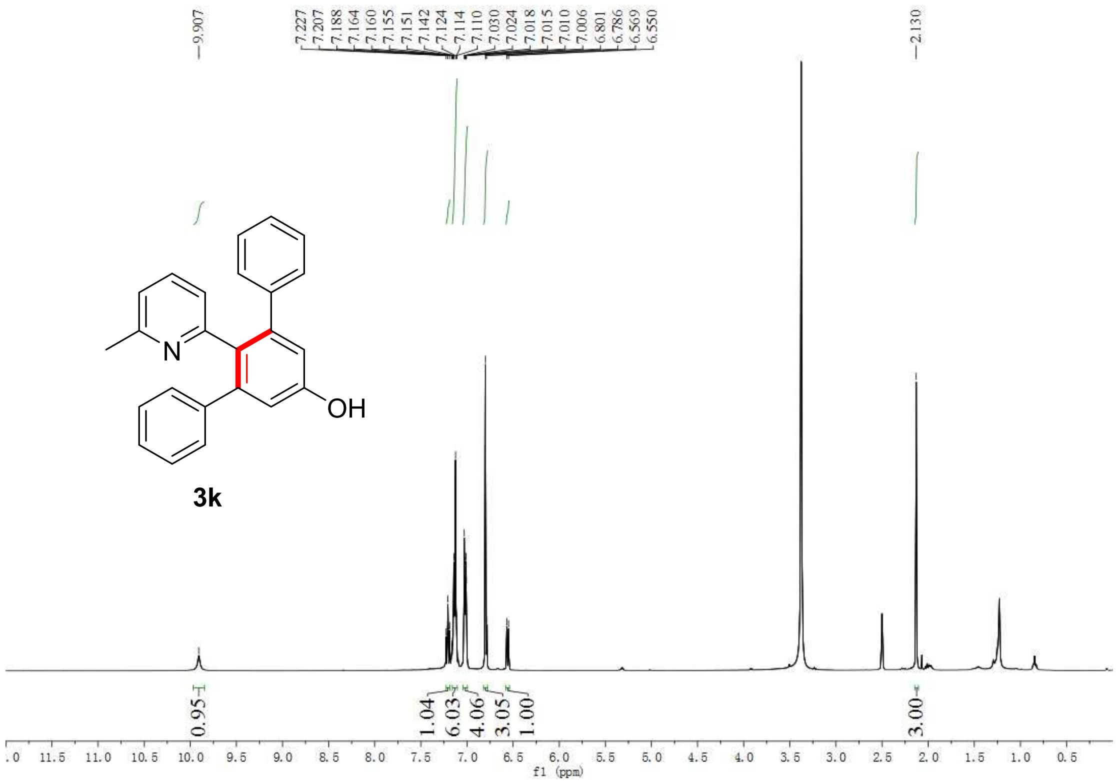
9.907
7.227
7.207
7.188
7.164
7.160
7.155
7.151
7.142
7.124
7.114
7.110
7.030
7.024
7.018
7.015
7.010
7.006
6.801
6.786
6.569
6.550
2.130
N
OH
3k
0.95
1.04
6.03
4.06
3.05
1.00
3.00
f1 (ppm)

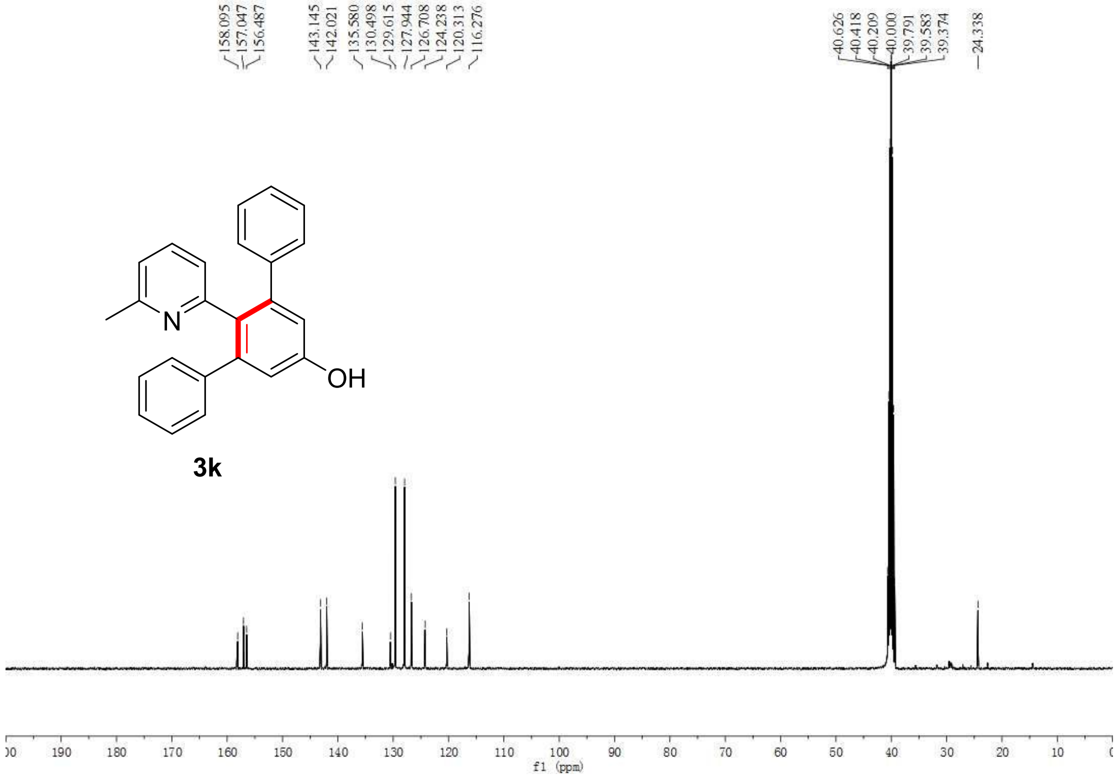
158.095
157.047
156.487
143.145
142.021
135.580
130.498
129.615
127.944
126.708
124.238
120.313
116.276
40.626
40.418
40.209
40.000
39.791
39.583
39.374
24.338
N
OH
3k
f1 (ppm)

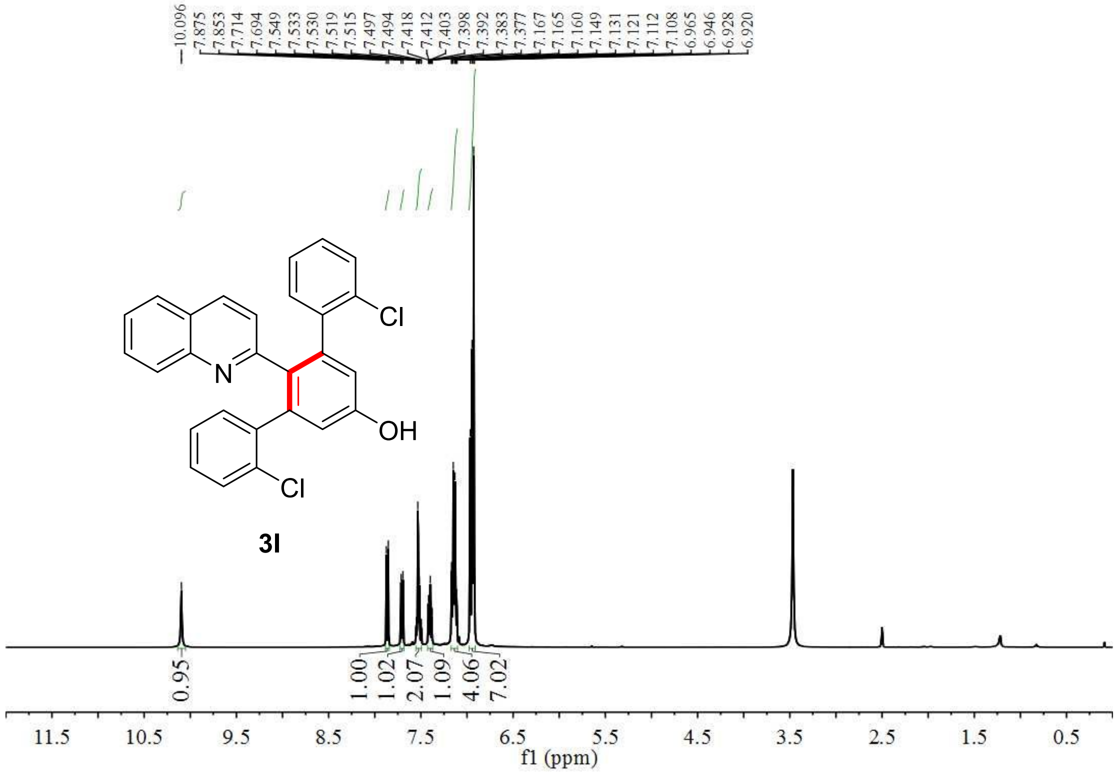

3l

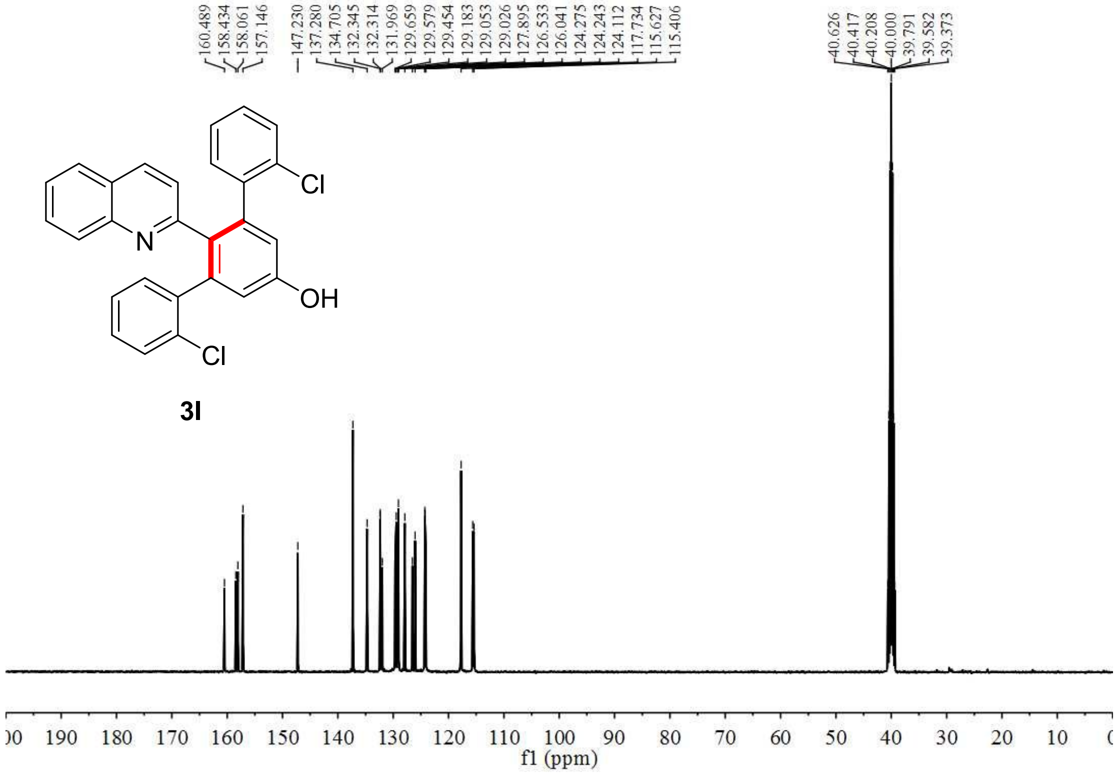

3l

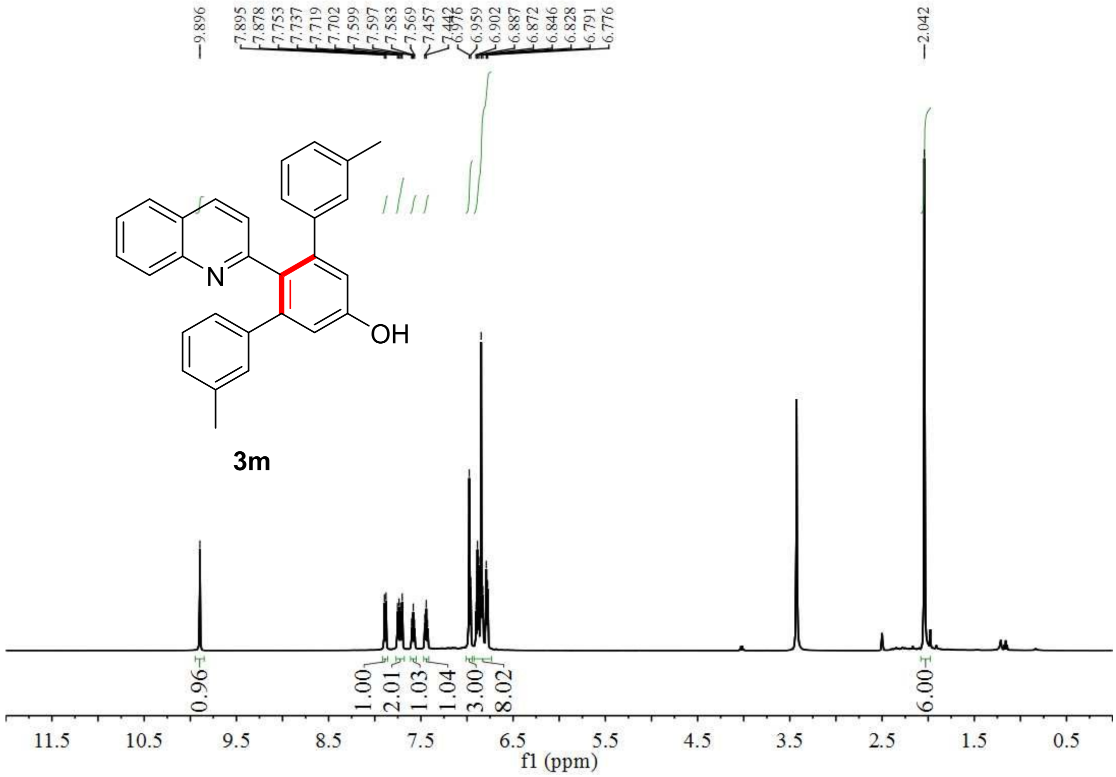
9.896
7.895
7.878
7.753
7.737
7.719
7.702
7.599
7.597
7.583
7.569
7.457
7.442
6.976
6.959
6.902
6.887
6.872
6.846
6.828
6.791
6.776
2.042
N
OH
3m
0.96
1.00
2.01
1.03
1.04
3.00
8.02
6.00
11.5 10.5 9.5 8.5 7.5 6.5 5.5 4.5 3.5 2.5 1.5 0.5
f1 (ppm)

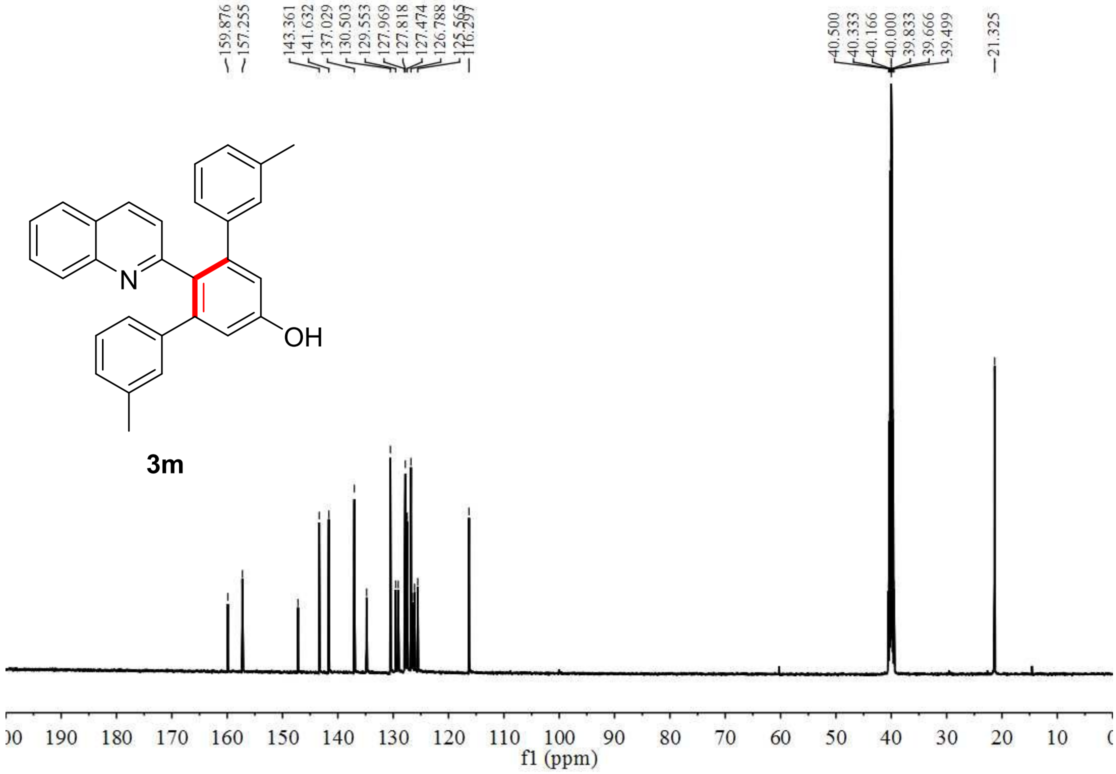
159.876
157.255
143.361
141.632
137.029
130.503
129.553
127.969
127.818
127.474
126.788
125.565
116.297
40.500
40.333
40.166
40.000
39.833
39.666
39.499
21.325
N
OH
3m
190 180 170 160 150 140 130 120 110 100 90 80 70 60 50 40 30 20 10
f1 (ppm)

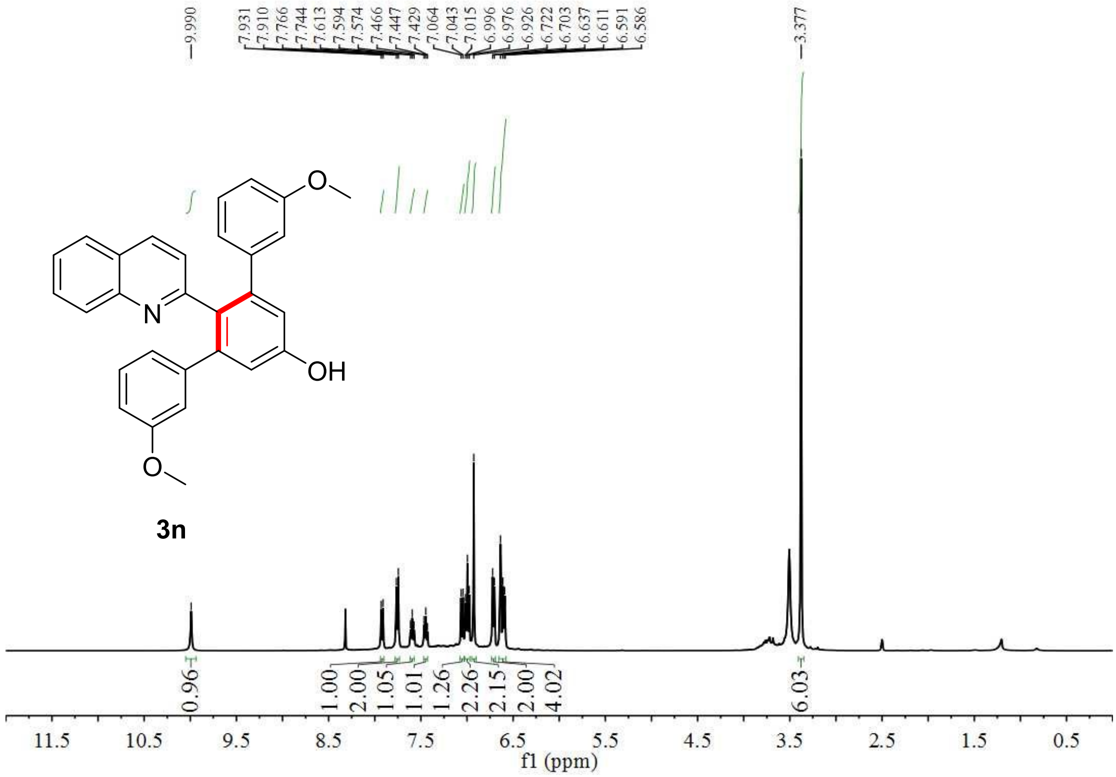
9.990
7.931 7.910 7.766 7.744 7.613 7.594 7.574 7.466 7.447 7.429 7.064 7.043 7.015 6.996 6.976 6.926 6.722 6.703 6.637 6.611 6.591 6.586
3.377
N
OH
3n
0.96
1.00 2.00 1.05 1.01 1.26 2.26 2.15 2.00 4.02
6.03
11.5 10.5 9.5 8.5 7.5 6.5 5.5 4.5 3.5 2.5 1.5 0.5
f1 (ppm)

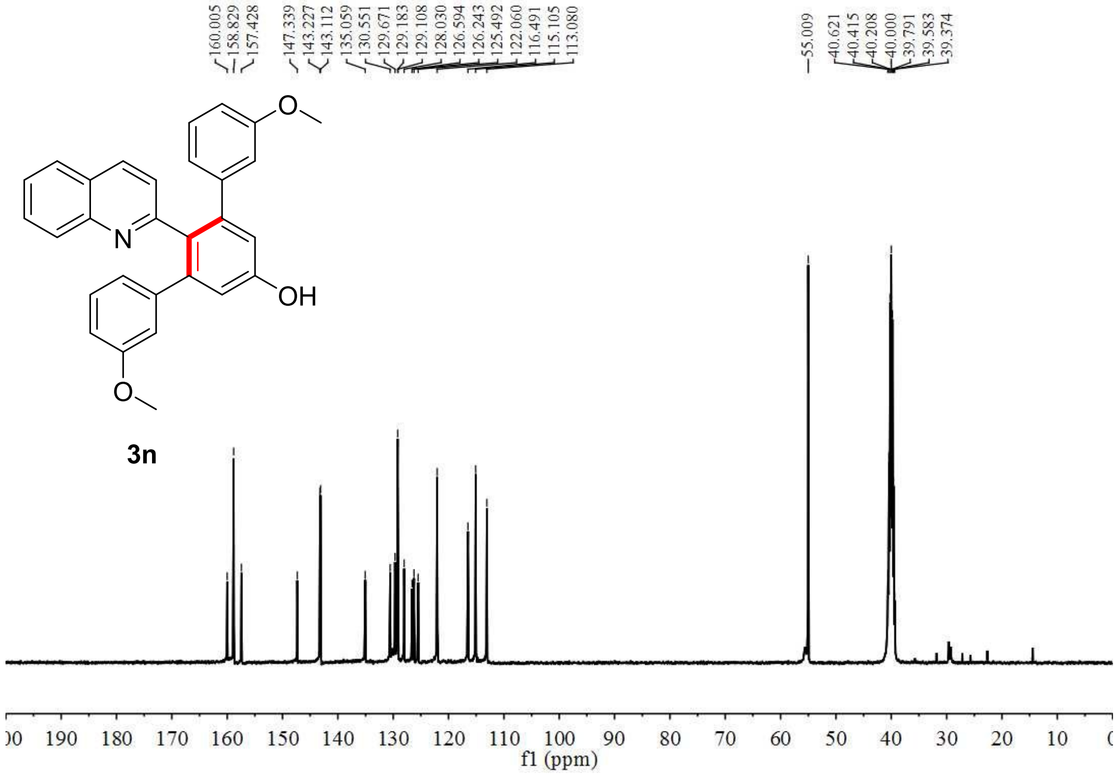
160.005 158.829 157.428
147.339 143.227 143.112 135.059 130.551 129.671 129.183 129.108 128.030 126.594 126.243 125.492 122.060 116.491 115.105 113.080
55.009
40.621 40.415 40.208 40.000 39.791 39.583 39.374
N
OH
3n
190 180 170 160 150 140 130 120 110 100 90 80 70 60 50 40 30 20 10
f1 (ppm)

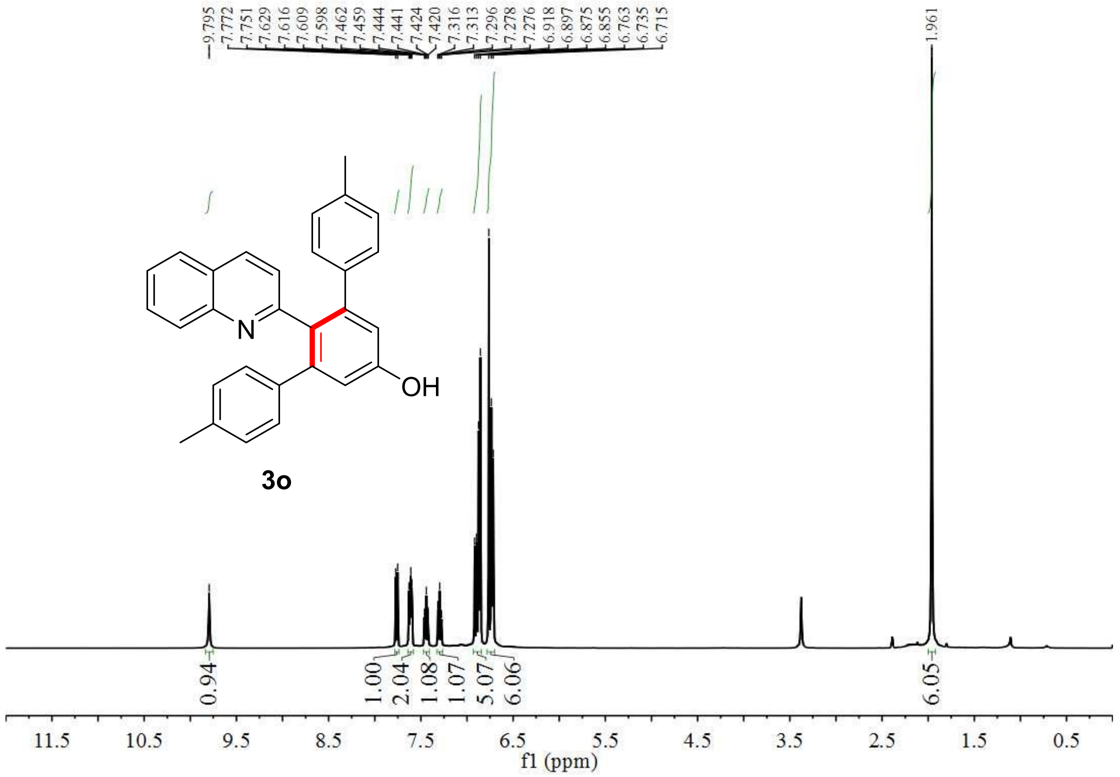
9.795 7.772 7.751 7.629 7.616 7.609 7.598 7.462 7.459 7.444 7.441 7.424 7.420 7.316 7.313 7.296 7.278 7.276 6.918 6.897 6.875 6.855 6.763 6.735 6.715
1.961
N
OH
3o
0.94
1.00 2.04 1.08 1.07 5.07 6.06
6.05
11.5 10.5 9.5 8.5 7.5 6.5 5.5 4.5 3.5 2.5 1.5 0.5
f1 (ppm)

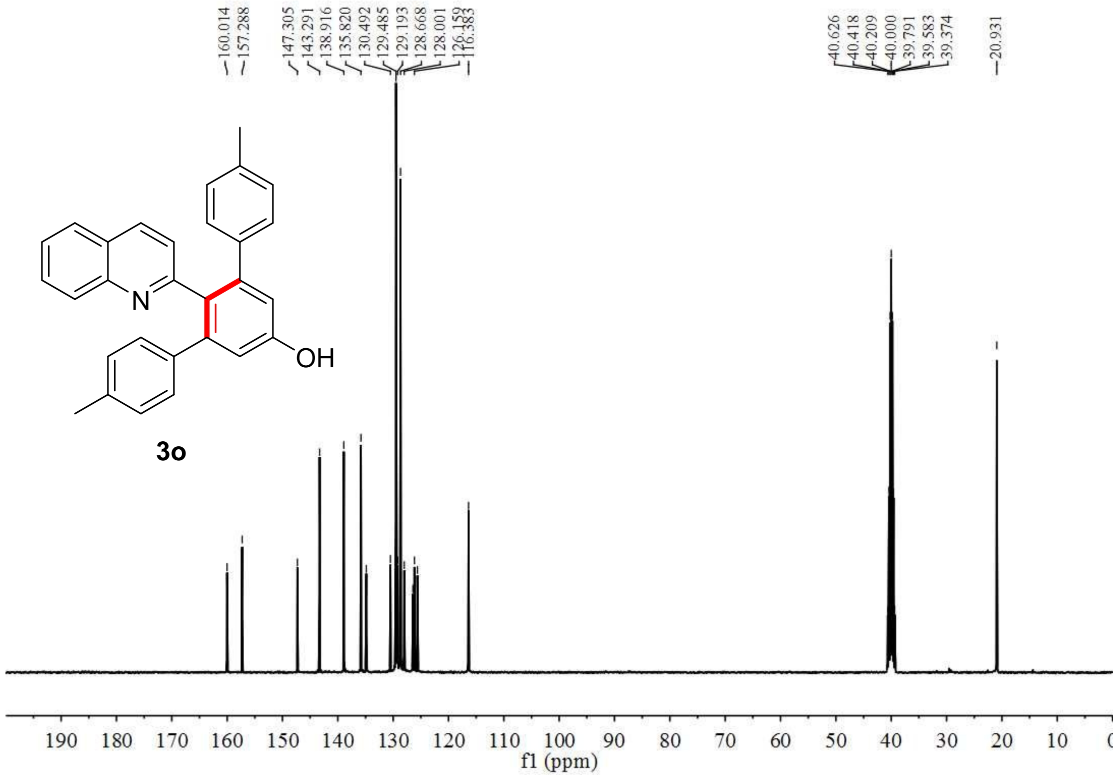
160.014 157.288
147.305 143.291 138.916 135.820 130.492 129.485 129.193 128.668 128.001 126.159 116.383
40.626 40.418 40.209 40.000 39.791 39.583 39.374
20.931
N
OH
3o
190 180 170 160 150 140 130 120 110 100 90 80 70 60 50 40 30 20 10
f1 (ppm)

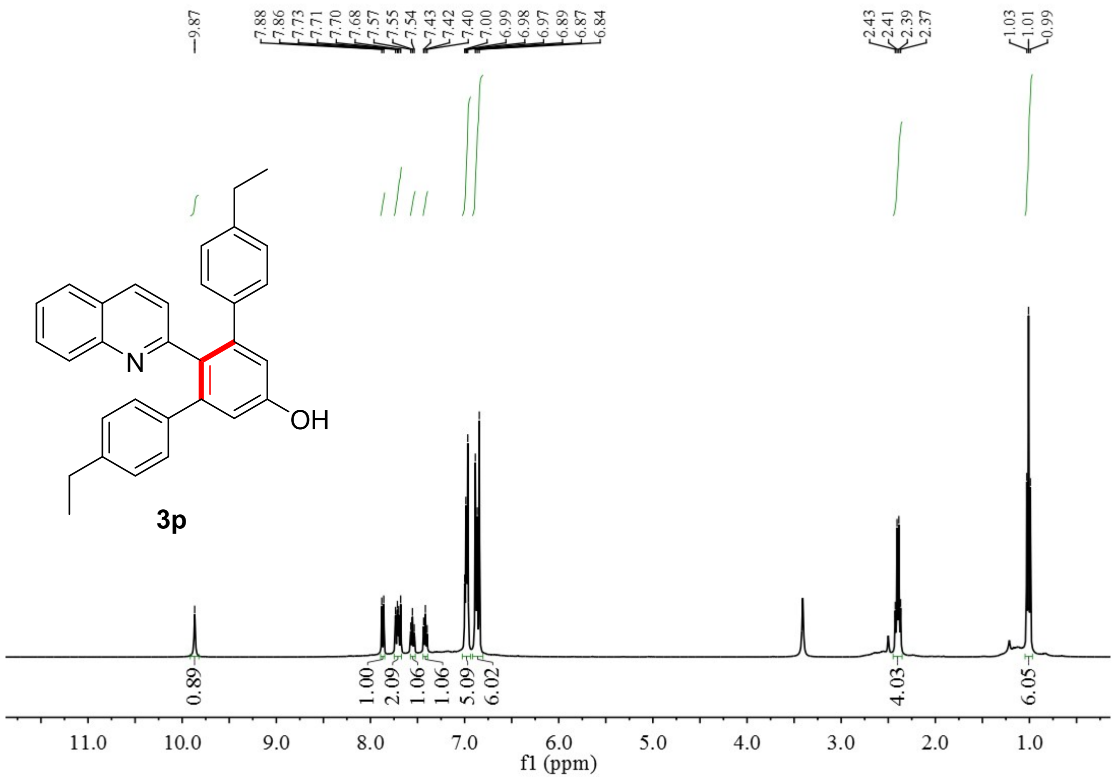

9.87
7.88
7.86
7.73
7.71
7.70
7.68
7.57
7.55
7.54
7.43
7.42
7.40
7.00
6.99
6.98
6.97
6.89
6.87
6.84
2.43
2.41
2.39
2.37
1.03
1.01
0.99
N
OH
3p
0.89
1.00
2.09
1.06
1.06
5.09
6.02
4.03
6.05
11.0
10.0
9.0
8.0
7.0
6.0
5.0
4.0
3.0
2.0
1.0
f1 (ppm)

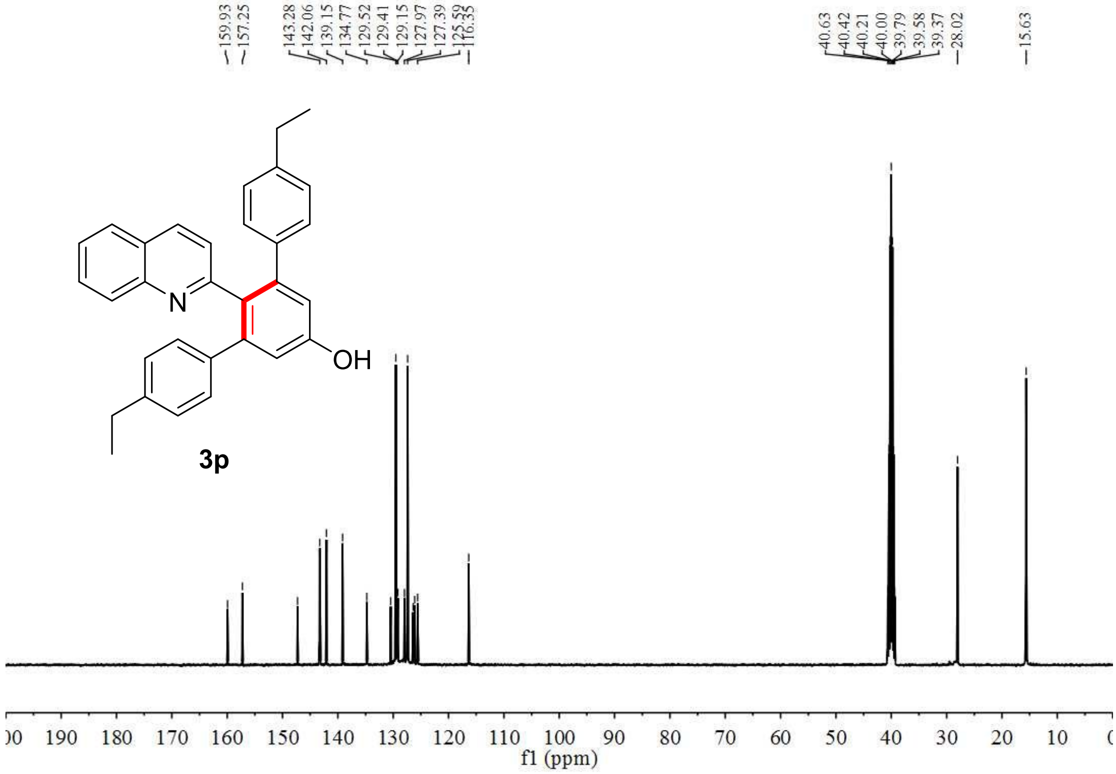

159.93
157.25
143.28
142.06
139.15
134.77
129.52
129.41
129.15
127.97
127.39
125.59
116.35
40.63
40.42
40.21
40.00
39.79
39.58
39.37
28.02
15.63
N
OH
3p
190
180
170
160
150
140
130
120
110
100
90
80
70
60
50
40
30
20
10
f1 (ppm)

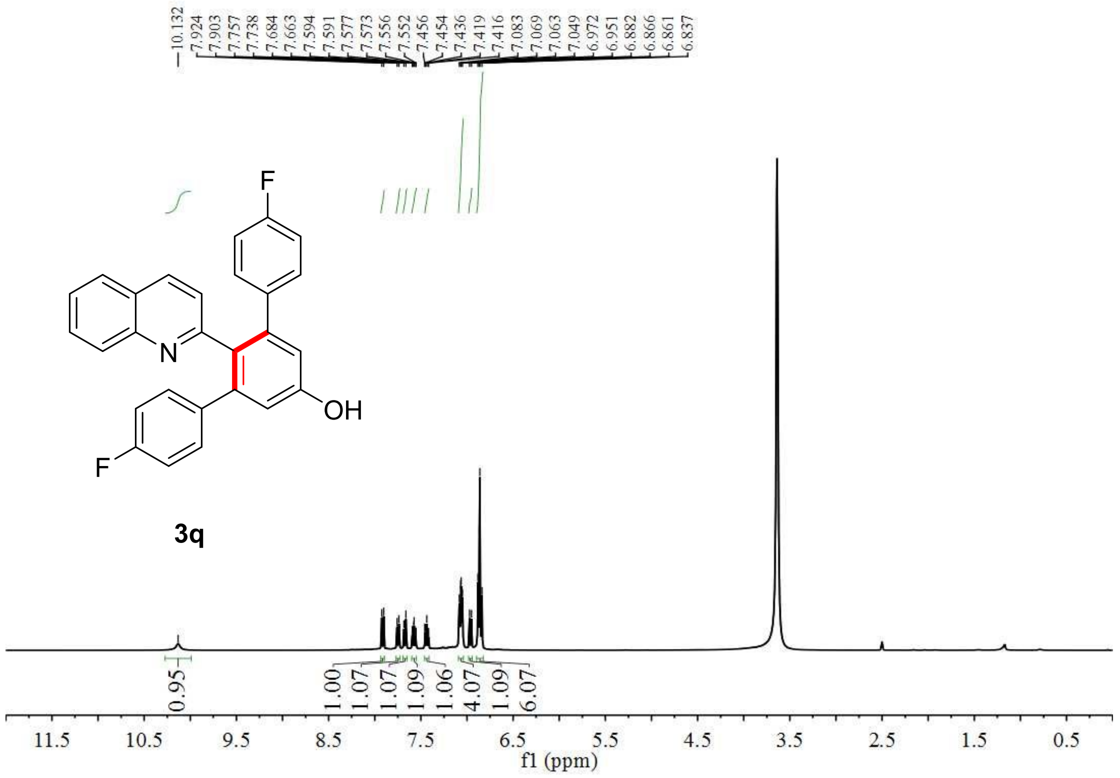
3q
f1 (ppm)

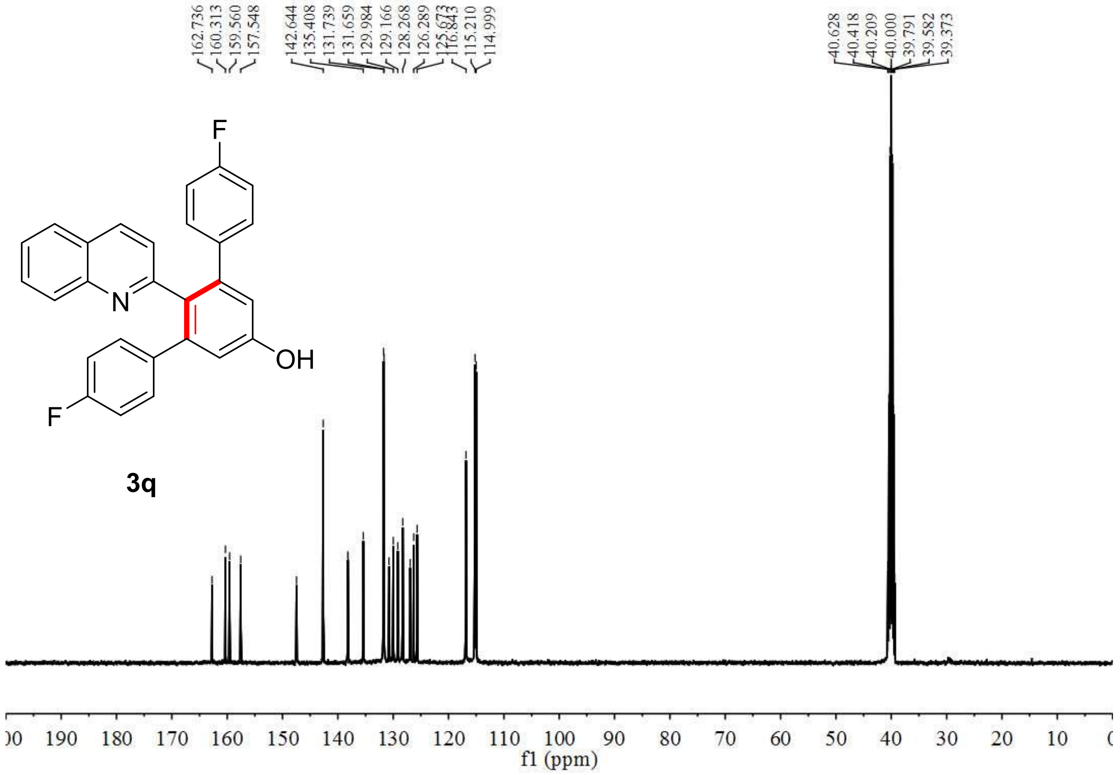
3q
f1 (ppm)

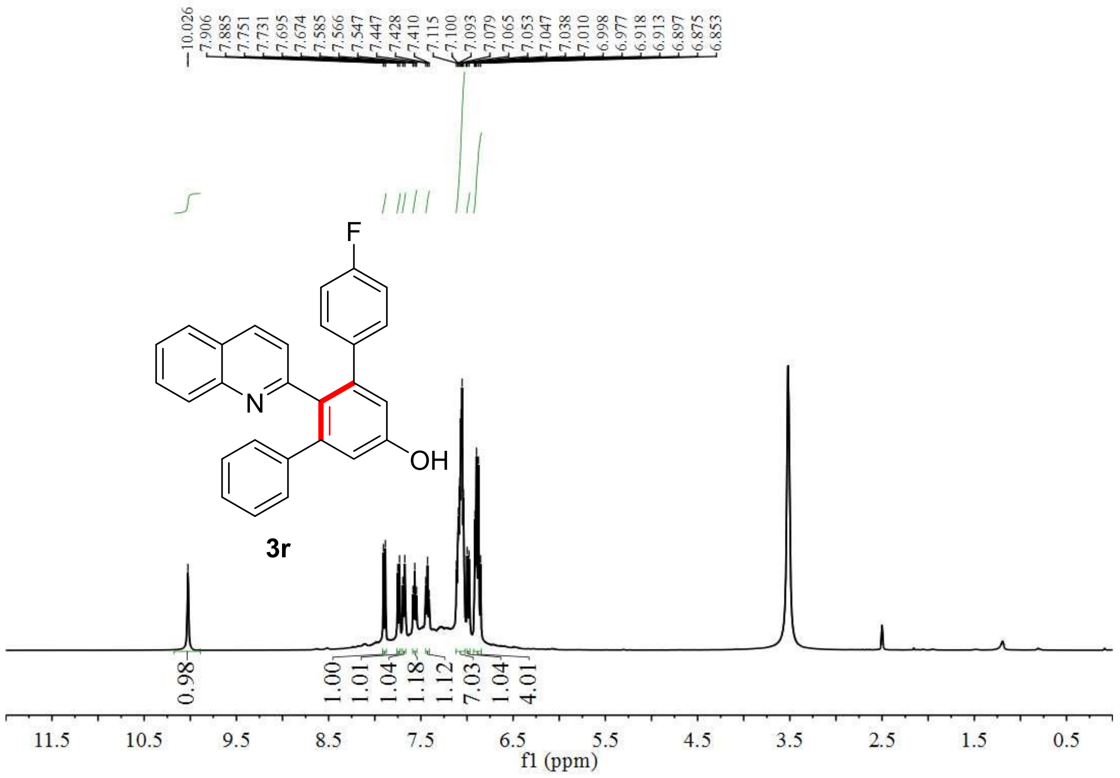

10.026
7.906
7.885
7.751
7.731
7.695
7.674
7.585
7.566
7.547
7.447
7.428
7.410
7.115
7.100
7.093
7.079
7.065
7.053
7.047
7.038
7.010
6.998
6.977
6.918
6.913
6.897
6.875
6.853
F
N
OH
3r
0.98
1.00
1.01
1.04
1.18
1.12
7.03
1.04
4.01
11.5 10.5 9.5 8.5 7.5 6.5 5.5 4.5 3.5 2.5 1.5 0.5
f1 (ppm)

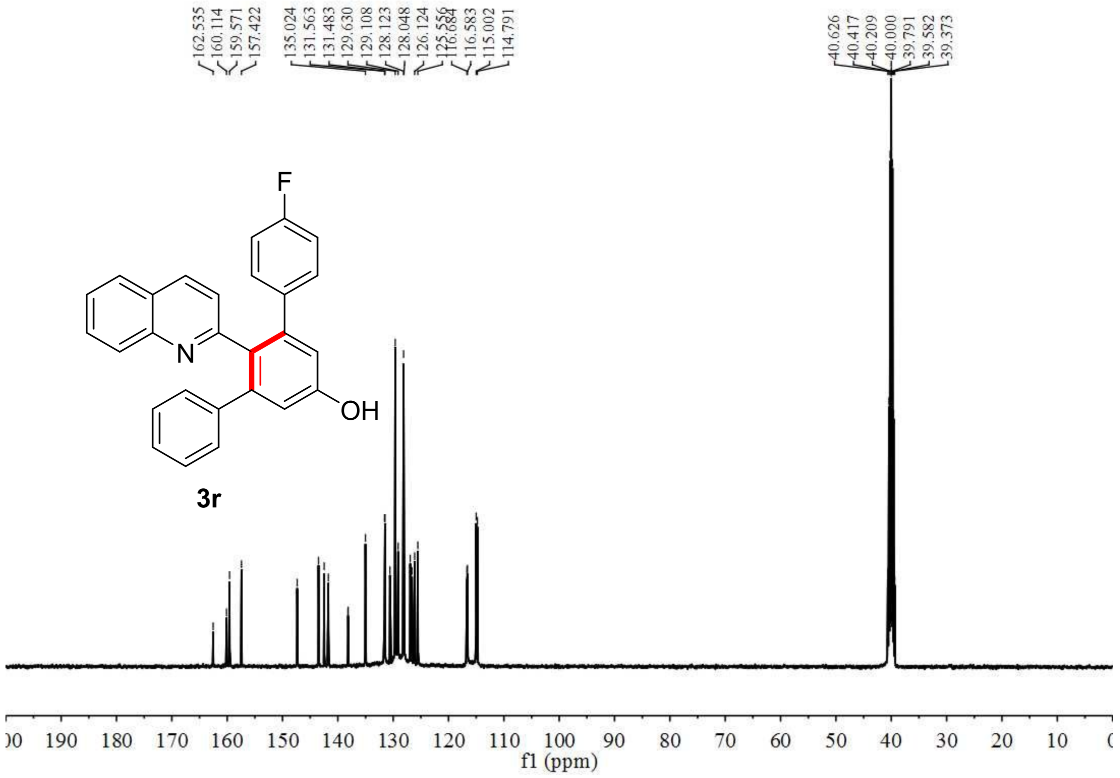

162.535
160.114
159.571
157.422
135.024
131.563
131.483
129.630
129.108
128.123
128.048
126.124
125.556
116.684
116.583
115.002
114.791
40.626
40.417
40.209
40.000
39.791
39.582
39.373
F
N
OH
3r
190 180 170 160 150 140 130 120 110 100 90 80 70 60 50 40 30 20 10
f1 (ppm)

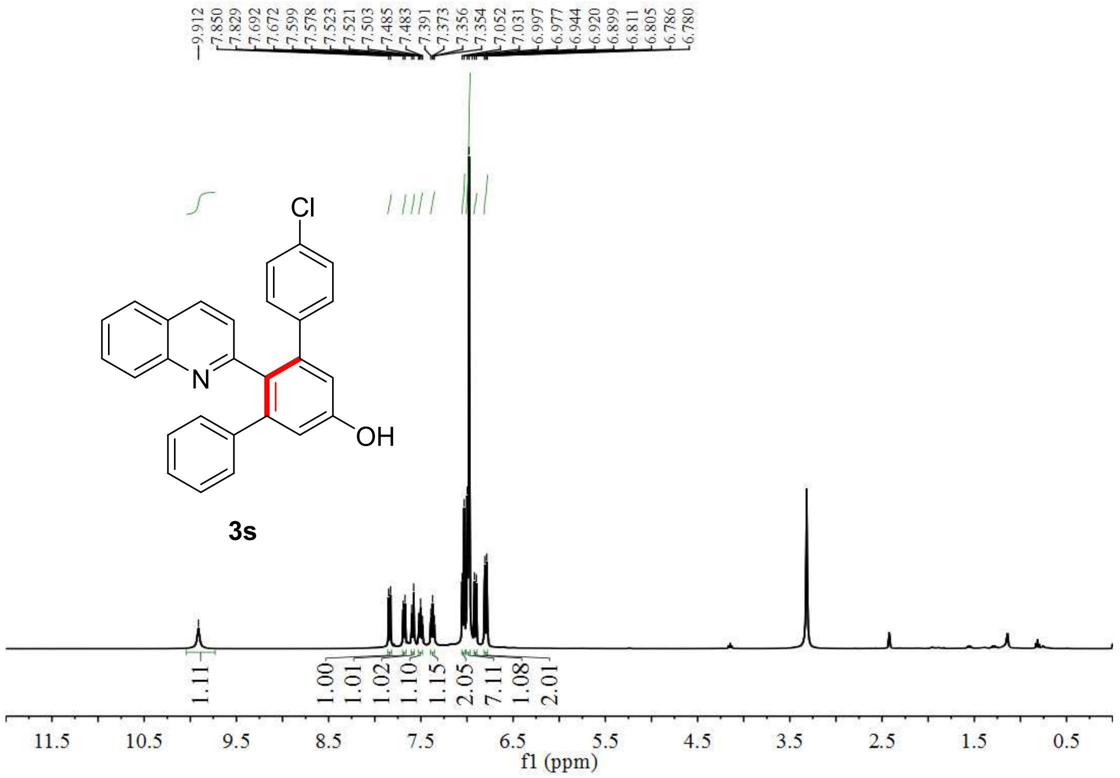
9.912
7.850
7.829
7.692
7.672
7.599
7.578
7.523
7.521
7.503
7.485
7.483
7.391
7.373
7.356
7.354
7.052
7.031
6.997
6.977
6.944
6.920
6.899
6.811
6.805
6.786
6.780
Cl
N
OH
3s
1.11
1.00
1.01
1.02
1.10
1.15
2.05
7.11
1.08
2.01
11.5
10.5
9.5
8.5
7.5
6.5
5.5
4.5
3.5
2.5
1.5
0.5
f1 (ppm)

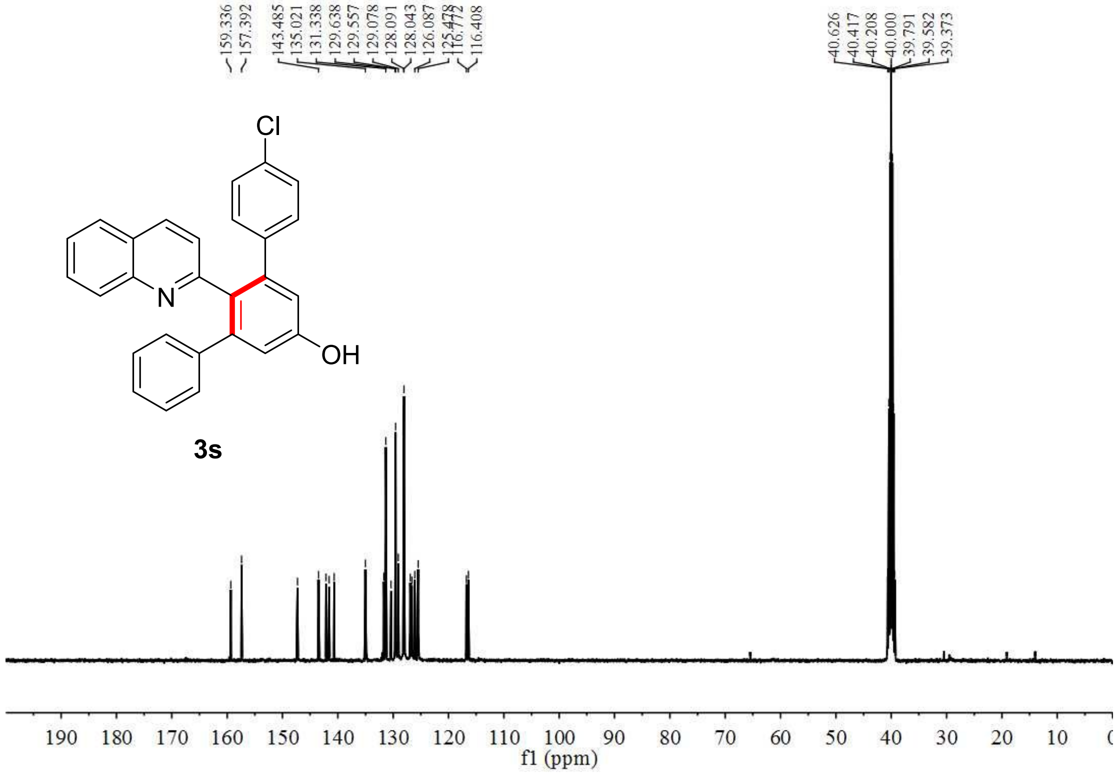
159.336
157.392
143.485
135.021
131.338
129.638
129.557
129.078
128.091
128.043
126.087
125.478
116.472
116.408
40.626
40.417
40.208
40.000
39.791
39.582
39.373
Cl
N
OH
3s
190
180
170
160
150
140
130
120
110
100
90
80
70
60
50
40
30
20
10
f1 (ppm)

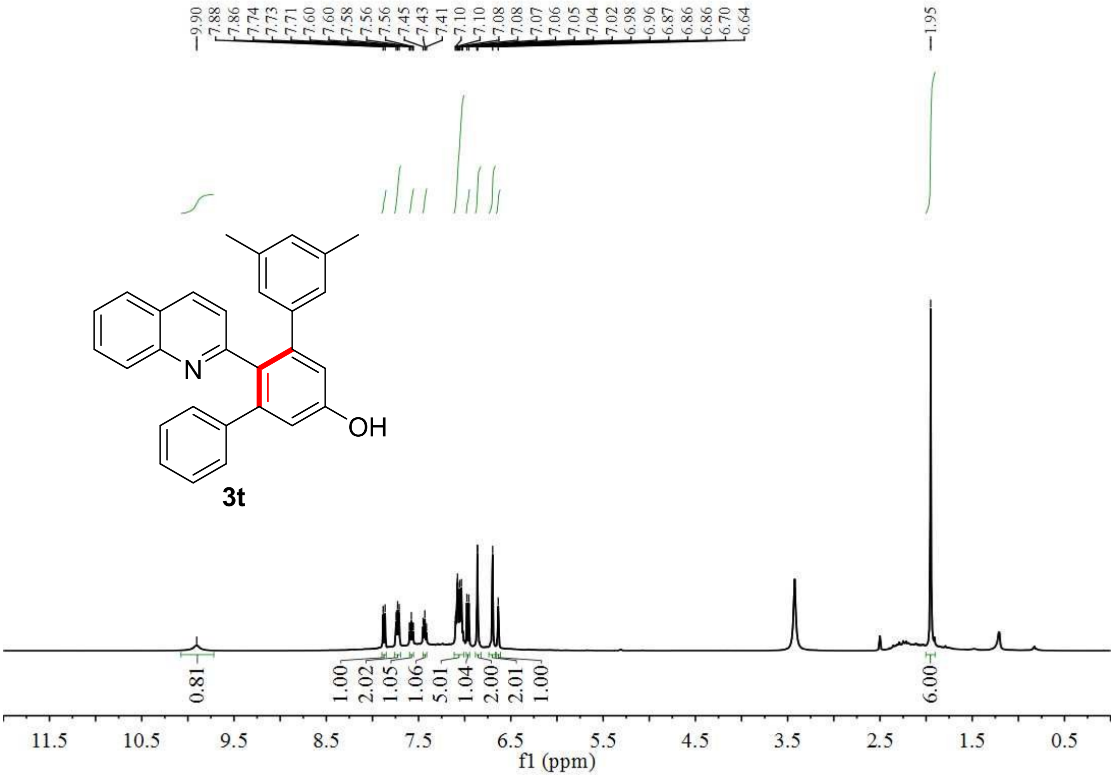
9.90 7.88 7.86 7.74 7.73 7.71 7.60 7.60 7.58 7.56 7.56 7.45 7.43 7.41 7.10 7.10 7.08 7.08 7.07 7.06 7.05 7.04 7.02 6.98 6.96 6.87 6.86 6.86 6.70 6.64
1.95
N
OH
3t
0.81
1.00 2.02 1.05 1.06 5.01 1.04 2.00 2.01 1.00
6.00
11.5 10.5 9.5 8.5 7.5 6.5 5.5 4.5 3.5 2.5 1.5 0.5
f1 (ppm)

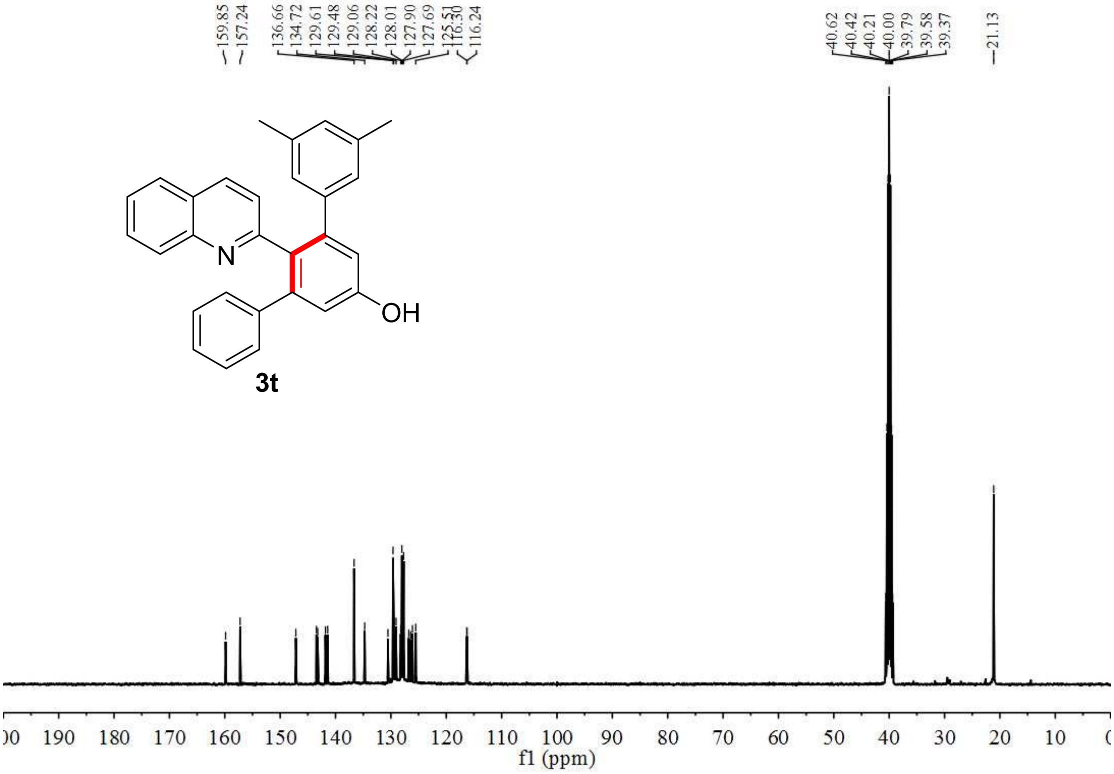
159.85 157.24
136.66 134.72 129.61 129.48 129.06 128.22 128.01 127.90 127.69 125.51 116.30 116.24
40.62 40.42 40.21 40.00 39.79 39.58 39.37
21.13
N
OH
3t
190 180 170 160 150 140 130 120 110 100 90 80 70 60 50 40 30 20 10
f1 (ppm)

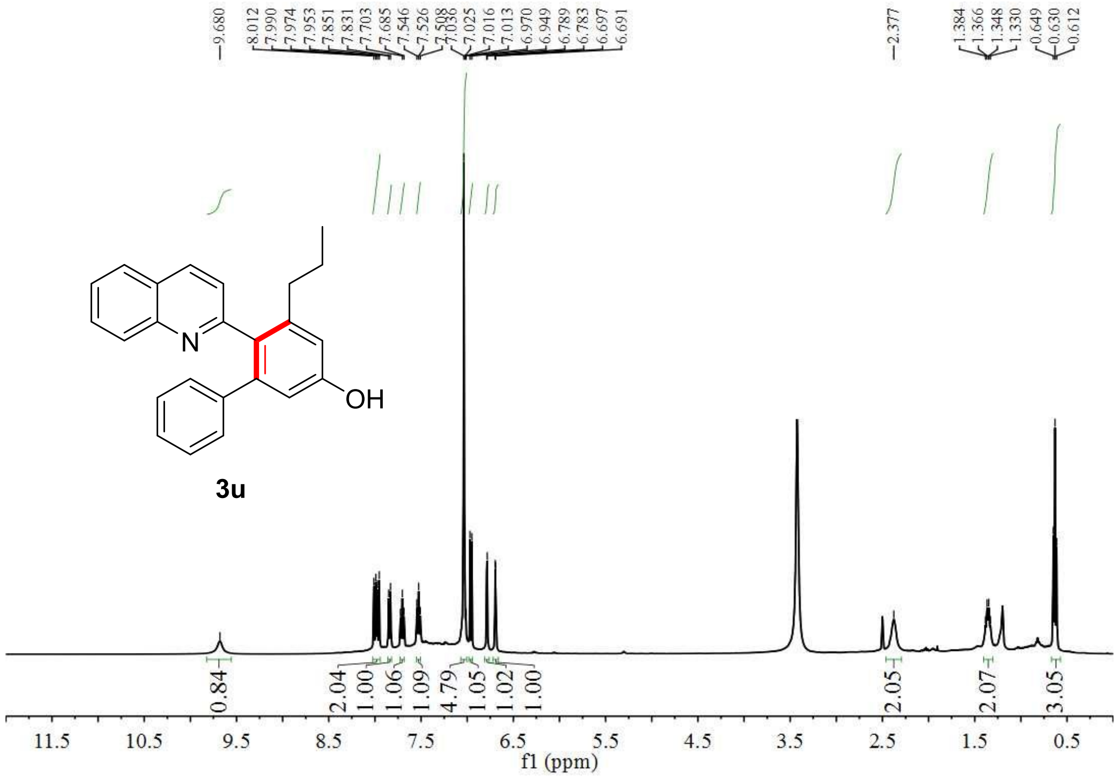
9.680
8.012 7.990 7.974 7.953 7.851 7.831 7.703 7.685 7.546 7.526 7.508 7.036 7.025 7.016 7.013 6.970 6.949 6.789 6.783 6.697 6.691
2.377
1.384 1.366 1.348 1.330 0.649 0.630 0.612
N
OH
3u
0.84 2.04 1.00 1.06 1.09 4.79 1.05 1.02 1.00 2.05 2.07 3.05
11.5 10.5 9.5 8.5 7.5 6.5 5.5 4.5 3.5 2.5 1.5 0.5
f1 (ppm)

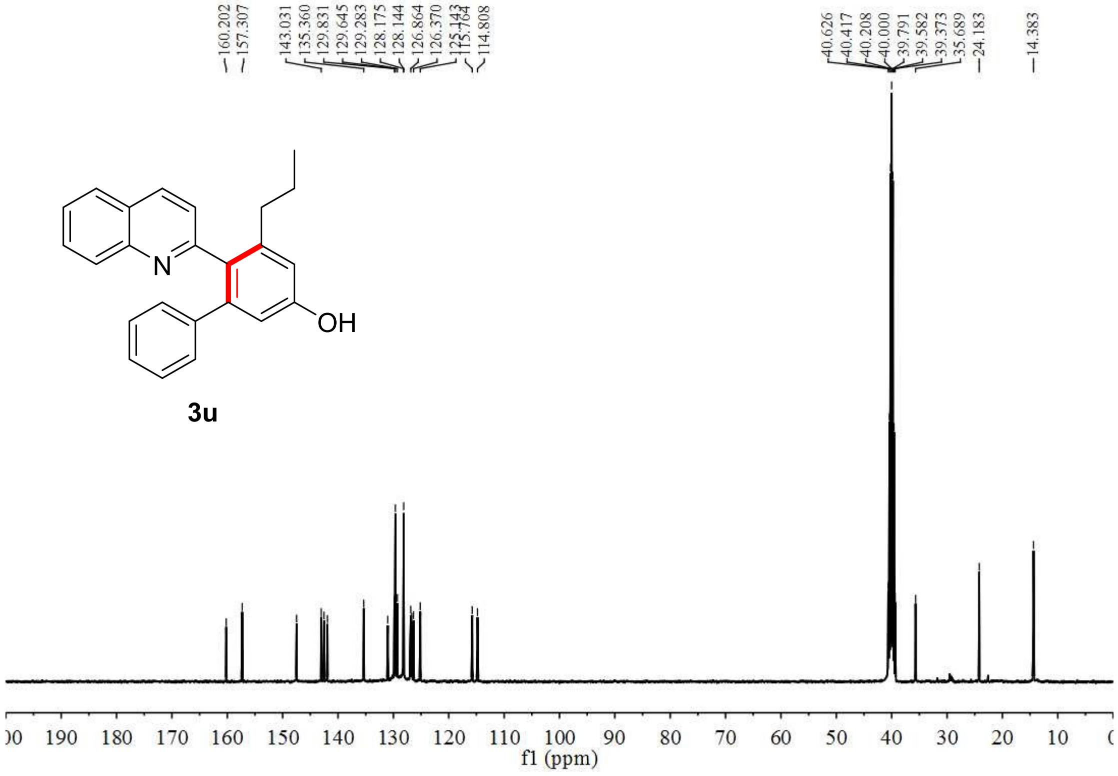
160.202 157.307
143.031 135.360 129.831 129.645 129.283 128.175 128.144 126.864 126.370 125.143 115.764 114.808
40.626 40.417 40.208 40.000 39.791 39.582 39.373 35.689 24.183
14.383
N
OH
3u
190 180 170 160 150 140 130 120 110 100 90 80 70 60 50 40 30 20 10
f1 (ppm)

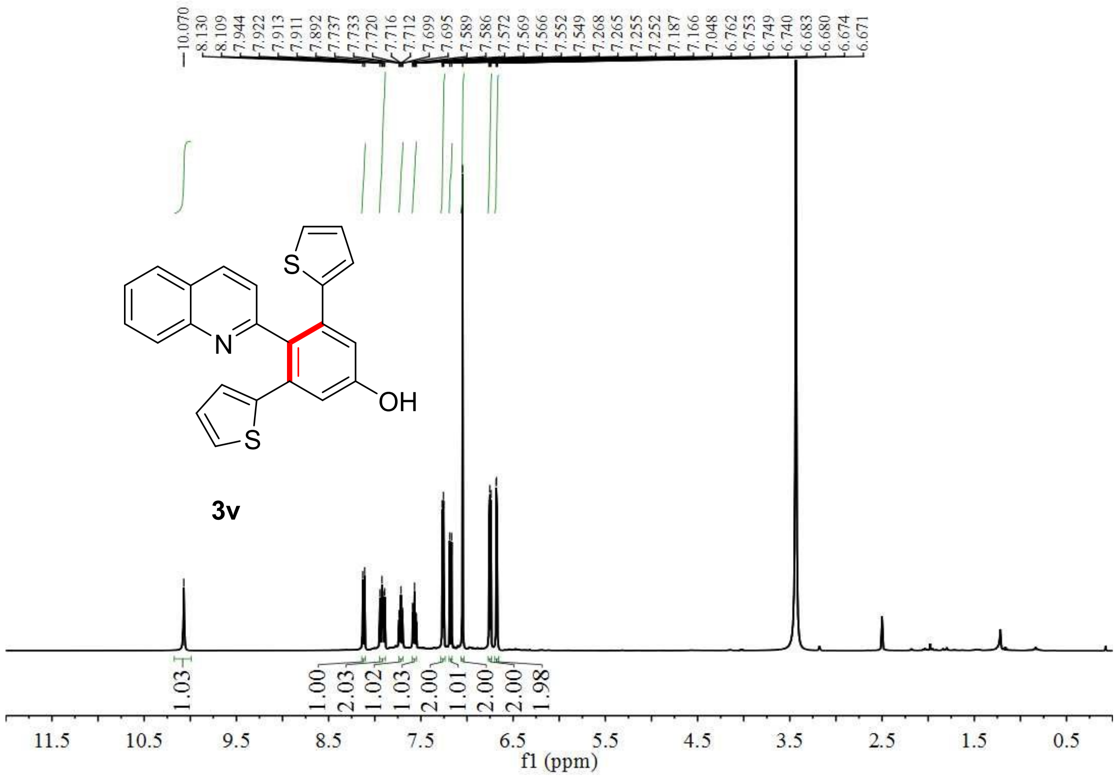
10.070
8.130
8.109
7.944
7.922
7.913
7.911
7.892
7.737
7.733
7.720
7.716
7.712
7.699
7.695
7.589
7.586
7.572
7.569
7.566
7.552
7.549
7.268
7.265
7.255
7.252
7.187
7.166
7.048
6.762
6.753
6.749
6.740
6.683
6.680
6.674
6.671
N
S
S
OH
1.03
1.00
2.03
1.02
1.03
2.00
1.01
2.00
2.00
1.98
11.5
10.5
9.5
8.5
7.5
6.5
5.5
4.5
3.5
2.5
1.5
0.5
f1 (ppm)

3v

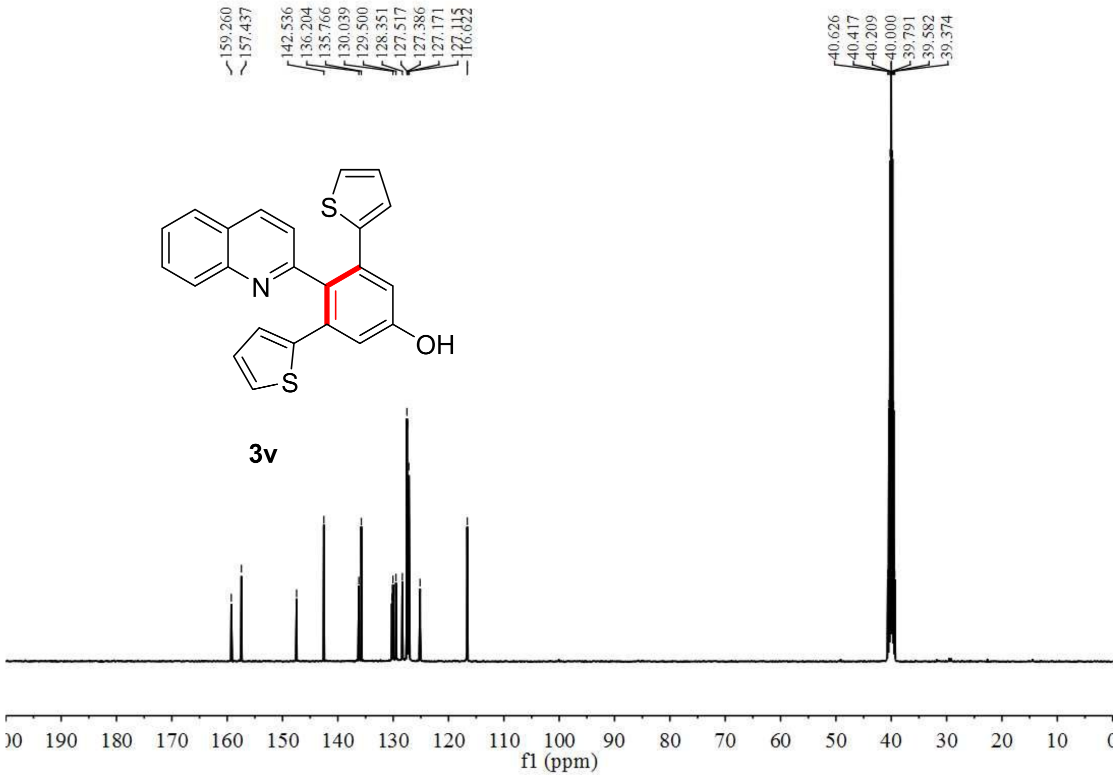
159.260
157.437
142.536
136.204
135.766
130.039
129.500
128.351
127.517
127.386
127.171
127.115
116.622
40.626
40.417
40.209
40.000
39.791
39.582
39.374
N
S
S
OH
190
180
170
160
150
140
130
120
110
100
90
80
70
60
50
40
30
20
10
f1 (ppm)

3v

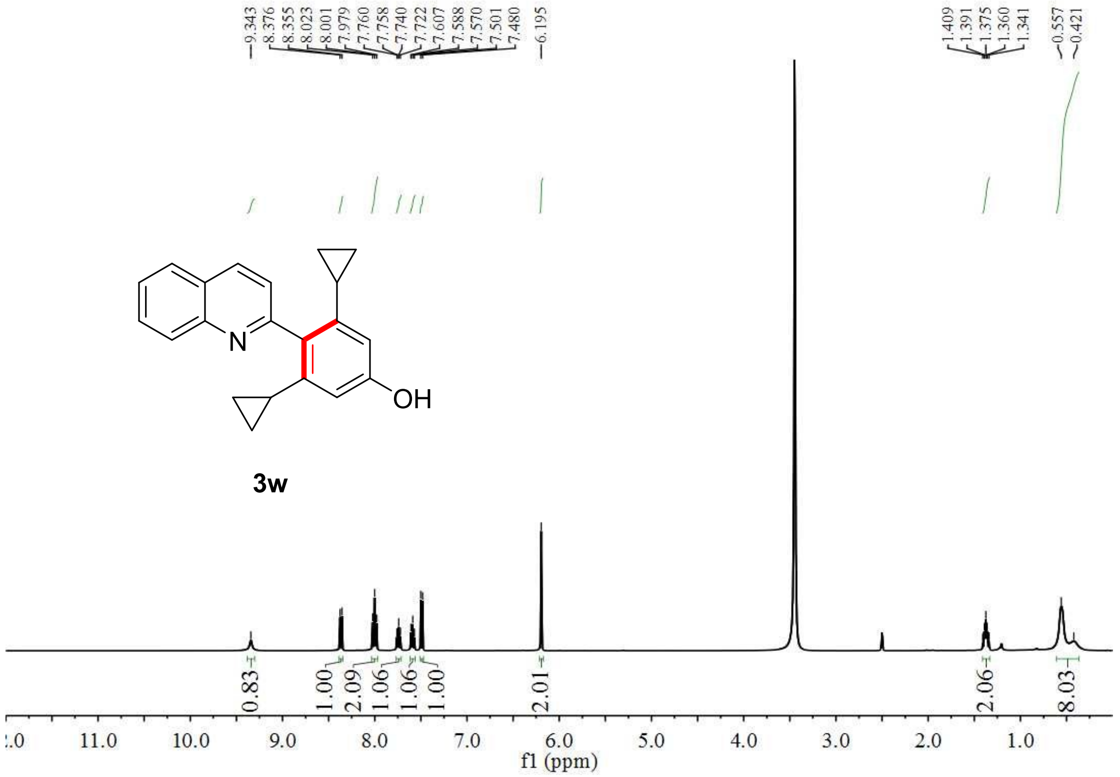
9.343
8.376
8.355
8.023
8.001
7.979
7.760
7.758
7.740
7.722
7.607
7.588
7.570
7.501
7.480
6.195
1.409
1.391
1.375
1.360
1.341
0.557
0.421
N
OH
3w
0.83
1.00
2.09
1.06
1.06
1.00
2.01
2.06
8.03
f1 (ppm)

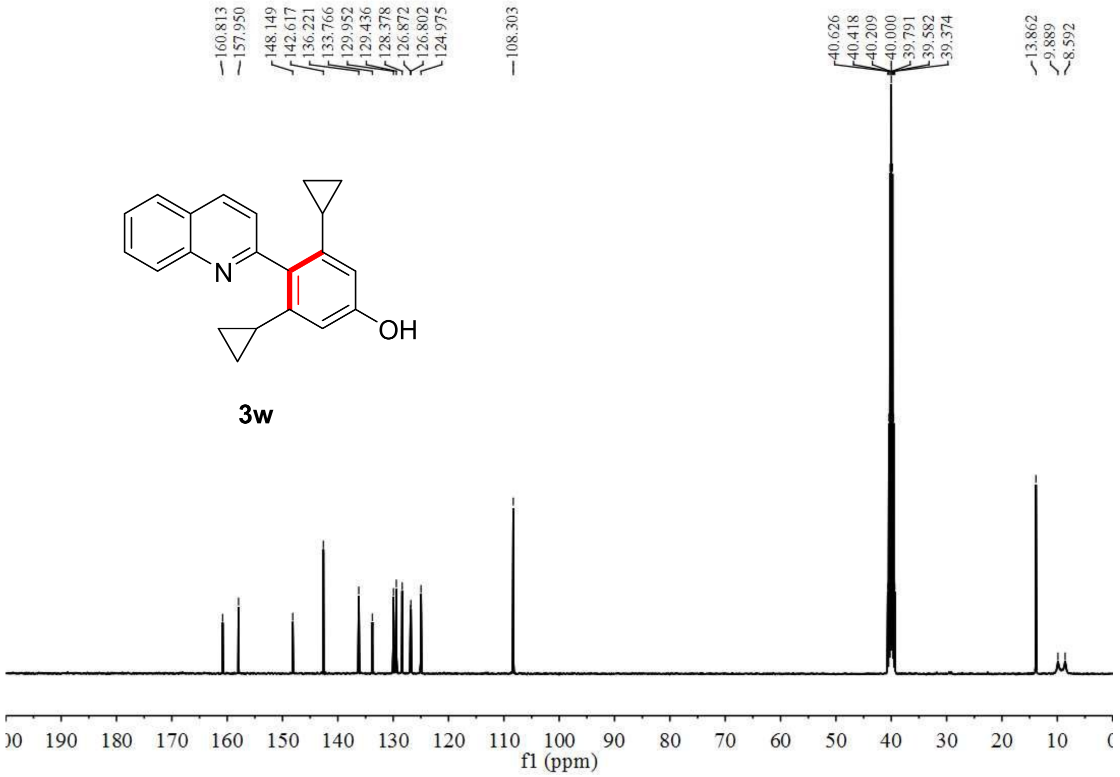
160.813
157.950
148.149
142.617
136.221
133.766
129.952
129.436
128.378
126.872
126.802
124.975
108.303
40.626
40.418
40.209
40.000
39.791
39.582
39.374
13.862
9.889
8.592
N
OH
3w
f1 (ppm)

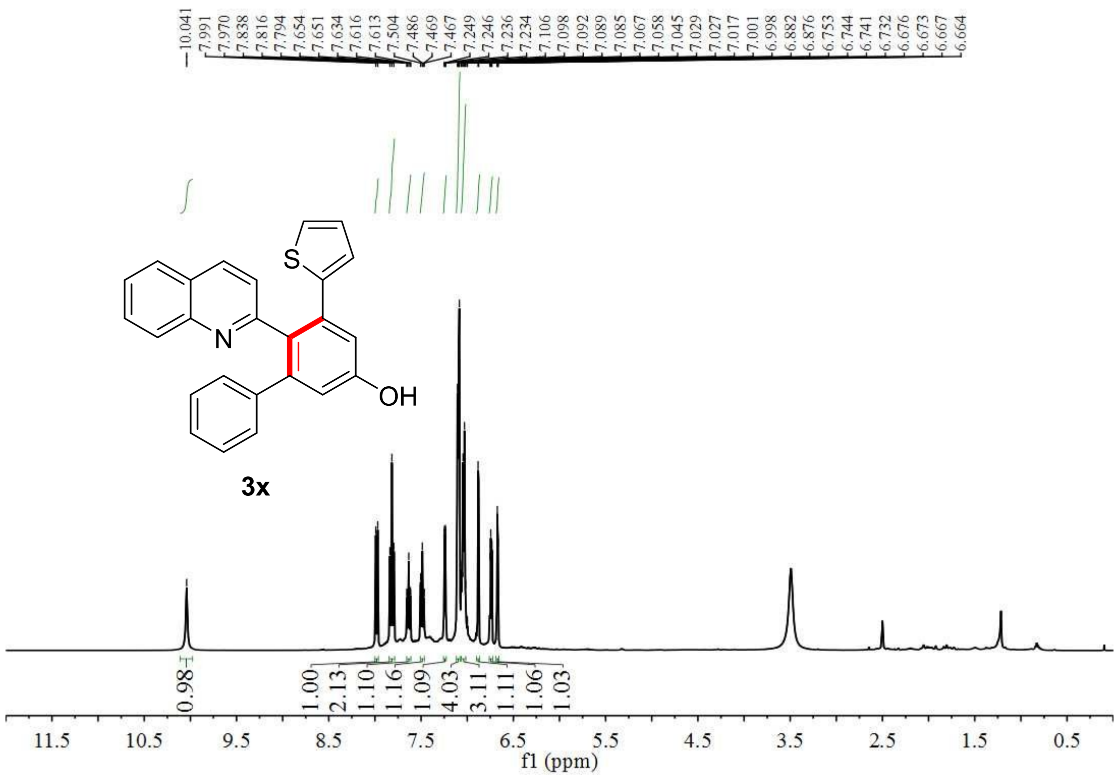

10.041
7.991
7.970
7.838
7.816
7.794
7.654
7.651
7.634
7.616
7.613
7.504
7.486
7.469
7.467
7.249
7.246
7.236
7.234
7.106
7.098
7.092
7.089
7.085
7.067
7.058
7.045
7.029
7.027
7.017
7.001
6.998
6.882
6.876
6.753
6.744
6.741
6.732
6.676
6.673
6.667
6.664
S
N
OH
3x
0.98
1.00
2.13
1.10
1.16
1.09
4.03
3.11
1.11
1.06
1.03
11.5
10.5
9.5
8.5
7.5
6.5
5.5
4.5
3.5
2.5
1.5
0.5
f1 (ppm)

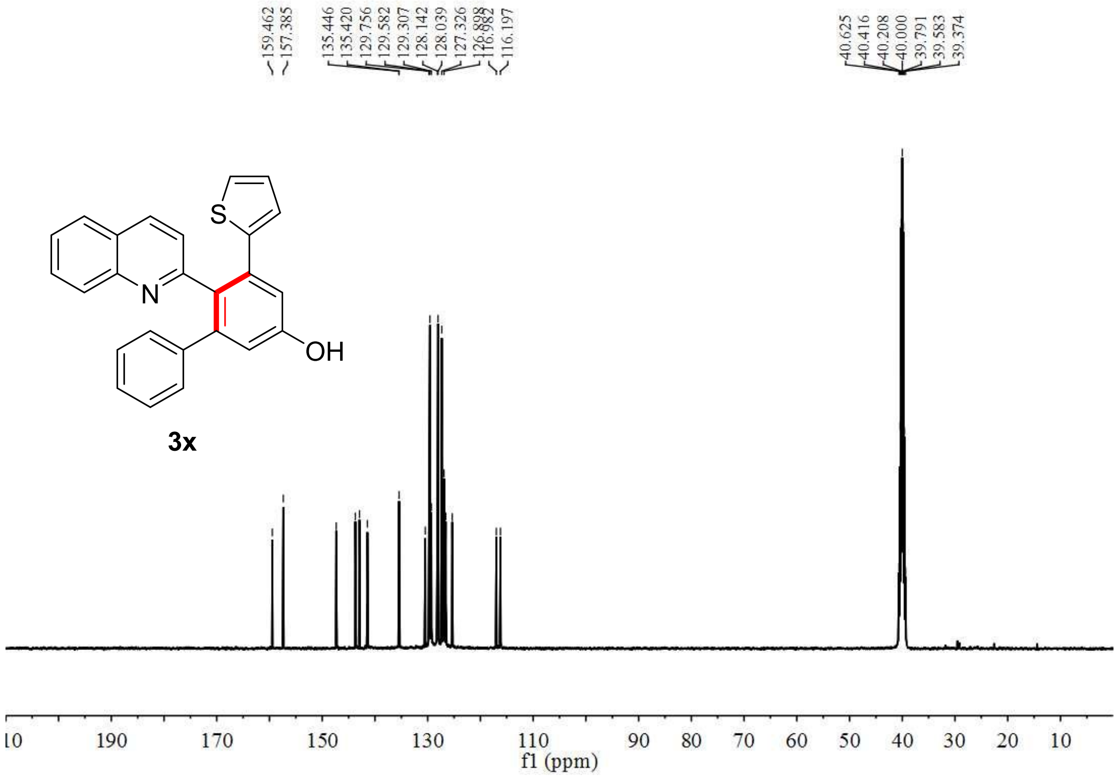

159.462
157.385
135.446
135.420
129.756
129.582
129.307
128.142
128.039
127.326
116.197
40.625
40.416
40.208
40.000
39.791
39.583
39.374
S
N
OH
3x
190
170
150
130
110
90
80
70
60
50
40
30
20
10
f1 (ppm)

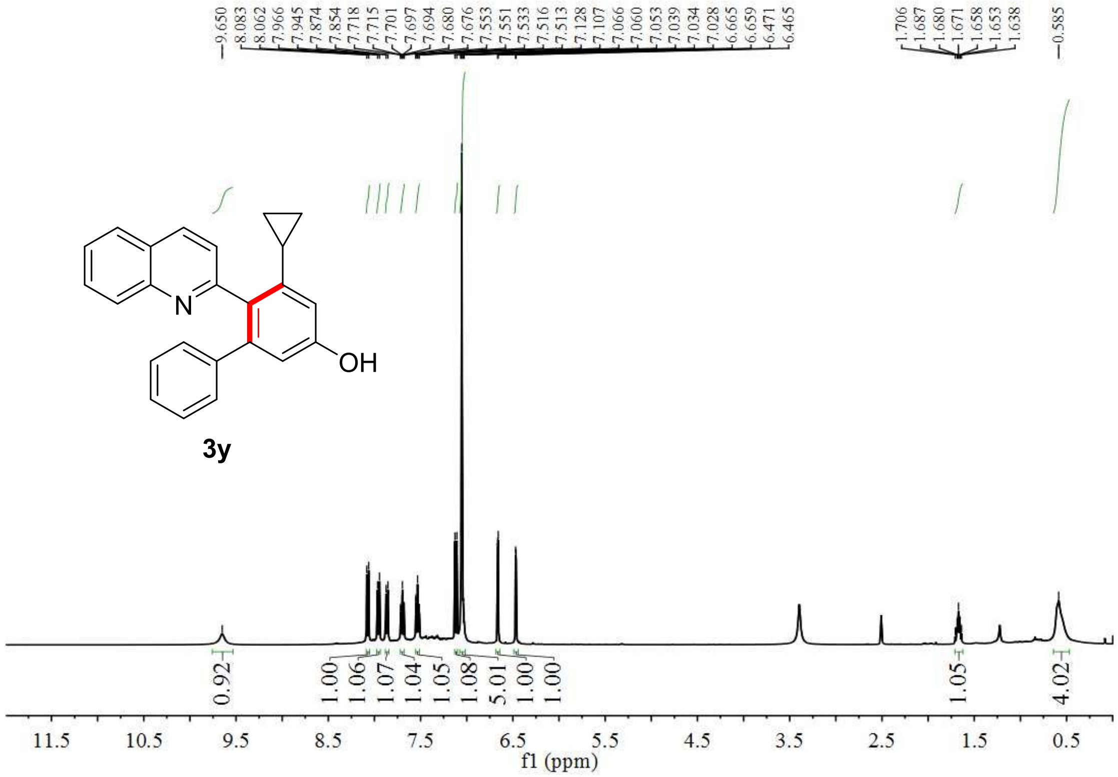
9.650
8.083 8.062 7.966 7.945 7.874 7.854 7.718 7.715 7.701 7.697 7.694 7.680 7.676 7.553 7.551 7.533 7.516 7.513 7.128 7.107 7.066 7.060 7.053 7.039 7.034 7.028 6.665 6.659 6.471 6.465
1.706 1.687 1.680 1.671 1.658 1.653 1.638
0.585
N
OH
3y
0.92
1.00 1.06 1.07 1.04 1.05 1.08 5.01 1.00 1.00
1.05
4.02
11.5 10.5 9.5 8.5 7.5 6.5 5.5 4.5 3.5 2.5 1.5 0.5
f1 (ppm)

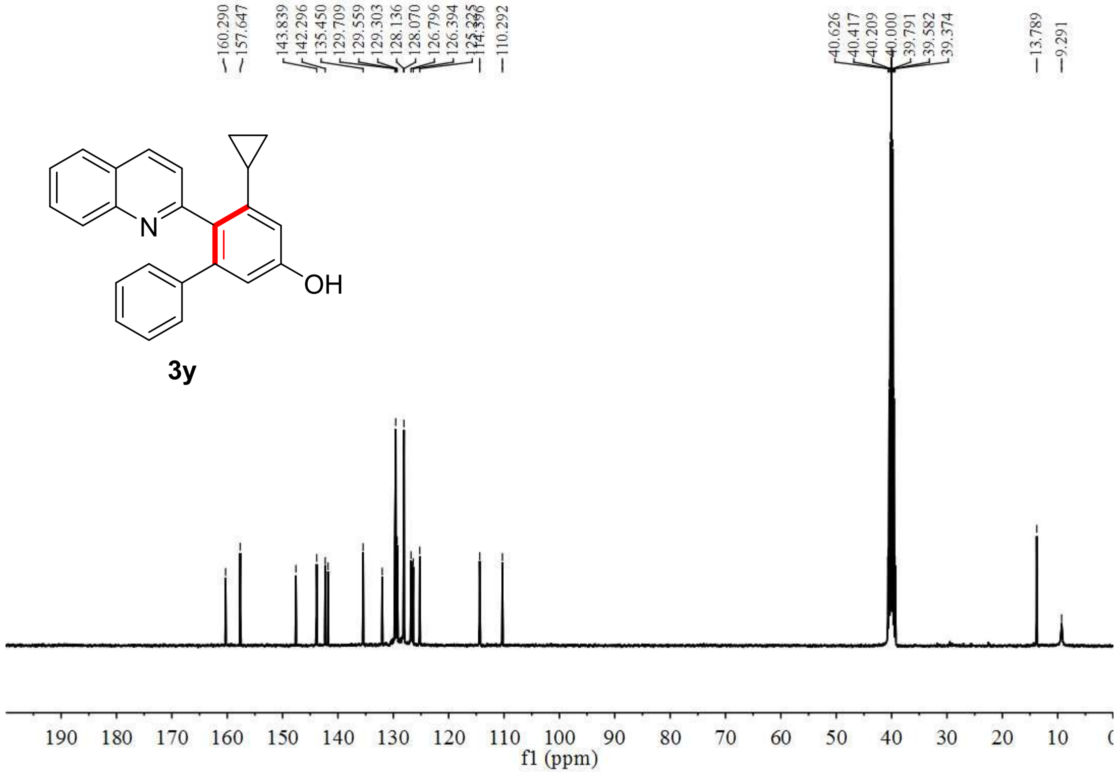
160.290
157.647
143.839 142.296 135.450 129.709 129.559 129.303 128.136 128.070 126.796 126.394 125.325 114.396
110.292
40.626 40.417 40.209 40.000 39.791 39.582 39.374
13.789
9.291
N
OH
3y
190 180 170 160 150 140 130 120 110 100 90 80 70 60 50 40 30 20 10
f1 (ppm)

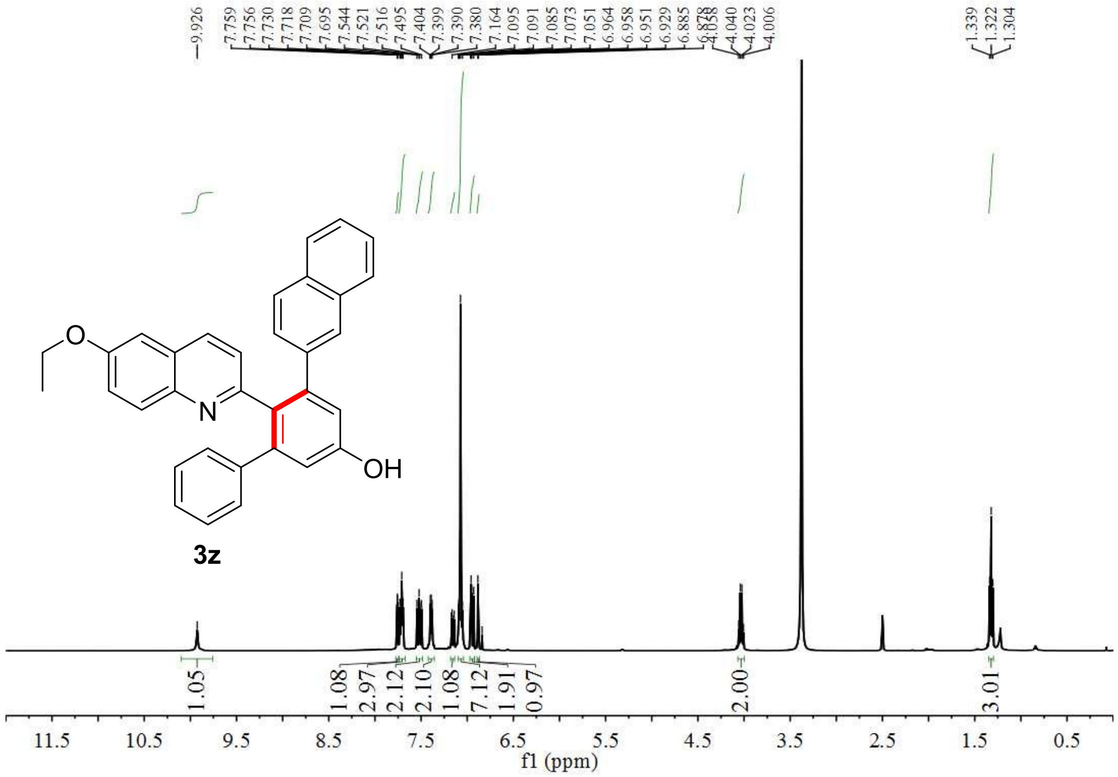
3z
f1 (ppm)

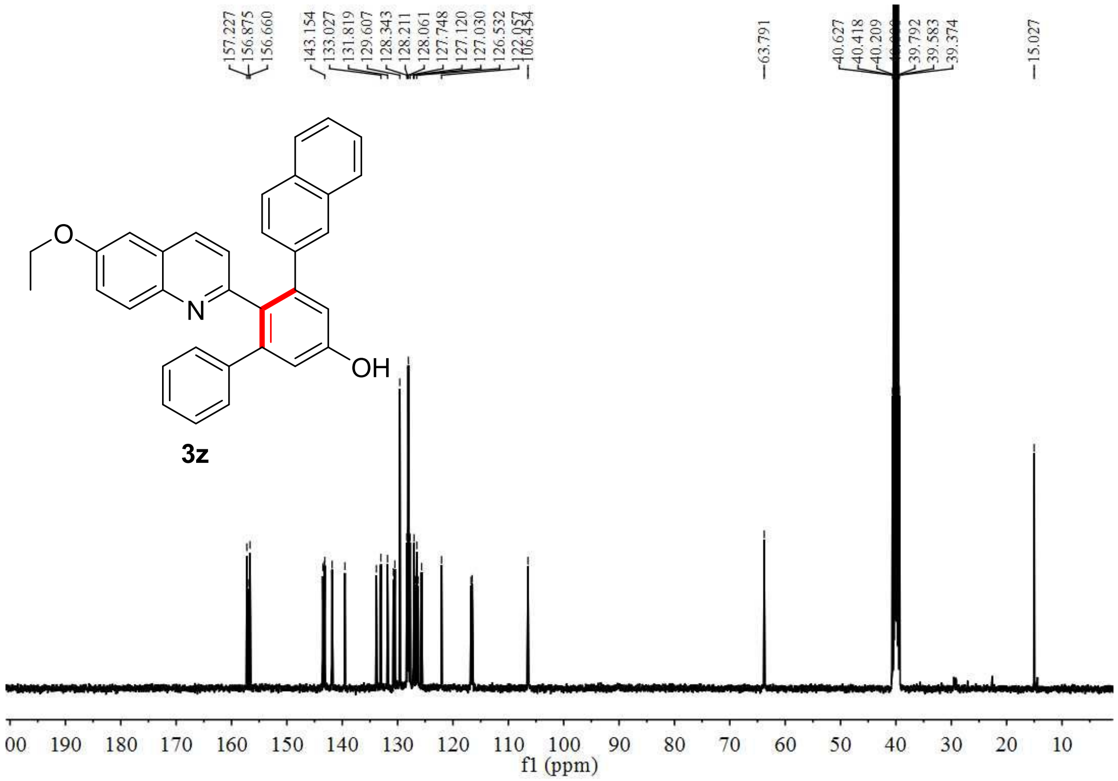
3z
f1 (ppm)
